# Impact of violence on emotional exhaustion risk of registered nurses in Germany: a Bayesian analysis of cross-sectional data with multiple imputations

**DOI:** 10.1186/s12912-025-03745-y

**Published:** 2025-08-18

**Authors:** Alessandro Campione, Cornelia Henschke

**Affiliations:** 1 Department of Health Care Management, Berlin Centre of Health Economics Research (BerlinHECOR), Technische Universität Berlin, H80, Straße des 17. Juni 135, 10623 Berlin, Germany; 2 Institute of General Practice and Interprofessional Care, University Hospital Tübingen, Eberhard Karls Universität Tübingen, Tübingen, Germany

**Keywords:** Nursing, Occupational health, Workplace violence, Workplace safety, Nursing service delivery, Working conditions, Human resources, Workforce retention

## Abstract

**Background:**

Medical workers are at risk of burnout due to various stressors, affecting their health, reducing quality of care, and threatening the retention of a resilient workforce. Among these stressors, workplace violence has emerged as a contributor to an increased risk of burnout among nurses. However, evidence from the German context remains limited. This study investigates the association between the frequency of workplace violence and the risk of the primary dimension of burnout, emotional exhaustion.

**Methods:**

This analysis is based on data from a German survey of registered nurses, measuring burnout risk and experiences of workplace violence. Multiple imputations were applied to missing data. The primary outcome, emotional exhaustion, was assessed with the Maslach Burnout Inventory. Extensive literature searches guided variable selection and prior elicitation for ordinal Bayesian regression. The main independent variables measured self-reported frequencies of physical and verbal violence from different perpetrators.

**Results:**

A total of 4317 RNs responded to the survey. Multiple imputed estimates revealed a high prevalence of frequent verbal (21.6%) and physical (3.8%) violence by patients and their families. High frequency verbal and physical violence perpetrated by staff was reported by 7.8% and 9.7% of respondents, respectively. Bayesian regression analyses across multiple imputed datasets showed a robust association between all types of workplace violence and increased risk of emotional exhaustion.

**Conclusion:**

Repeated exposure to physical and verbal violence is a major contributor to the risk of burnout in the emotional exhaustion dimension among German RNs. Given its high prevalence, there is an urgent need for reforms and policy measures to prevent violence and address its impact. Updated evidence and targeted interventions are essential to safeguard workers and maintain a resilient nursing workforce.

**Clinical trial number:**

Not applicable.

**Supplementary Information:**

The online version contains supplementary material available at 10.1186/s12912-025-03745-y.

## Introduction

Burnout poses a threat to the sustainable retention, effectiveness, and health of an already scarce healthcare workforce. It has been described as one of the primary health concerns for professions involved in interpersonal work [1]. As an occupational outcome, burnout negatively affects patient safety, quality of care, patient satisfaction, and the incidence of medical errors among nurses [2]. Initially conceptualized in the context of human services occupations, burnout was defined as a state of exhaustion and fatigue [3]. While early empirical research focused on human services, the concept of burnout has been expanded to include all occupations [4]. As a result, the definition has shifted from a narrow focus on physical depletion to a more comprehensive psychological construct [5].

Burnout is now included in the 11th revision of the International Classification of Diseases (ICD-11), where it is defined as “[…] a syndrome conceptualized as resulting from chronic workplace stress that has not been successfully managed.”. However, it is not classified as a medical condition [6]. The most widely used measurement tool is the Maslach-Burnout-Inventory, with emotional exhaustion (EE) identified as the primary dimension related to job demands and stress [7–9]. Stress is characterized as a relational and dynamic process between the individual and their environment, in which a situation is perceived as demanding and exceeding personal coping resources [10, 11]. When overwhelmed or chronically activated, as in prolonged occupational strain, this process can lead to maladaptive emotional, behavioral and neuroendocrine responses in the long term [12, 13].

Compared to the general population, healthcare workers are at higher risk of burnout, with an estimated prevalence of 30% (CI: 26–34%) in nurses, as reported in a meta-analysis of more than 30 countries covering 10 years. The analysis followed a three-factor approach, defining a high risk of burnout when two or more MBI dimensions were assessed as severe [14]. EE is particularly prevalent, affecting 48% (CI: 33–63%) of intensive care and emergency department nurses and physicians [15], and 25% (CI: 17–35%) of mental health nurses [16]. Several studies have assessed various factors associated with risk of burnout among healthcare workers, especially for EE. In systematic reviews, violence was identified as a potential risk factor for an increased risk of burnout [2, 17–21]. However, only few studies cover the context of German nurses, with one of the largest representation found in a study on European nurses published in 2008 [21].

Although the mechanism behind the association remains underexplored, repeated exposure to violence has been shown to affect EE and the risk of burnout through chronic stress [16, 22, 23] and fear of future violence [24]. In healthcare, workplace violence (WV) is defined as “any act of verbal or physical violence or obstruction or threat of violence that interferes with the availability, access and delivery of curative and/or preventive health services during emergencies.” [25]. The World Health Organization (WHO) has designated WV a global issue [26] emphasizing its recurrence both historically and throughout the COVID-19 pandemic [27]. Meta-analyses confirm high exposure: the prevalence of verbal abuse against nurses is reported at 77% (CI: 72–82%), while general physical violence is reported at 20% (CI: 16%-24%) [28]. Prevalences of 35% (CI: 29–42%) are reported in cases involving physical client-perpetrated violence directed at US nurses [29]. Additionally, 12% (CI: 7–17%) report experiences of sexual harassment in south-east Asian and western pacific nurses [30].

Nurses regularly operate in dynamic and highly challenging environments, rendering them disproportionally vulnerable to overwhelming stressors. Understanding nurses’ responses to stressors such as violence is crucial, but German nurses remain underrepresented in the literature. A 2021 systematic review on health outcomes and experiences of violence among German nurses identified only nine studies addressing this topic [31]. These studies show high frequencies of violence, with 52% experiencing verbal or physical violence at least once a week [32], and 12-month prevalence rates of up to 94.1% [33, 34]. Burnout is widely reported with over 65% of nurses exhibiting moderate to high levels of risk of burnout in the EE dimension [35, 36]. However, only a few studies assessed the relationship between violence and burnout or EE: A 2020 study involving 582 German nurses reports a positive association between violence exposure and emotional exhaustion in regression analyses [37]. In contrast, a 2022 study of 349 emergency department workers in Germany using the Copenhagen burnout scale found no significant association between frequency of self-reported violence and burnout in multivariate models [38]. Consequently, studies on violence exposure of German nurses and the associated health risks, such as burnout, are scarce, and findings from international studies are constrained by differences in health systems [31]. Addressing this gap is essential to improve occupational health and working conditions for nurses in Germany [31, 39]. Recent publications suggest growing interest in this area, exemplified by a 2020 study that explored the moderating role of follow-up counseling on burnout dimensions. The study found that the availability of follow-up counselling moderated the relationship between verbal violence and depersonalization but not emotional exhaustion [37].

This study aims to analyze the association between the frequency of experienced WV and the risk of burnout for the EE dimension in German registered nurses (RNs), adjusting for covariables. Bayesian regression methods that build on prior findings in similar research are applied, allowing parameter estimates to be re-used in future Bayesian research.

## Methods

### Data source

Data were obtained from RNs as part of the German 2015 follow-up study to the Registered Nurse Forecasting (RN4Cast) project, the so called “G-NWI-Study”. The project initially aimed at identifying and predicting nurse workforce needs and developments [40]. This follow-up employed a cross-sectional design and used a paper-based, anonymous survey administered to RNs working in acute care hospitals. Participating hospitals included those previously involved in the RN4Cast study (2009–2011), as well as additional acute care hospitals with at least 100 beds. Hospitals specializing in rehabilitation, pediatrics, or psychiatry were excluded from participation. In total, 71 hospitals took part, and 4317 RNs returned at least partially completed surveys with informed consent [41]. Prepaid envelopes were provided for returning both the completed questionnaire and consent form to Berlin University of Technology. Survey forms were mechanically read into a digital dataset.

### Variable selection, instruments and measures

To identify relevant independent adjustment covariables that model MBI-burnout and EE, a comprehensive literature search was conducted. The selected variables included employment type, work, and wage satisfaction as well as work environment aspects. Demographic variables and violence frequency were also included.

As violence was the primary exposure of interest, and no validated instrument was present in the dataset, another literature search was conducted to determine suitability of the measures of self-reported violence. More details on both literature searches can be found in Appendix B. Kendall’s $\:\tau\:$ was used to check for collinearity among independent variables. The age category was excluded in favor of years of seniority, as these were collinear in the sample ($\:\tau\:=0.78\:$). Seniority was retained, as it more accurately reflects the time spent in a potentially demanding work environment.

The survey included a section on demographics and employment characteristics, capturing information on training and job-related academic qualifications. The Practice Environment Scale of the Nursing Work Index (PES-NWI, hereafter PES), a validated instrument, was used to assess key elements of the nurse working environment. It consists of 32 Likert-scale items (1: “Disagree” to 4: “Agree”) covering five domains: nurse participation in hospital affairs, nursing foundation for quality of care, nurse manager ability, leadership and support for RNs, staffing and resource adequacy and collegial nurse-physician relations. Scoring is heterogeneous throughout the literature [42]. In this analysis, subscales were dichotomized into favorable (positive, mean > 2.5) or unfavorable (negative) working environments [43]. The research edition was translated by the Technische Universität Berlin in 2009 and revised in 2014. Furthermore, the questionnaire included items on perceived patient safety, quality of care, satisfaction with working conditions, staffing, task distribution and missed care activities within the respondents’ units. More details about the study and project are described elsewhere [40].

Burnout risk was measured using the Maslach Burnout Inventory – Human Services Survey (MBI-HSS), which was translated and validated for Germany [44]. The MBI is one of the most commonly used instruments to measure burnout risk [2, 7]. It consists of 22 items rated on a seven-point Likert scale (0: “Never”; 1: “A few times a year or less”; 2: “Once a month or less”; 3: “A few times a month”; 4: “Once a week”; 5: “A few times a week”; 6: “Every day”). It maps three dimensions of burnout: emotional exhaustion (nine items), depersonalization (five items) and personal accomplishment (eight items) [45]. Of these, EE is the primary dimension of burnout related to stress and job demands [7–9]. Therefore, this analysis focused on EE as the central dimension of burnout, capturing stress, depletion, and overextension of emotional resources towards workers’ ability to commit to their work [46]. Depersonalization (DP) captures detached or cynical attitudes response towards recipients of care, while personal accomplishment (PA) entails feelings of reduced productivity and competence [47]. Risk on each dimensions were categorized as “low”, “middle”, or “high” risk using established cutoffs, despite ongoing debate and inconsistency in their application [48]. This analysis categorizes risk of “High EE” as sum scores $\:\ge\:$ 27, “Moderate EE” as values $\:<$ 27 and $\:\ge\:$ 19, and “Low EE” as values $\:<$ 18 (score range: 0–54), with higher scores indicating higher risk. The MBI was originally developed as a research tool, although often used as a diagnostic instrument in research and medicine [48]. In this work, emphasis is put on the MBI as a research tool measuring risk in each of the three dimensions, rather than a diagnosis. Cronbach’s standardized alpha suggested high internal consistency ($\:{\alpha\:}_{std}=0.88$) [49].

The survey also included four items assessing the frequency of physical and verbal violence experienced over the last 12 months. Participants were asked how often they experienced physical or verbal violence, (e.g. “How often would you say each of the following incidents occurs involving you: Physical violence”) either from patients and/or family members (WV Physical 1; WV Verbal 1) or from staff members (WV Physical 2; WV Verbal 2) using a seven-point Likert scale (0: “Never”; 1: “Once or less in a year”; 2: “Once or less in a month”; 3: “Multiple times a month”; 4: “Once a week”; 5: “Multiple times a week”; 6: ”Daily”). The measurement did not adjust for violence severity. For analysis, WV frequencies were aggregated into three groups: “Low”: responses 0–1 (never or once a year), “Moderate”: responses 2–3 (monthly), and “High”: responses 4–6 (weekly or more frequently). An exception was made for physical violence by staff, which was dichotomized into “Lower” (responses $\:<$3) or “Higher” (responses  $\:\ge\:$3) , due to low group size. Wage and work satisfaction, measured with four-point Likert scales, was dichotomized into “Low” or “High” via midpoint split. A sum score of nine missed care activities from the questionnaire was created in the auxiliary variables to economize imputation. All data transformations were performed before modelling.

### Statistical methods

Missing data were addressed using multiple imputed chained equations (MICE) with fully conditional specification (FCS) within the mice package [50]. Missingness on multi-item composite scores (EE; PES-subdimensions) was addressed using passive imputation, which yield more stable estimates and exploit differential missingness between the subscales [51, 52]. To increase information for MI, additional variables were selected from a pool of auxiliary variables. Prediction matrices of selected variables are reported in Appendix A1. A total of 33 imputed datasets were generated. The convergence of imputations was monitored using potential scale reduction factor $\:\widehat{R}$ (PSRF) and visual inspections of chain means and standard deviations (more details are found in Appendix C1).

Descriptive results were expressed using percentages and means. To analyze the association between WV frequency and the risk of EE, a Bayesian proportional continuation ratio model with a logistic link was fit using the brms package [53]. This model type was chosen because of the natural compatibility with MI procedures and the possibility to include information from prior studies and was adjusted by other covariables. Priors were elicited by calculating weighted averages of relevant regression weights from the literature (Sect. “Variable selection, instruments and measures”).

Three prior sets (set 1, 2 and 3), expressing different levels of prior information confidence, were used for sensitivity analyses to ensure robustness. Prior set 1 represented mildly informative normal priors and was used for main analysis. All priors are formalized in Appendix A2. Suitability was assessed using prior and posterior predictive checks. For significance, 90% posterior equal-tailed credible intervals were estimated. As a sensitivity analysis of the imputation procedure, the model was additionally fit to CC data. Chain convergence and resolution were assessed using PSRF $\:\widehat{R}$ and effective sample size measures. For both MI and Bayesian modelling, random seeds were chosen, and results compared for robustness. Technical details of the Bayesian model are described in Appendix C2. All analyses were conducted using RStudio (version 4.3.3) and packages from RStudio (mice, brms). To enhance transparency, (reporting) guidelines for Bayesian analysis and multiple imputation methods [54, 55], as well as STROBE-guidelines for cross-sectional designs were applied [56].

## Results

A total of 4317 responses were recorded during the study period. After deleting one entry in self-rated quality of care due to a coding error, a CC sample of *n* = 2897 (67.1%) was available for sensitivity analysis. A full appraisal of missing data items is found in Appendix C3.

### Descriptive results of model variables

Table 1 shows descriptive results of variables included in the models. CC results are reported as sensitivity analysis to provide insights into the imputation model’s allocation of missing values and to assess deviations from original data distributions.

A high share of RNs was at an increased risk of burnout, as measured by the MBI for the EE dimension. According to MI estimates, 26.8% of respondents were at moderate risk and 37% were at high risk of EE. Most participants were female (84.8%) and working full-time (64.3%). A minority held an academic nursing degree (2.1%). The most frequently reported high exposure to WV was verbal violence by patients or family members. In contrast, physical violence was less frequent: 3.8% of RNs reported high frequency of physical violence from patients and family members, while 10% reported higher levels of physical violence perpetrated by staff.

**Table 1:** Description of CC and MI data of RN

Variables	MI data, *n* = 4317	CC data, *n* = 2897
EE	Low: 36.0%; Moderate: 26.9%; High: 37.1%	Low: 36.2%; Moderate: 26.8%; High: 37.0%
Sex	Male: 14.0%; Female: 86.0%	Male: 15.2%; Female: 84.8%
Seniority [mean (sd)]	17.1 (11.9)	16.8 (11.7)
Employment	Part-time: 36.6%; Full-time: 63.4%	Part-time: 35.7%; Full-time: 64.3%
Training	Academic nursing degree: 2.3%	Academic nursing degree: 2.1%
PES Participation	Positive: 56.4%	Positive. 55.7%
PES Quality foundations	Positive: 53.2%	Positive: 52.5%
PES Staffing adequacy	Positive: 13.7%	Positive: 13.9%
PES Manager ability	Positive: 67.4%	Positive: 67%
PES Physician relations	Positive: 59.5%	Positive: 58.3%
Work Satisfaction	Positive: 54.9%	Positive: 54.2%
Wage Satisfaction	Positive: 30.9%	Positive: 30.7%
WV Verbal 1	Low: 29.2%; Moderate: 52.2%; High: 21.6%	Low: 25.3%; Moderate: 52.2%; High: 22.4%
WV Verbal 2	Low: 60%; Moderate: 32.2%; High: 7.8%	Low: 58%; Moderate: 33.4%; High: 8.6%
WV Physical 1	Low: 69.5%; Moderate: 26.7%; High: 3.8%	Low: 68.5%; Moderate: 27.6%; High: 3.8%
WV Physical 2	Lower: 90.3%; Higher: 9.7%	Lower: 90%; Higher: 10%

Note: Training data was manually imputed using free text entries (see appendix C3); CC: Complete cases; MI: Multiple imputations; WV Verbal 1: Verbal workplace violence by patients and family members; WV Verbal 2: Verbal workplace violence by staff; WV Physical 1: Physical workplace violence by patients and family members; WV Physical 2: Physical workspace violence by staff; EE: Emotional exhaustion

Aggregated statistics showed that MI datasets largely mimicked the original data in both populations. Convergence was achieved without the need for further adjustments. The results generated from different seeds did not differ significantly. Results of the convergence measures are shown in Appendix A3.

### Results of Bayesian regression models

Posterior means (regression coefficient estimates) and 90% credible intervals (CI) from the MI analysis with prior set 1 are reported in Table 2. CC results are reported as sensitivity analysis.

**Table 2:** Regression results for EE risk categories

Coefficient – RNs data	Posterior mean - MI (OR)	90% Credible intervals - MI (L, *R*)	Posterior mean - CC (OR)	90% Credible intervals - CC (L, *R*)
**Sex: Female**	**0.16 (1.17)**	**(1.04, 1.31)**	**0.19 (1.21)**	**(1.06, 1.37)**
**Seniority**	**0.01 (1.01)**	**(1.005, 1.013)**	**0.01 (1.01)**	**(1.004, 1.014)**
**Employment: Fulltime**	**0.41 (1.5)**	**(1.37**,** 1.66)**	**0.45 (1.57)**	**(1.39**,** 1.76)**
Academic degree	-0.06 (0.94)	(0.78, 1.15)	-0.08 (0.92)	(0.75, 1.12)
**Wage Satisfaction**	**-0.48 (0.62)**	**(0.57**,** 0.68)**	**-0.47 (0.63)**	**(0.56**,** 0.69)**
PES Participation	-0.11 (0.89)	(0.79, 1.002)	-0.07 (0.93)	(0.81, 1.07)
**PES Quality foundations**	**-0.25 (0.78)**	**(0.7**,** 0.87)**	**-0.29 (0.75)**	**(0.65**,** 0.85)**
**PES Staffing adequacy**	**-0.57 (0.57)**	**(0.49**,** 0.64)**	**-0.59 (0.55)**	**(0.47**,** 0.65)**
PES Manager ability	-0.1 (0.9)	(0.81, 1.008)	**-0.2 (0.82)**	**(0.72**,** 0.93)**
PES Physician relations	-0.03 (0.97)	(0.88, 1.08)	-0.05 (0.95)	(0.84, 1.07)
**Satisfaction**	**-1.1 (0.33)**	**(0.3**,** 0.37)**	**-1.07 (0.34)**	**(0.3**,** 0.39)**
**WV Verbal 1: Moderate**	**0.24 (1.27)**	**(1.15**,** 1.41)**	**0.28 (1.32)**	**(1.17**,** 1.5)**
**WV Verbal 1: High**	**0.57 (1.77)**	**(1.54**,** 2.02)**	**0.5 (1.65)**	**(1.4**,** 1.92)**
WV Verbal 2: Moderate	0.09 (1.09)	(0.99, 1.2)	0.11 (1.12)	(0.99, 1.25)
**WV Verbal 2: High**	**0.34 (1.4)**	**(1.17**,** 1.67)**	**0.33 (1.39)**	**(1.15**,** 1.69)**
**WV Physical 1: Moderate**	**0.2 (1.22)**	**(1.1**,** 1.36)**	**0.29 (1.34)**	**(1.19**,** 1.5)**
**WV Physical 1: High**	**0.36 (1.43)**	**(1.13**,** 1.8)**	**0.51 (1.66)**	**(1.29**,** 2.17)**
**WV Physical 2: High**	**0.22 (1.24)**	**(1.08**,** 1.43)**	**0.17 (1.18)**	**(1.002**,** 1.4)**

Note: OR: Odds ratio; WV Verbal 1: Verbal workplace violence by patients and family members; WV Verbal 2: Verbal workplace violence by staff; WV Physical 1: Physical workplace violence by patients and family members; WV Physical 2: Physical workspace violence by staff; EE: Emotional exhaustion; PES: Practice environment scale; CC: Complete cases; MI: Multiple imputations; Posterior means represent the regression coefficient estimates$\:\left({\upbeta\:}\right)$; 90% left (L) and right (R) posterior equal-tailed intervals are reported; Results in bold are significant to the 90% credible interval

All WV variables had an effect within a 90% credible interval. Compared to low frequency exposure, a high frequency of verbal violence from patients or family members increased the risk of transitioning into a higher EE risk category by 77% for RNs (OR: 1.77; CI: 1.54–2.02). Similarly, high frequency of verbal violence from staff increased risk by 40% (OR: 1.4; CI: 1.17–1.67) compared to a low frequency. A high frequency of physical violence by patients or family members exhibited a similar effect size, increasing the risk of higher risk of EE by 43% compared the reference (OR: 1.42; CI: 1.13–1.8). Posterior means revealed an exposure pattern: higher frequencies of WV had a stronger effect on risk of increased EE than moderate frequencies. However, moderate frequency exposure of verbal violence by staff did not yield a significant estimate. The strongest association was observed for the frequency of verbal violence by patients and family members. The results of violence frequency were similar between MI and the sensitivity analysis using CC data. Except for PES manager ability dimension (“Manager ability, leadership & support of nurses”), this was also true for the other covariates. Job satisfaction emerged as the strongest protective factor with risk of EE, reducing increased EE risk by 67% with high posterior confidence (OR: 0.33; CI: 0.3–0.37).

### Diagnostics of Bayesian regression models

Posterior and prior predictive plots indicated a best fit for the data of prior sets 1 and 2, which suggests that the priors did not introduce bias. Prior checks are found in Appendix A4. Posterior checks can be found in Appendix A5. For MI, predictive checks were conducted for each of the 33 imputed datasets, although detailed outputs are not reported here. Appendix A6 shows posterior density comparisons with different prior sets as sensitivity checks on the estimates of MI data. Distributions largely agree between the sets, suggesting that the data can overwhelm priors. Since MI analysis requires convergence of each chain in all imputed datasets, potential signs of non-convergence were inspected via summary statistics but not fully reported. None of the parameters showed signs of non-convergence and effective sample size was sufficient across parameters. Proof of convergence for the CC model with prior set 1 can be found in Appendix A7.

## Discussion

According to the present estimates, a high proportion (37.1%) of RNs were at high risk of EE, while 26.9% were at moderate risk. Comparisons with other studies are limited by the lack of comparable German studies using similarly sizes samples and equivalent measurement tools. Nevertheless, our estimate is lower than that reported in a meta-analysis of physicians and emergency department nurses using the same MBI version and EE cutoffs, which found a prevalence of 48% (CI: 33–63%) [15]. This may be attributable to the inclusion of intensive care unit and emergency department workers, as well as physicians, although nurses constituted the majority of the sample. By contrast, a lower prevalence of 25% was reported for EE in mental health nurses (CI: 17–35%) [16]. Another German study from 2010 using EE mean scores on the MBI-General survey reported only 15.1% of nurses being at high risk of burnout, however this study combined moderate and high risk (65%) for regression analyses [35].When applying the same grouping, the current estimate for EE of our study (63.8%) is comparable.

The most reported form of frequent violence in the sample was verbal violence by patients or family members (21.6%), while physical violence by the same perpetrators was less frequent (3.8%). The high frequency of verbal violence is close to overall high violence frequency by patients or family members in an older study on European nurses (22%) [21], and similar to an estimate of the 12-month prevalence of verbal violence by patients and relatives in a German analysis when moderate and high categories are grouped (73.8% vs. 77.8%). Yet in the same comparison, physical violence is less frequently reported in the present sample (30.5% vs. 56.2%) [34].

Other population characteristics were consistent with previous research. The proportion of female respondents (86.0%) is comparable to other German studies (80%-90%) [35, 37]. These studies report that the highest category of job seniority was over 15 years with over 62% of responses [37] or 3–12 years (38.9%) and > 12 years (37.9%) [35], while the present sample had an average seniority of 17.1 years with a wide standard deviation. In total, 36.6% of the surveyed nurses reported working part-time, comparable to the landscape at the time of the survey [57] and lower compared to a more recent analysis of German nurses with 57% [58].

In line with the study’s aims, our models estimate that frequency of WV is associated with risk of higher EE in German nurses, with moderate to high posterior confidence. Associations were robust across verbal and physical violence, regardless of perpetrator group, and consistent across sensitivity analyses using CC data and different priors. Although work environment and demographic factors have been examined extensively elsewhere, these findings underscore WV as a critical occupational stressor for German nurses.

Mitigating WV remains challenging due to the unpredictability of violent events. Current interventions focus largely on individual-level strategies (e.g., de-escalation training and communication) [59, 60] and organizational measures (e.g., joint staff decisions, feedback, support and safety culture) [60–63]. Enhanced reporting and accountability may also deter violence [62], but this presupposes that incidents are both reported and addressed effectively. A combination of these interventions appears most promising, but studies lack quality and the impact of singular personal and organizational interventions are described as limited [64, 65]. Similarly, burnout prevention interventions often focus on personal strategies. Systematic reviews point towards practices of mental health and physical wellbeing as interventions with mixed effectiveness and study quality [66, 67]. While this suggests that more research is needed for both WV and burnout prevention, such approaches may also inadvertently shift the burden to individual staff rather than addressing structural stressors. Recognition of burnout as a work-related illness by German professional associations could help mobilize resources and increase public awareness and scientific attention to develop effective prevention strategies.

## Limitations

The primary limitation is the age of the dataset, which may not accurately reflect current conditions in nursing, including WV, burnout and other occupational outcomes. Evidence suggests that burnout among nurses is increasing globally [14], and WV also appears to be on the rise [29, 68]. Evidence on trends in violence against healthcare workers in Germany remains scarce, yet recent policy developments point to a growing readiness to use violence against them [69]. The age of this sample may restrict generalizability, but recent studies on the relationship between WV and burnout or EE suggests that the direction and significance of association remains in the present workforce [17, 18]. Furthermore, the survey did not include psychiatric, rehabilitation or pediatric nurses and was limited to certain hospitals participating in the RN4Cast follow up study.

Another limitation concerns self-reported measures, including the lack of validated WV instruments. Nevertheless, the analysis of WV frequency variables was comparable to other analyses applied to the German context [21, 32] and internationally [70–72]. While the categories of low, moderate and high frequency were arbitrary, studies using similar frequency items used “monthly or more” as a cutoff for increased exposure [21, 73, 74]. Other studies delineated frequency of violence in terms of yearly, monthly or weekly occurrences [75]. One analysis reports that nurses were exposed to physical and emotional violence once a month on average [76], grounding the distinction between the categories created to a non-normative frequency. However, these measures do not adjust for the severity of violence. For future research, validated instruments should be used. Another limitation in this and other works is the lack of self-reported sexual violence. Men and women experience sexual violence, but violent experiences by women, who are disproportionally targeted, are possibly underrepresented [77]. It should be noted that in certain care settings, patient-related violence (e.g., from intoxicated, demented or delirious patients) might be perceived as part of the job. Such perceptions could influence how workplace violence is assessed and reported, and how the resulting stress and job demands are perceived, which represents a potential limitation of our study.

RNs took their survey at work, which can introduce availability bias [78]. Moreover, surveys at work may not capture responses from those affected the most by both WV and burnout. This can introduce a sort of survivor bias, which is also theorized to affect younger workers [35, 46] and may also play a role in the reporting of WV due to stigma or shame [79, 80]. The use of MBI-measured EE risk is in line with the literature. While some studies use a composite of all three dimensions, this is not recommended by MBI’s authors [2]. Still, this comes with limitations: As elaborated in a systematic review on the usage of cutoffs for the MBI dimensions, there is ambiguity in classification, but most studies use a cutoff of 27 for risk of ”high EE” [48]. Furthermore, EE is associated with further psychological burden, such as depression [81], which could be a competing occupational outcome and is itself associated with EE [82]. For example, DP constitutes a burnout dimension which is less associated with depression [81, 82], implying that further research is needed to discern distinctive effects on burnout dimensions. Variable selection was based on a literature search. This search did not satisfy the requirements of a systematic review and not every identified domain was available in the data. Notably, we lacked some of the classical working conditions variables such as autonomy and time pressure, using the PES-dimensions as proxies for adjustment. Highly sensitive items were imputed. Selecting from a large pool of auxiliary variables using different strategies is prone to a multiple testing problem. However, imputation models do not need to be conceptually meaningful [83]. The statistical model was constrained by the proportional-odds-assumption and did not include interaction terms. While nonproportional models provide more refined effects, they are rarely useful, imply severe restrictions on the model parameter and complicate interpretation [84]. Lastly, the cross-sectional design and models do not allow for causal inference and ignore the possibility of different associative scenarios, such as risk of EE causing violence and conflict more frequently, resulting in a feedback loop between mental health and violence.

## Conclusion

This work contributes to the body of evidence on the association between WV and the risk of the EE dimension of burnout in German nurses. It applies a unified MI and Bayesian regression approach to analyze complex survey data. Building on the information gathered from literature, the data and models reinforce WV as a significant workplace stressor associated with risk of EE. This subsequently disrupts workforce development and adversely affects the quality of care delivered to patients. Therefore, violence prevention must be recognized as a shared concern for both healthcare workers and patients. Health care workers can perceive WV from patients as a natural “part of the job” and work demand in certain health care settings, such as working with patients in a heightened state of arousal or disorientation. However, a normalization of violent experiences may conflict with approaches to prevent the causes of WV as well as its effects, especially when it arises from other perpetrators. This dilemma may serve as a natural frame of reference for future policy positions and interventions. There is an urgent need for evidence-supported interventions to prevent and mitigate the effects of WV, backed by well-designed studies. WV incidents are largely unpredictable and research on violence prevention remains inconclusive. Recognizing burnout as a work-related disease could incentivize research, fostering the development of effective prevention and treatment strategies. The results of this work can be re-used for future (Bayesian) research, updating the evidence and providing more robust insights.

## Supplementary Information

Below is the link to the electronic supplementary material.

Supplementary Material 1

Supplementary Material 2

Supplementary Material 3

## Data Availability

The dataset analyzed in this study is not publicly available due to the sensitive nature of the data but is available from the corresponding author on reasonable request. The appendices include extensive descriptions of designed and deployed methods and materials used.

## Abbreviations

RNs: Registered Nurses
WHO: World Health Organization
WV: Workplace Violence
COVID 19: Coronavirus–Disease 2019
ICD-11: International Classification of Diseases, 11th version
RN4Cast: Registered Nurse Forecasting
PES-NWI (PES): Practice Environment Scale of the Nursing Work Index
MBI-HSS/MBI: Maslach Burnout Inventory–Human Services Survey
EE: Emotional Exhaustion
DP: Depersonalization
STROBE: Strengthening the Reporting of Observational Studies in Epidemiology
WV Verbal 1: Verbal workplace violence from patients or family members
WV Verbal 2: Verbal workplace violence from staff
WV Physical 1: Physical workplace violence from patients or family members
WV Physical 2: Physical workplace violence from staff
CC: Complete Cases
MI: Multiple Imputation
CI: Credible Interval
OR: Odds Ratio

## References

[1] Gómez-Urquiza JL, La Fuente-Solana, Albendín-García EI, Vargas-Pecino C, Ortega-Campos EM. La Cañadas-De Fuente GA. Prevalence of burnout syndrome in emergency nurses: a meta-analysis. Crit Care Nurse. 2017;37:e1–9. 10.4037/ccn2017508. 28966203 10.4037/ccn2017508

[2] Dall’Ora C, Ball J, Reinius M, Griffiths P. Burnout in nursing: a theoretical review. Hum Resour Health. 2020;18:41. 10.1186/s12960-020-00469-9. 32503559 10.1186/s12960-020-00469-9 PMC7273381

[3] Freudenberger HJ. Staff Burn-Out. J Soc Issues. 1974;30:159–65. 10.1111/j.1540-4560.1974.tb00706.x.

[4] Maslach C, Schaufeli WB. In: Schaufeli WB, Maslach C, Maslach C, editors. Historical and conceptual development of burnout. Professional Burnout: Routledge; 2017. pp. 1–16. 10.4324/9781315227979-1.

[5] Schaufeli WB, Leiter MP, Maslach C. Burnout: 35 years of research and practice. Career Dev Int. 2009;14:204–20. 10.1108/13620430910966406.

[6] WHO. Burn-out an. Occupational Phenomenon. 2025. https://www.who.int/standards/classifications/frequently-asked-questions/burn-out-an-occupational-phenomenon. Accessed 30 May 2024.

[7] Schaufeli WB, Desart S, de Witte H. Burnout assessment tool (BAT)-Development, validity, and reliability. Int J Environ Res Public Health. 2020. 10.3390/ijerph17249495. 33352940 10.3390/ijerph17249495 PMC7766078

[8] Demerouti E. Burnout: a comprehensive review. Z Arb Wiss. 2024;78:492–504. 10.1007/s41449-024-00452-3.

[9] van Ruysseveldt J, Verboon P, Smulders P. Job resources and emotional exhaustion: the mediating role of learning opportunities. Work Stress. 2011;25:205–23. 10.1080/02678373.2011.613223.

[10] Wells A. Stress. In: Freeman A, Dattilio FM, editors. Comprehensive casebook of cognitive therapy. Boston, MA: Springer US; 1992. pp. 99–106. 10.1007/978-1-4757-9777-0_9.

[11] Folkman S, Stress. Appraisal and coping. In: Gellman MD, Turner JR, editors. Encyclopedia of behavioral medicine. New York, NY: Springer New York; 2013. pp. 1913–5. 10.1007/978-1-4419-1005-9_215.

[12] Winwood PC, Lushington K. Disentangling the effects of psychological and physical work demands on sleep, recovery and maladaptive chronic stress outcomes within a large sample of Australian nurses. J Adv Nurs. 2006;56:679–89. 10.1111/j.1365-2648.2006.04055.x. 17118046 10.1111/j.1365-2648.2006.04055.x

[13] McEwen BS. Physiology and neurobiology of stress and adaptation: central role of the brain. Physiol Rev. 2007;87:873–904. 10.1152/physrev.00041.2006. 17615391 10.1152/physrev.00041.2006

[14] Ge M-W, Hu F-H, Jia Y-J, Tang W, Zhang W-Q, Chen H-L. Global prevalence of nursing burnout syndrome and Temporal trends for the last 10 years: A meta-analysis of 94 studies covering over 30 countries. J Clin Nurs. 2023;32:5836–54. 10.1111/jocn.16708. 37194138 10.1111/jocn.16708

[15] Lin K-H, Selvanayagam N, Patnaik S, Kuo C-Y. Burnout among physicians and nurses working in intensive care units and emergency departments: a systematic review and meta-analysis. J Emerg Nurs. 2025. 10.1016/j.jen.2025.02.007. 40088246 10.1016/j.jen.2025.02.007

[16] López-López IM, Gómez-Urquiza JL, Cañadas GR, La Fuente EI, Albendín-García L, La Cañadas-De Fuente GA. Prevalence of burnout in mental health nurses and related factors: a systematic review and meta-analysis. Int J Ment Health Nurs. 2019;28:1032–41. 10.1111/inm.12606. 31132216 10.1111/inm.12606

[17] Amiri S, Mahmood N, Mustafa H, Javaid SF, Khan MA. Occupational risk factors for burnout syndrome among healthcare professionals: a global systematic review and meta-analysis. Int J Environ Res Public Health. 2024. 10.3390/ijerph21121583. 39767426 10.3390/ijerph21121583 PMC11675210

[18] Berger S, Grzonka P, Frei AI, Hunziker S, Baumann SM, Amacher SA, et al. Violence against healthcare professionals in intensive care units: a systematic review and meta-analysis of frequency, risk factors, interventions, and preventive measures. Crit Care. 2024;28:61. 10.1186/s13054-024-04844-z. 38409034 10.1186/s13054-024-04844-z PMC10898135

[19] Sahebi A, Golitaleb M, Moayedi S, Torres M, Sheikhbardsiri H. Prevalence of workplace violence against health care workers in hospital and pre-hospital settings: an umbrella review of meta-analyses. Front Public Health. 2022. 10.3389/fpubh.2022.895818. 36003634 10.3389/fpubh.2022.895818 PMC9393420

[20] Lanctôt N, Guay S. The aftermath of workplace violence among healthcare workers: A systematic literature review of the consequences. Aggress Violent Beh. 2014;19:492–501. 10.1016/j.avb.2014.07.010.

[21] Estryn-Behar M, van der Heijden B, Camerino D, Fry C, Le Nezet O, Conway PM, Hasselhorn H-M. Violence risks in nursing–results from the European ‘NEXT’ study. Occup Med (Lond). 2008;58:107–14. 10.1093/occmed/kqm142. 18211910 10.1093/occmed/kqm142

[22] Lu L, Dong M, Wang S-B, Zhang L, Ng CH, Ungvari GS, et al. Prevalence of workplace violence against health-care professionals in China: a comprehensive meta-analysis of observational surveys. Trauma Violence Abuse. 2020;21:498–509. 10.1177/1524838018774429. 29806556 10.1177/1524838018774429

[23] Murray RM, Davis AL, Shepler LJ, Moore-Merrell L, Troup WJ, Allen JA, Taylor JA. A systematic review of workplace violence against emergency medical services responders. New Solut. 2020;29:487–503. 10.1177/1048291119893388. 31841060 10.1177/1048291119893388 PMC8594050

[24] Fu C, Wang G, Shi X, Ren Y, Cao F. The association between fear of future workplace violence and burnout among nurses in China: a cross-sectional study. J Affect Disord. 2021;293:29–35. 10.1016/j.jad.2021.06.013. 34166906 10.1016/j.jad.2021.06.013

[25] WHO. Stopping attacks on health care. 2023. https://www.who.int/activities/stopping-attacks-on-health-care. Accessed 30 May 2024.

[26] ILO. Framework guidelines for addressing workplace violence in the health sector. 2 January 2002. https://iris.who.int/bitstream/handle/10665/42617/9221134466.pdf?sequence=1

[27] ILO. Violence against healthcare: current practices to prevent, reduce or mitigate violence against health care. July 2022. https://www.wma.net/wp-content/uploads/2022/07/Violence-against-healthcare-survey-report.pdf

[28] Aljohani B, Burkholder J, Tran QK, Chen C, Beisenova K, Pourmand A. Workplace violence in the emergency department: a systematic review and meta-analysis. Public Health. 2021;196:186–97. 10.1016/j.puhe.2021.02.009. 34246105 10.1016/j.puhe.2021.02.009

[29] McLaughlin L, Khemthong U. The prevalence of type II workplace violence in US nurses 2000 to 2022: A Meta-Analysis. West J Nurs Res. 2024;46:248–55. 10.1177/01939459231222449. 38229584 10.1177/01939459231222449

[30] Varghese A, Joseph J, Vijay VR, Khakha DC, Dhandapani M, Gigini G, Kaimal R. Prevalence and determinants of workplace violence among nurses in the South-East Asian and Western Pacific regions: a systematic review and meta-analysis. J Clin Nurs. 2022;31:798–819. 10.1111/jocn.15987. 34351652 10.1111/jocn.15987

[31] Schaller A, Klas T, Gernert M, Steinbeißer K. Health problems and violence experiences of nurses working in acute care hospitals, long-term care facilities, and home-based long-term care in germany: a systematic review. PLoS ONE. 2021;16:e0260050. 10.1371/journal.pone.0260050. 34793537 10.1371/journal.pone.0260050 PMC8601565

[32] Franz S, Zeh A, Schablon A, Kuhnert S, Nienhaus A. Aggression and violence against health care workers in Germany–a cross sectional retrospective survey. BMC Health Serv Res. 2010;10:51. 10.1186/1472-6963-10-51. 20184718 10.1186/1472-6963-10-51 PMC2837654

[33] Schablon A, Wendeler D, Kozak A, Nienhaus A, Steinke S. Prevalence and consequences of aggression and violence towards nursing and care staff in Germany-A survey. Int J Environ Res Public Health. 2018 10.3390/ijerph15061274. 10.3390/ijerph15061274 PMC6025092 29914142

[34] Schablon A, Zeh A, Wendeler D, Peters C, Wohlert C, Harling M, Nienhaus A. Frequency and consequences of violence and aggression towards employees in the German healthcare and welfare system: a cross-sectional study. BMJ Open. 2012. 10.1136/bmjopen-2012-001420. 23087013 10.1136/bmjopen-2012-001420 PMC3488706

[35] Kowalski C, Ommen O, Driller E, Ernstmann N, Wirtz MA, Köhler T, Pfaff H. Burnout in nurses - the relationship between social capital in hospitals and emotional exhaustion. J Clin Nurs. 2010;19:1654–63. 10.1111/j.1365-2702.2009.02989.x. 20384668 10.1111/j.1365-2702.2009.02989.x

[36] Vaupel C, Vincent-Höper S, Helms L, Adler M, Schablon A. Sexuelle Belästigung und Gewalt in Pflege- und Betreuungsberufen: - Ergebnisbericht für die Branche Krankenhäuser - Bereiche Psychiatrie, Allgemeinkrankenhäuser und Rehabilitationskliniken. November 2020. https://www.bgw-online.de/resource/blob/22158/40b558f7249c9c76419103469a5c7b29/bericht-gewalt-krhs-data.pdf

[37] Vincent-Höper S, Stein M, Nienhaus A, Schablon A. Workplace aggression and burnout in Nursing-The moderating role of follow-up counseling. Int J Environ Res Public Health. 2020. 10.3390/ijerph17093152. 32369903 10.3390/ijerph17093152 PMC7246829

[38] Schablon A, Kersten JF, Nienhaus A, Kottkamp HW, Schnieder W, Ullrich G, et al. Risk of burnout among emergency department staff as a result of violence and aggression from patients and their relatives. Int J Environ Res Public Health. 2022. 10.3390/ijerph19094945. 35564338 10.3390/ijerph19094945 PMC9105776

[39] Schaller A, Gernert M, Klas T, Lange M. Workplace health promotion interventions for nurses in germany: a systematic review based on the RE-AIM framework. BMC Nurs. 2022;21:65. 10.1186/s12912-022-00842-0. 35313866 10.1186/s12912-022-00842-0 PMC8935842

[40] Sermeus W, Aiken LH, van den Heede K, Rafferty AM, Griffiths P, Moreno-Casbas MT, et al. Nurse forecasting in Europe (RN4CAST): rationale, design and methodology. BMC Nurs. 2011;10:6. 10.1186/1472-6955-10-6. 21501487 10.1186/1472-6955-10-6 PMC3108324

[41] Köppen J, Zander B, Busse R. Die aktuelle Situation der stationären Krankenpflege in Deutschland. 2017. https://www.gesundheitskongresse.de/berlin/2017/dokumente/praesentationen/Koeppen-Julia---Nurse-Forecasting-zur-quantitativen-und-qualitativen-Bedarfsplanung.pdf

[42] Swiger PA, Patrician PA, Miltner RSS, Raju D, Breckenridge-Sproat S, Loan LA. The practice environment scale of the nursing work index: an updated review and recommendations for use. Int J Nurs Stud. 2017;74:76–84. 10.1016/j.ijnurstu.2017.06.003. 28641123 10.1016/j.ijnurstu.2017.06.003

[43] Partnership for Quality Measurement. Practice Environment Scale - Nursing Work Index (PES-NWI) (composite and five subscales) (previously NQF#0206 - Undergoing Maintenance). 2022. https://p4qm.org/measures/3450

[44] Büssing A, Perrar K-M. Die Messung von Burnout. Untersuchung einer deutschen Fassung des Maslach Burnout Inventory (MBI-D). [Measuring burnout: a study of a German version of the maslach burnout inventory (MBI-D).]. diagnostica. 1992:328–53. 8004334

[45] Maslach C, Jackson SE, Leiter M. January. The Maslach Burnout Inventory Manual. 1997. https://www.researchgate.net/publication/277816643_The_Maslach_Burnout_Inventory_Manual

[46] Maslach C, Schaufeli WB, Leiter MP. Job burnout. Annu Rev Psychol. 2001;52:397–422. 10.1146/annurev.psych.52.1.397. 11148311 10.1146/annurev.psych.52.1.397

[47] Maslach C. A multidimensional theory of burnout. In: Cooper CL, editor. Theories of organizational stress. Oxford University PressOxford; 1998. pp. 68–85. 10.1093/oso/9780198522799.003.0004.

[48] Doulougeri K, Georganta K, Montgomery A. Diagnosing burnout among healthcare professionals: can we find consensus? Cogent Med. 2016;3:1. 10.1080/2331205X.2016.1237605.

[49] Taber KS. The use of cronbach’s alpha when developing and reporting research instruments in science education. Res Sci Educ. 2018;48:1273–96. 10.1007/s11165-016-9602-2.

[50] van Buuren S, Groothuis-Oudshoorn K. Mice: multivariate imputation by chained equations in R. J Stat Soft. 2011. 10.18637/jss.v045.i03.

[51] van Buuren S. Flexible imputation of missing data. London, New York: CRC Press Taylor & Francis Group A Chapman & Hall Book;: Boca Raton; 2018.

[52] Rezvan PH, Comulada WS, Fernández MI, Belin TR. Assessing alternative imputation strategies for infrequently missing items on Multi-item scales. Commun Stat Case Stud Data Anal Appl. 2022;8:682–713. 10.1080/23737484.2022.2115430. 36467970 10.1080/23737484.2022.2115430 PMC9718541

[53] Bürkner P-C. Brms: an R package for Bayesian multilevel models using Stan. J Stat Soft. 2017. 10.18637/jss.v080.i01.

[54] Woods AD, Gerasimova D, van Dusen B, Nissen J, Bainter S, Uzdavines A, et al. Best practices for addressing missing data through multiple imputation. Infant Child Dev. 2024. 10.1002/icd.2407.

[55] Kruschke JK. Bayesian analysis reporting guidelines. Nat Hum Behav. 2021;5:1282–91. 10.1038/s41562-021-01177-7. 34400814 10.1038/s41562-021-01177-7 PMC8526359

[56] Cuschieri S. The STROBE guidelines. Saudi J Anaesth. 2019;13:S31–4. 10.4103/sja.SJA_543_18. 30930717 10.4103/sja.SJA_543_18 PMC6398292

[57] Wagner BZ-JF, Rzayeva N, Busse R. Strengthening health systems through nursing: Evidence from 14 European countries [Internet].: 4 Germany. 2019. https://www.ncbi.nlm.nih.gov/books/NBK545718/# 31465161

[58] Sommer D, Wilhelm S, Wahl F. Nurses’ workplace perceptions in Southern Germany-Job satisfaction and self-intended retention towards nursing. Healthc (Basel). 2024. 10.3390/healthcare12020172. 10.3390/healthcare12020172 PMC10815135 38255061

[59] Kumari A, Sarkar S, Ranjan P, Chopra S, Kaur T, Baitha U, et al. Interventions for workplace violence against health-care professionals: A systematic review. Work. 2022;73:415–27. 10.3233/WOR-210046. 35431213 10.3233/WOR-210046

[60] López-Ros P, López-López R, Pina D, Puente-López E. User violence prevention and intervention measures to minimize and prevent aggression towards health care workers: A systematic review. Heliyon. 2023;9:e19495. 10.1016/j.heliyon.2023.e19495. 37809629 10.1016/j.heliyon.2023.e19495 PMC10558594

[61] Mausz J, Braaksma M-J, Johnston M, Batt AM, Donnelly EA. Paramedic willingness to report violence following the introduction of a novel, Point-of-Event reporting process in a single Canadian paramedic service. Int J Environ Res Public Health. 2024. 10.3390/ijerph21030363. 38541360 10.3390/ijerph21030363 PMC10970047

[62] Thomas BJ, O’Meara P, Edvardsson K, Spelten E. Barriers and opportunities for workplace violence interventions in Australian paramedicine: A qualitative study. Australasian J Paramedicine. 2020;17:1–9. 10.33151/ajp.17.817.

[63] Arnetz JE, Hamblin L, Russell J, Upfal MJ, Luborsky M, Janisse J, Essenmacher L. Preventing Patient-to-Worker violence in hospitals: outcome of a randomized controlled intervention. J Occup Environ Med. 2017;59:18–27. 10.1097/JOM.0000000000000909. 28045793 10.1097/JOM.0000000000000909 PMC5214512

[64] Somani R, Muntaner C, Hillan E, Velonis AJ, Smith PA, Systematic Review. Effectiveness of interventions to De-escalate workplace violence against nurses in healthcare settings. Saf Health Work. 2021;12:289–95. 10.1016/j.shaw.2021.04.004. 34527388 10.1016/j.shaw.2021.04.004 PMC8430427

[65] Spelten E, Thomas B, O’Meara PF, Maguire BJ, FitzGerald D, Begg SJ. Organisational interventions for preventing and minimising aggression directed towards healthcare workers by patients and patient advocates. Cochrane Database Syst Rev. 2020;4:CD012662. 10.1002/14651858.CD012662.pub2. 32352565 10.1002/14651858.CD012662.pub2 PMC7197696

[66] Lee M, Cha C. Interventions to reduce burnout among clinical nurses: systematic review and meta-analysis. Sci Rep. 2023;13:10971. 10.1038/s41598-023-38169-8. 37414811 10.1038/s41598-023-38169-8 PMC10325963

[67] Kunzler AM, Helmreich I, Chmitorz A, König J, Binder H, Wessa M, Lieb K. Psychological interventions to foster resilience in healthcare professionals. Cochrane Database Syst Rev. 2020;7:CD012527. 10.1002/14651858.cd012527.pub2. 32627860 10.1002/14651858.CD012527.pub2 PMC8121081

[68] O’Brien CJ, van Zundert AAJ, Barach PR. The growing burden of workplace violence against healthcare workers: trends in prevalence, risk factors, consequences, and prevention - a narrative review. EClinicalMedicine. 2024;72:102641. 10.1016/j.eclinm.2024.102641. 38840669 10.1016/j.eclinm.2024.102641 PMC11152903

[69] BMG. Schärfere Strafen bei Gewalt gegen Ärzte. 19. Feb. 2020. https://www.bundesgesundheitsministerium.de/ministerium/meldungen/2020/schaerfere-strafen-bei-gewalt-gegen-aerzte

[70] Merecz D, Rymaszewska J, Mościcka A, Kiejna A, Jarosz-Nowak J. Violence at the workplace–a questionnaire survey of nurses. Eur Psychiatry. 2006;21:442–50. 10.1016/j.eurpsy.2006.01.001. 16530389 10.1016/j.eurpsy.2006.01.001

[71] Rahama EES, Elmadani M, Mohammed MM, Osman Eqrer ZA. Occupational violence against nursing staff in the surgical wards of Murzuq locality hospitals, Libya (2024). BMC Nurs. 2025;24:222. 10.1186/s12912-025-02870-y 10.1186/s12912-025-02870-y PMC11866610 40016728

[72] Jakobsson J, Örmon K, Axelsson M, Berthelsen H. Exploring workplace violence on surgical wards in sweden: a cross-sectional study. BMC Nurs. 2023;22:106. 10.1186/s12912-023-01275-z. 37029387 10.1186/s12912-023-01275-z PMC10079490

[73] Winstanley S, Whittington R. Anxiety, burnout and coping styles in general hospital staff exposed to workplace aggression: A cyclical model of burnout and vulnerability to aggression. Work Stress. 2002;16:302–15. 10.1080/0267837021000058650.

[74] Liu J, Zheng J, Liu K, Liu X, Wu Y, Wang J, You L. Workplace violence against nurses, job satisfaction, burnout, and patient safety in Chinese hospitals. Nurs Outlook. 2019;67:558–66. 10.1016/j.outlook.2019.04.006. 31202444 10.1016/j.outlook.2019.04.006

[75] O’Connell B, Young J, Brooks J, Hutchings J, Lofthouse J. Nurses’ perceptions of the nature and frequency of aggression in general ward settings and high dependency areas. J Clin Nurs. 2000;9:602–10. 10.1046/j.1365-2702.2000.00401.x. 11261143 10.1046/j.1365-2702.2000.00401.x

[76] Havaei F, MacPhee M. The impact of heavy nurse workload and patient/family complaints on workplace violence: an application of human factors framework. Nurs Open. 2020;7:731–41. 10.1002/nop2.444. 32257260 10.1002/nop2.444 PMC7113506

[77] Kahsay WG, Negarandeh R, Dehghan Nayeri N, Hasanpour M. Sexual harassment against female nurses: a systematic review. BMC Nurs. 2020;19:58. 10.1186/s12912-020-00450-w. 32612455 10.1186/s12912-020-00450-w PMC7324991

[78] Tversky A, Kahneman D. Availability: A heuristic for judging frequency and probability. In: Kahneman D, Slovic P, Tversky A, editors. Judgment under uncertainty. Cambridge University Press; 2013. pp. 163–78. 10.1017/CBO9780511809477.012.

[79] Saragih ID, Tarihoran DETAU, Rasool A, Saragih IS, Tzeng H-M, Lin C-J. Global prevalence of stigmatization and violence against healthcare workers during the COVID-19 pandemic: a systematic review and meta-analysis. J Nurs Scholarsh. 2022;54:762–71. 10.1111/jnu.12794. 35819267 10.1111/jnu.12794 PMC9349814

[80] Spencer C, Sitarz J, Fouse J, DeSanto K. Nurses’ rationale for underreporting of patient and visitor perpetrated workplace violence: a systematic review. BMC Nurs. 2023;22:134. 10.1186/s12912-023-01226-8. 37088834 10.1186/s12912-023-01226-8 PMC10122798

[81] Baka L, Prusik M, Jasielska D. Toward a better Understanding of the health impairment process. Types of demand and burnout component matter. Front Psychiatry. 2022;13:1037053. 10.3389/fpsyt.2022.1037053. 36699490 10.3389/fpsyt.2022.1037053 PMC9868600

[82] Chen C, Meier ST. Burnout and depression in nurses: A systematic review and meta-analysis. Int J Nurs Stud. 2021;124:104099. 10.1016/j.ijnurstu.2021.104099. 34715576 10.1016/j.ijnurstu.2021.104099

[83] van Ginkel JR, Linting M, Rippe RCA, van der Voort A. Rebutting existing misconceptions about multiple imputation as a method for handling missing data. J Pers Assess. 2020;102:297–308. 10.1080/00223891.2018.1530680. 30657714 10.1080/00223891.2018.1530680

[84] Tutz G. Ordinal regression: a review and a taxonomy of models. WIREs Comput Stats. 2022. 10.1002/wics.1545.

