# Supplementary material for: Impact of violence on emotional exhaustion risk of registered nurses in Germany: a Bayesian analysis of cross-sectional data with multiple imputations
Source: BMC Nurs. 2025 Aug 18;24:1080. doi: 10.1186/s12912-025-03745-y (PMC12359958; doi:10.1186/s12912-025-03745-y)

Appendix A – Figures, predictive matrices, priors and convergence

Appendix A1 – MI prediction matrix

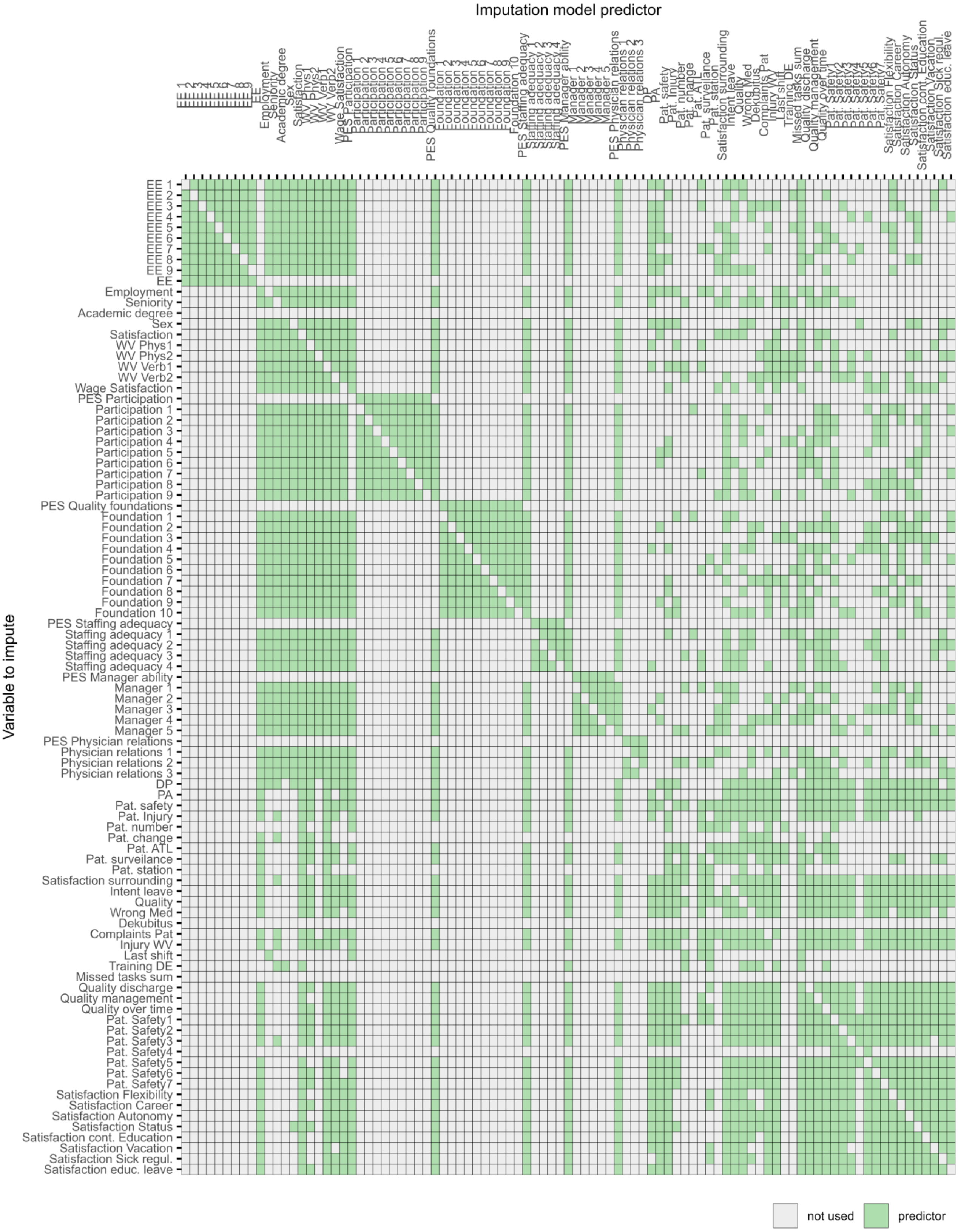

Prediction matrix showing which predictors were selected for multiple imputation of RNs data

Appendix A2 – Formalization and visualization of prior distributions

All priors are formalized and plotted below (Prior set 1: green; Prior set 2: red; Prior set 3: blue)

$$\beta_p \sim N(\mu_p, \sigma_p) \rightarrow$$

$$\beta_p \sim t(df, \mu_p, \sigma_p) \rightarrow$$

$$\beta_p \sim N(0,1.5) \rightarrow$$

Sex-Female Prior
Reference level= Male vs. Female

$\beta_{Sex\ Female} \sim N(0.25,0.15)$
$\beta_{Sex\ Female} \sim t(2,0.25,0.15)$
$\beta_{flat} \sim N(0,1.5)$

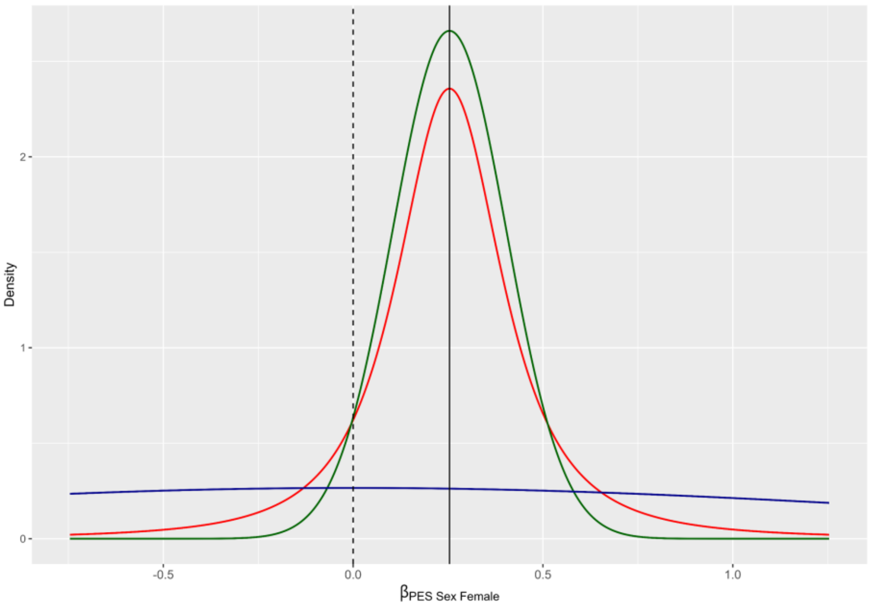

Employment Prior
Nursing: Reference level= Part-time vs. Full-time
EMS: Reference level= Part-time or volunteer vs. Full time

$\beta_{Employment} \sim N(0.654,0.3)$
$\beta_{Employment} \sim t(2,0.654,0.3)$
$\beta_{flat} \sim N(0,1.5)$

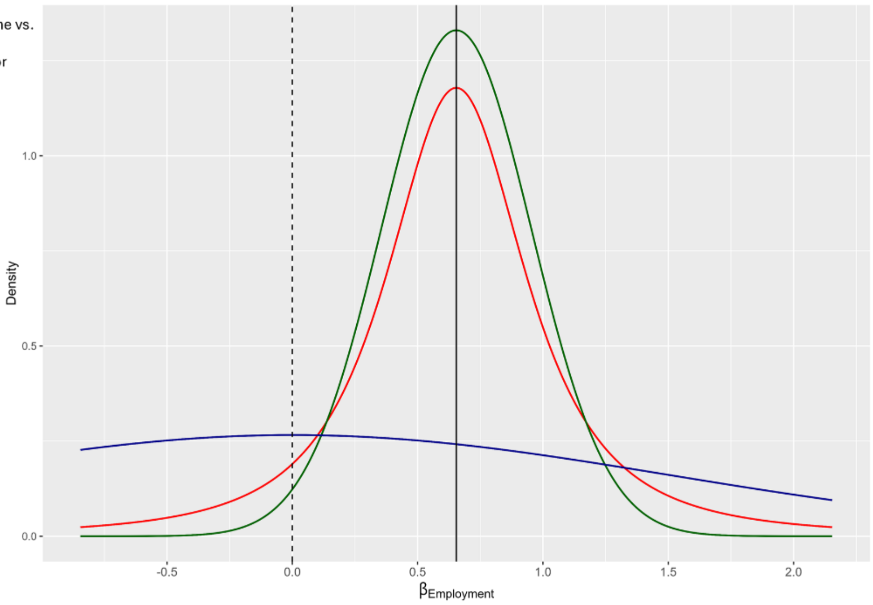

Seniority Prior
Seniority in years

$\beta_{Seniority} \sim N(-0.004, 0.2)$
$\beta_{Seniority} \sim t(2, -0.004, 0.2)$
$\beta_{flat} \sim N(0, 1.5)$

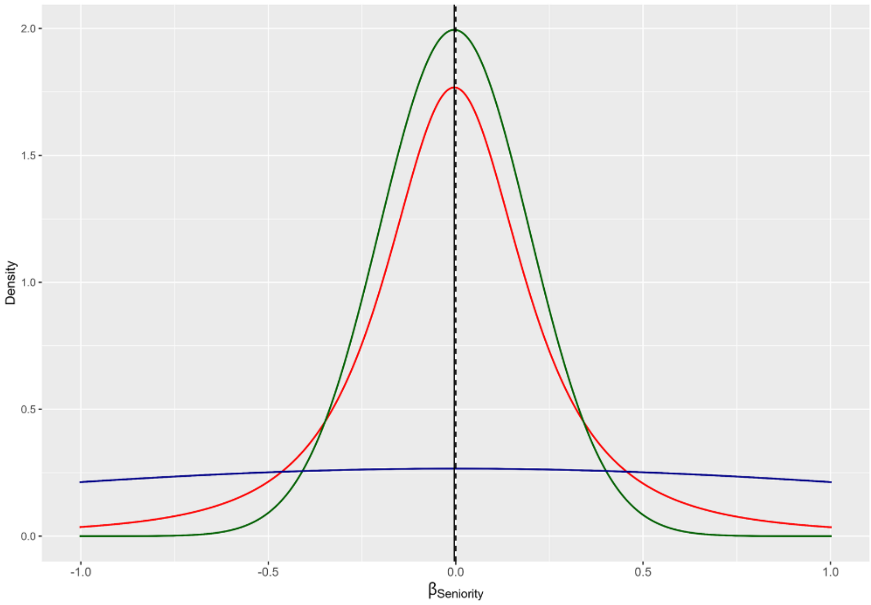

Wage Satisfaction Prior
Reference level= Negative vs. Positive Wage Satisfaction

$\beta_{Wage\ Satisfaction} \sim N(-0.3, 0.15)$
$\beta_{Wage\ Satisfaction} \sim t(2, -0.3, 0.15)$
$\beta_{flat} \sim N(0, 1.5)$

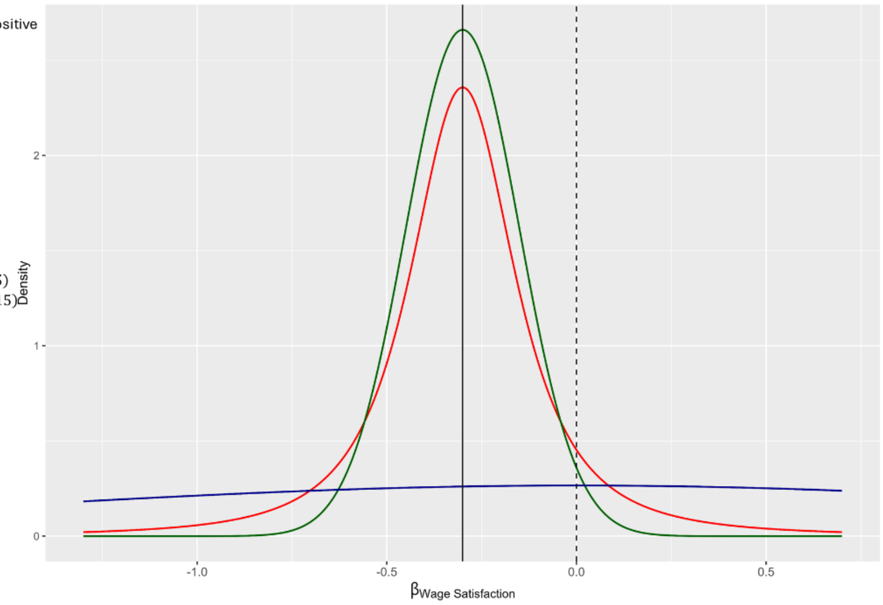

Academic degree Prior
Reference level= No vs. Yes academic nursing-related degree

$\beta_{Satisfaction} \sim N(-0.12, 0.15)$
$\beta_{Satisfaction} \sim t(2, -0.12, 0.15)$
$\beta_{flat} \sim N(0, 1.5)$

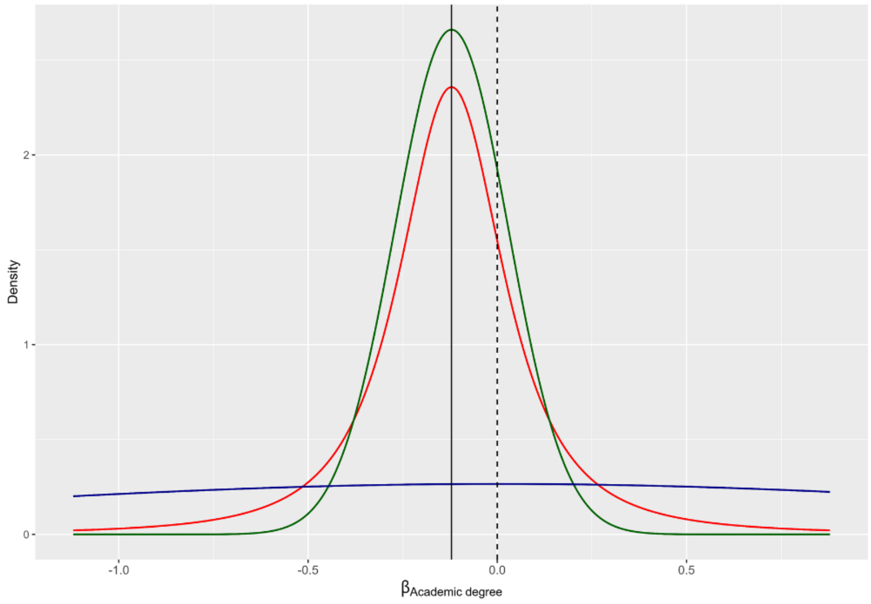

Prior distributions (continued)

PES Manager ability Priors
Reference level= Negative vs. Positive PES Manager ability

$\beta_{PES\ Manager\ ability} \sim N(-0.35, 0.2)$
$\beta_{PES\ Manager\ ability} \sim t(2, -0.35, 0.2)$
$\beta_{flat} \sim N(0, 1.5)$

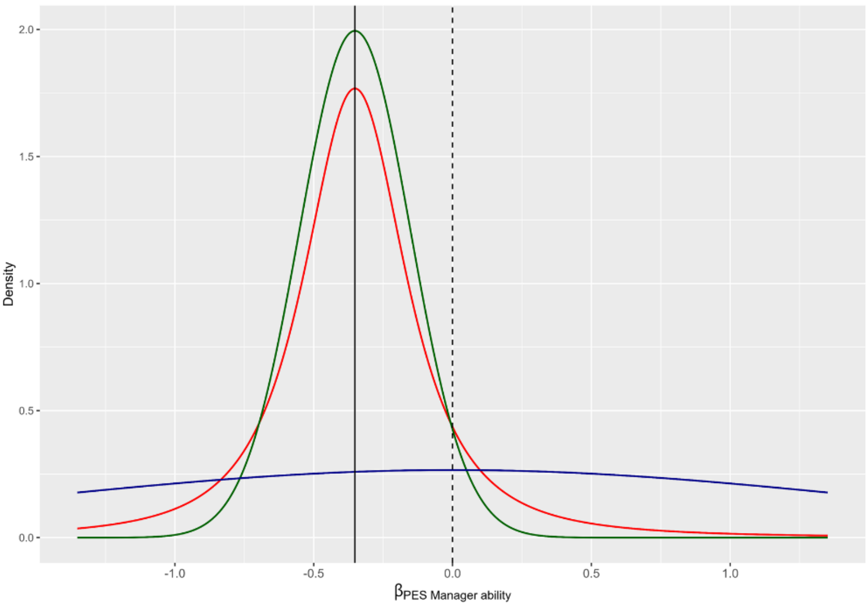

PES Quality Foundations Priors
Reference level= Negative vs. Positive PES Quality foundations

$\beta_{PES\ Quality\ foundations} \sim N(-0.55, 0.25)$
$\beta_{PES\ Quality\ foundations} \sim t(2, -0.55, 0.25)$
$\beta_{flat} \sim N(0, 1.5)$

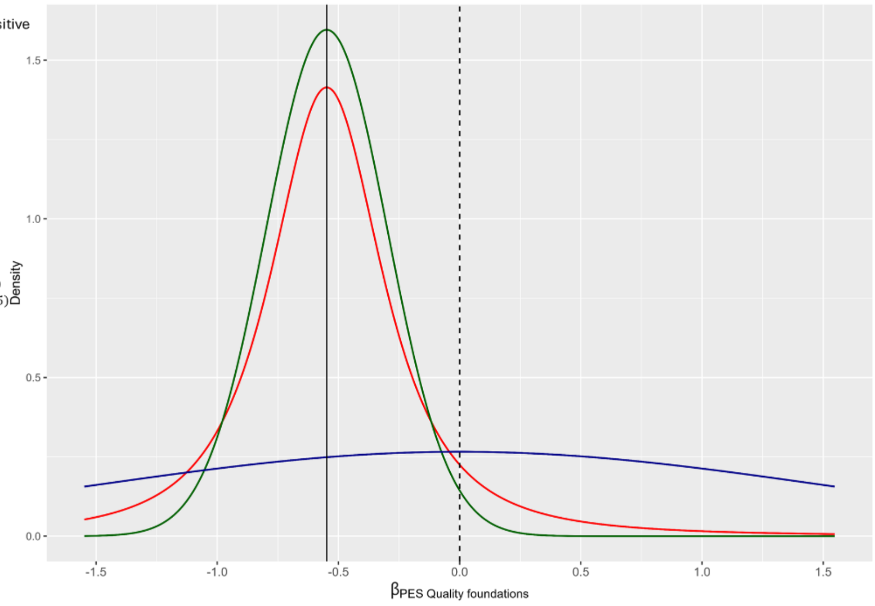

PES Physician relations Prior
Reference level= Negative vs. Positive PES Physician relations

$\beta_{PES\ Physician\ relations} \sim N(-0.43, 0.2)$
$\beta_{PES\ Physician\ relations} \sim t(2, -0.43, 0.2)$
$\beta_{flat} \sim N(0, 1.5)$

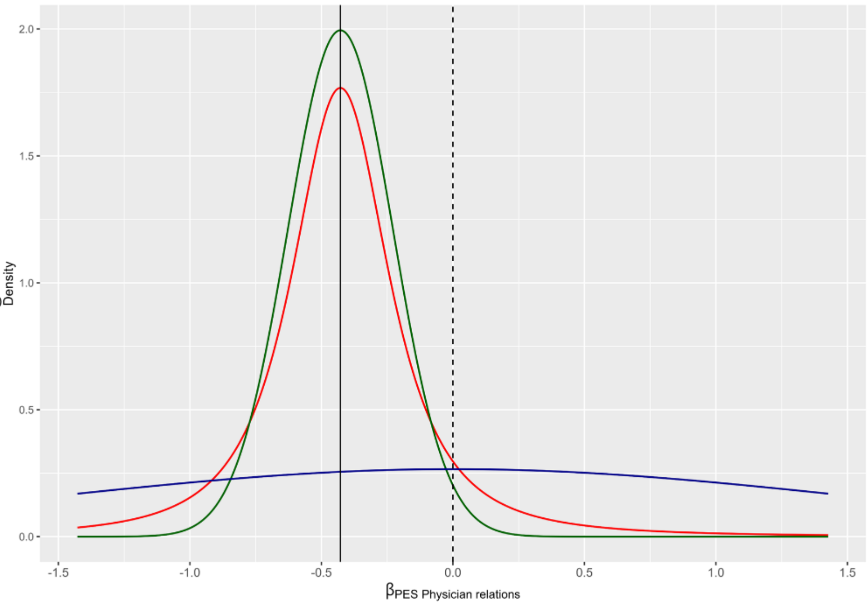

Prior distributions (continued)

PES Staffing adequacy Prior
Reference level= Negative vs. Positive PES Staffings adequacy

$\beta_{PES\ Staffing\ adequacy} \sim N(-0.46, 0.2)$
$\beta_{PES\ Staffing\ adequacy} \sim t(2, -0.46, 0.2)$
$\beta_{flat} \sim N(0, 1.5)$

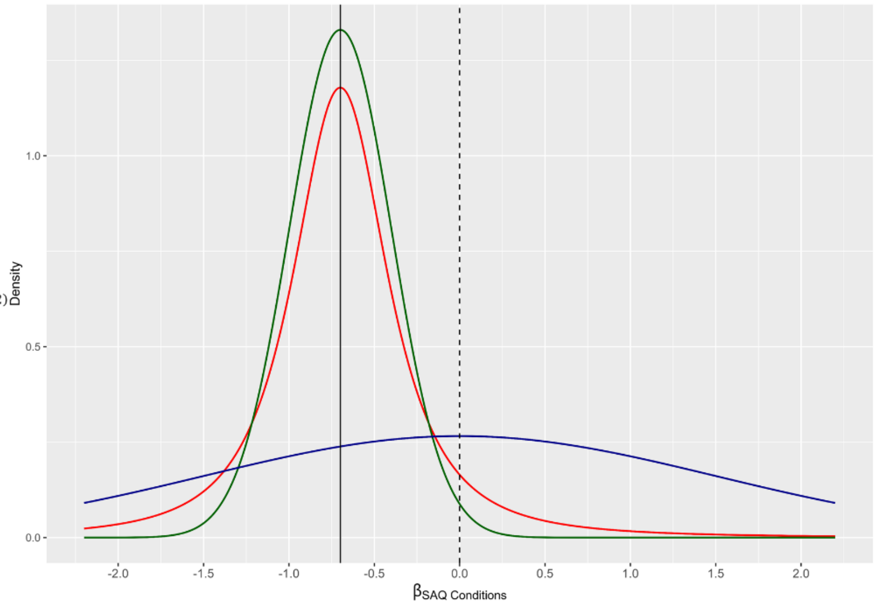

PES Participation Priors
Reference level= Negative vs. Positive PES Participation

$\beta_{PES\ Participation} \sim N(-0.72, 0.35)$
$\beta_{PES\ Participation} \sim t(2, -0.72, 0.35)$
$\beta_{flat} \sim N(0, 1.5)$

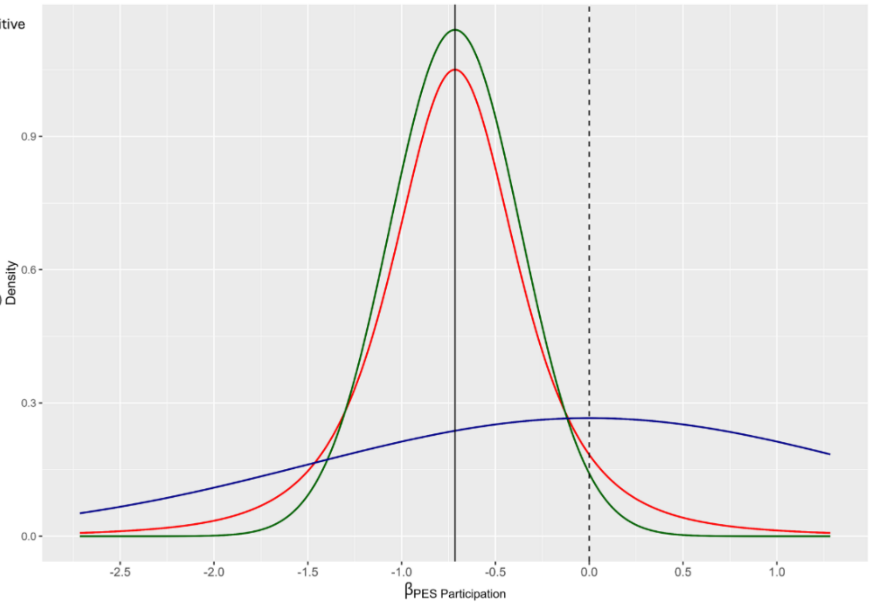

WV Verbal 1 – Moderate Prior
Reference level= Low vs. Moderate WV Verb1

$\beta_{WV\ Verb1\ moderate} \sim N(0.41, 0.2)$
$\beta_{WV\ Verb1\ moderate} \sim t(2, 0.41, 0.2)$
$\beta_{flat} \sim N(0, 1.5)$

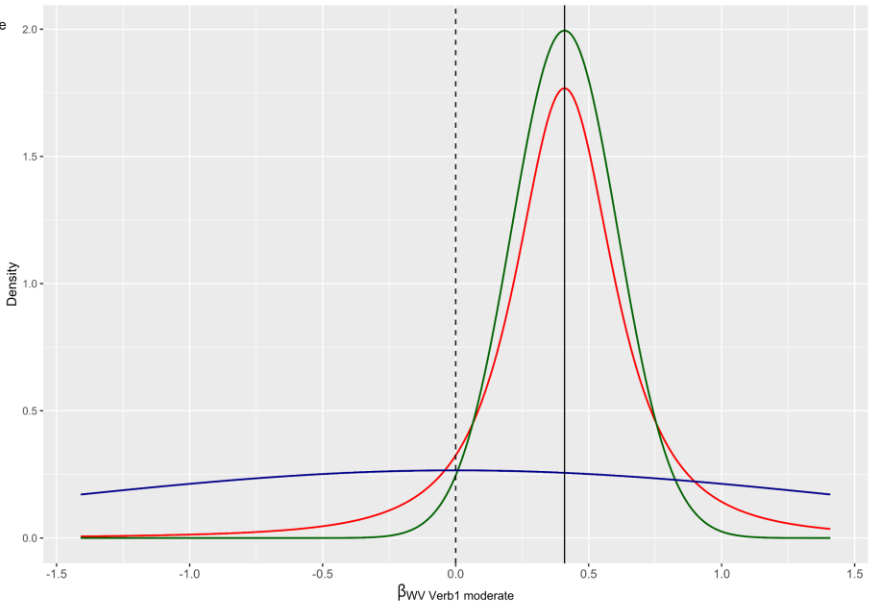

Prior distributions (continued)

WV Verbal 1 – High Prior
Reference level= Low vs. High WV Verb1

$\beta_{WV\ Verb1\ high} \sim N(0.49, 0.2)$
$\beta_{WV\ Verb1\ high} \sim t(2, 0.49, 0.2)$
$\beta_{flat} \sim N(0, 1.5)$

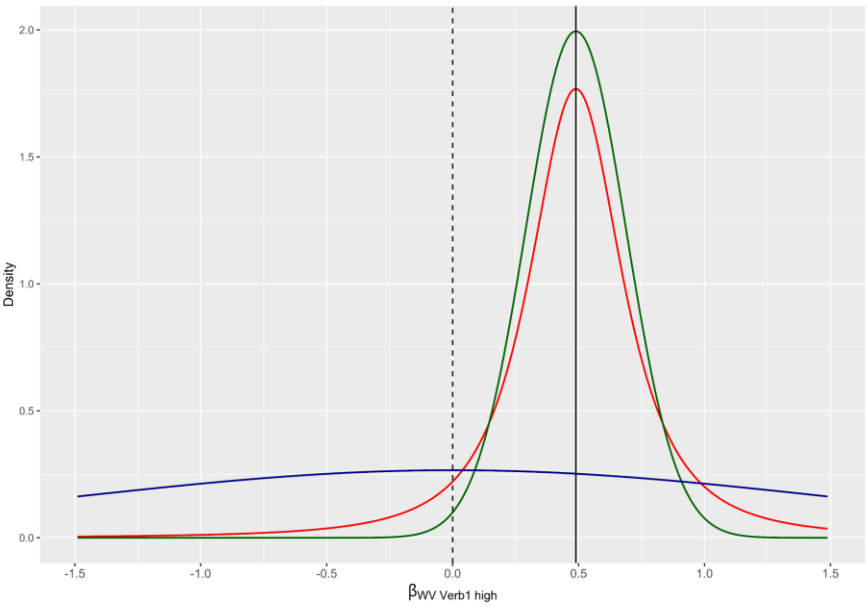

WV Verbal 2 – Moderate Prior
Reference level= Low vs. Moderate Verb2

$\beta_{WV\ Verb2\ moderate} \sim N(0.35, 0.2)$
$\beta_{WV\ Verb2\ moderate} \sim t(2, 0.35, 0.2)$
$\beta_{flat} \sim N(0, 1.5)$

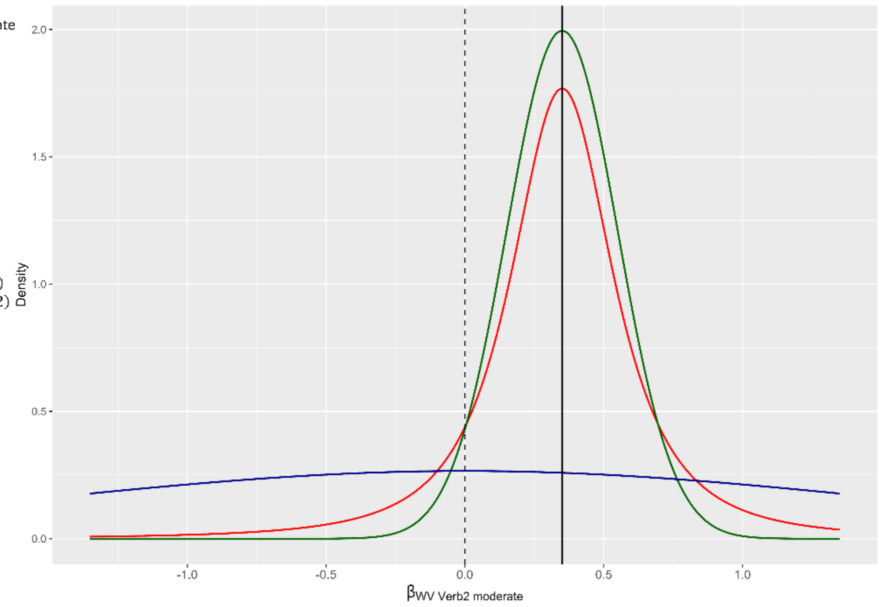

WV Verbal 2 – High Prior
Reference level= Low vs. High WV Verb2

$\beta_{WV\ Verb2\ high} \sim N(0.41, 0.2)$
$\beta_{WV\ Verb2\ high} \sim t(2, 0.41, 0.2)$
$\beta_{flat} \sim N(0, 1.5)$

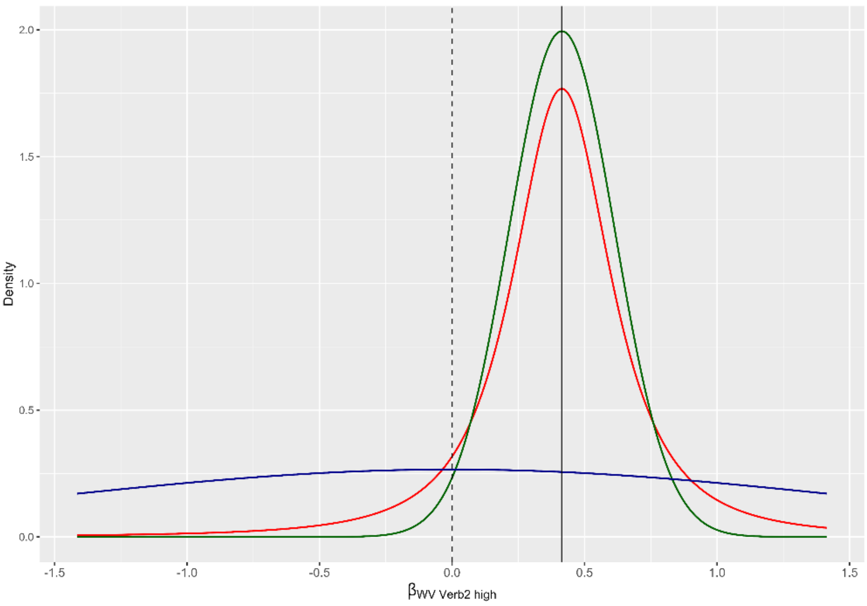

Prior distributions (continued)

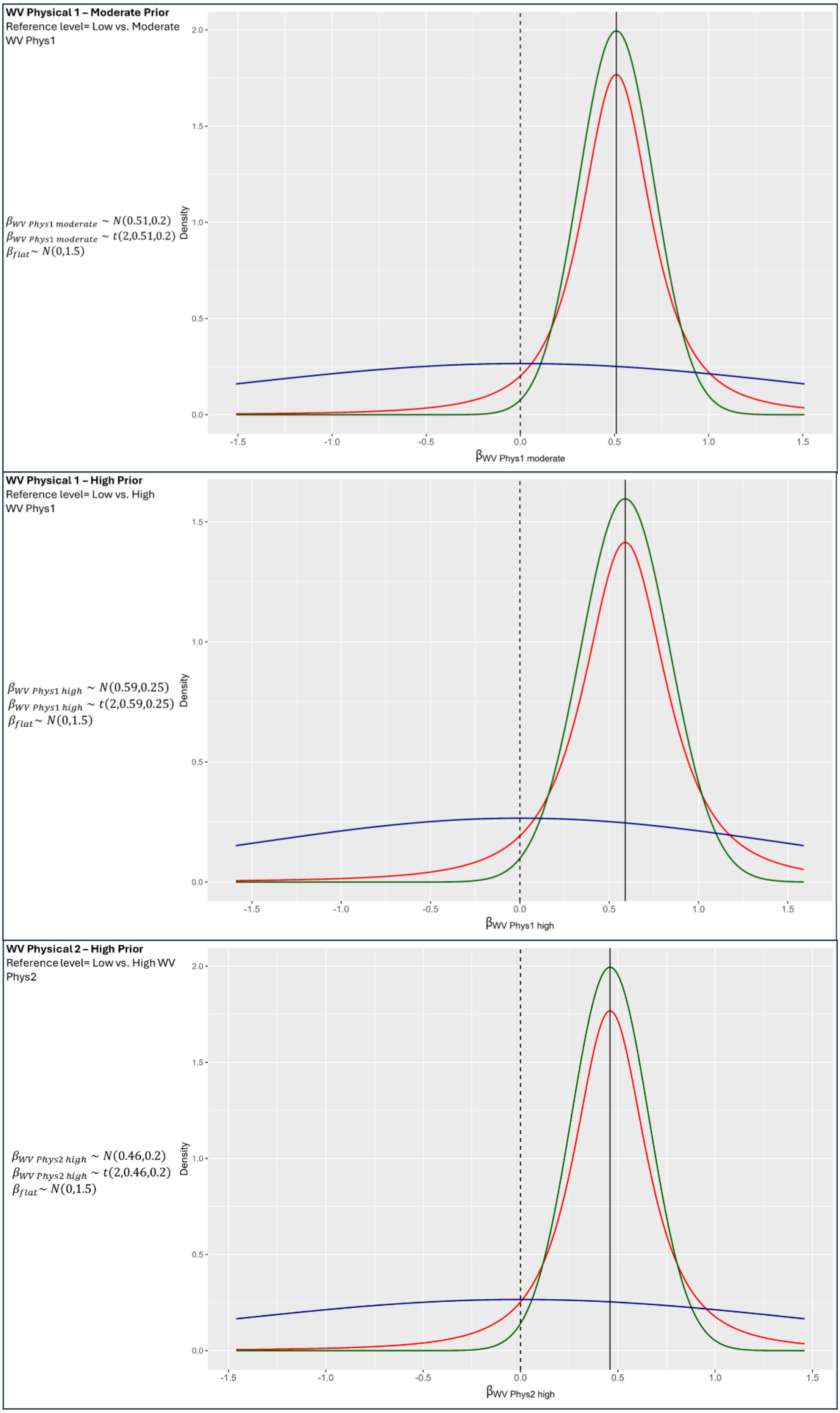

Prior distributions (continued)

Appendix A3 – PSRF and trace plots for MI of RN analysis variables

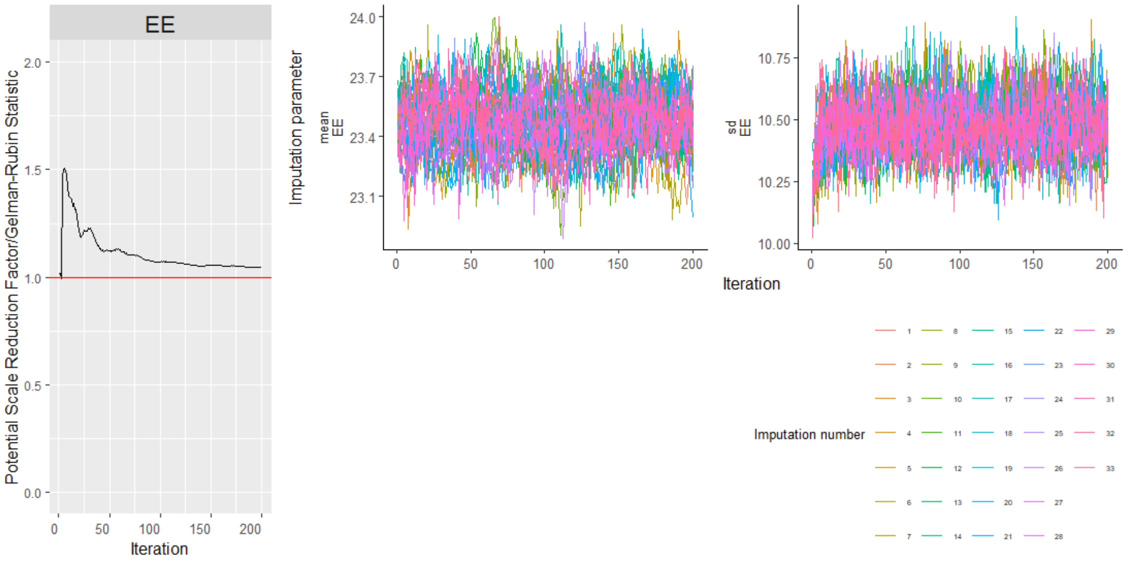

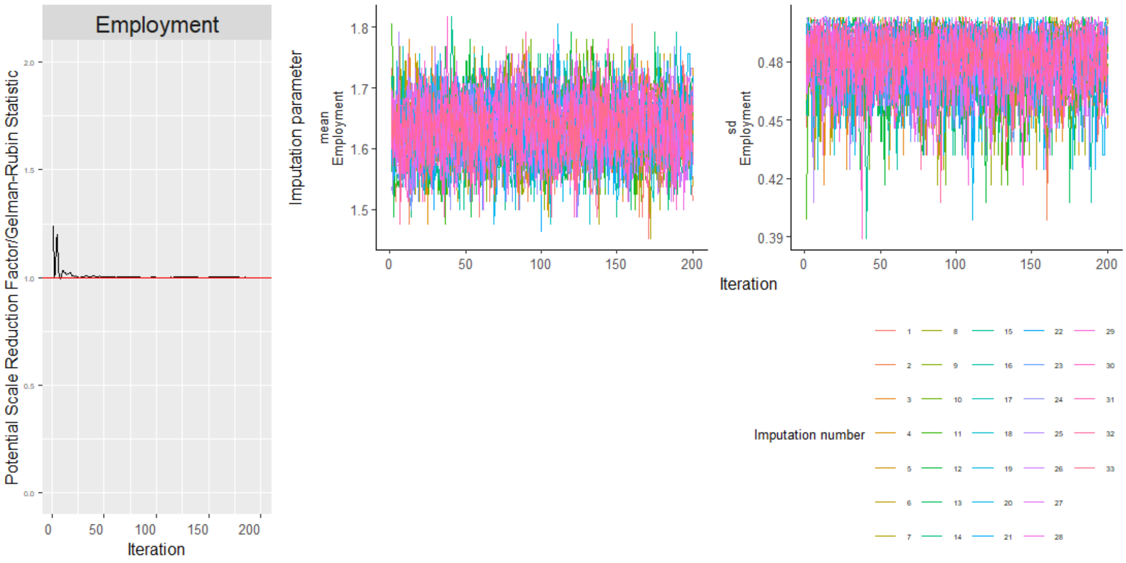

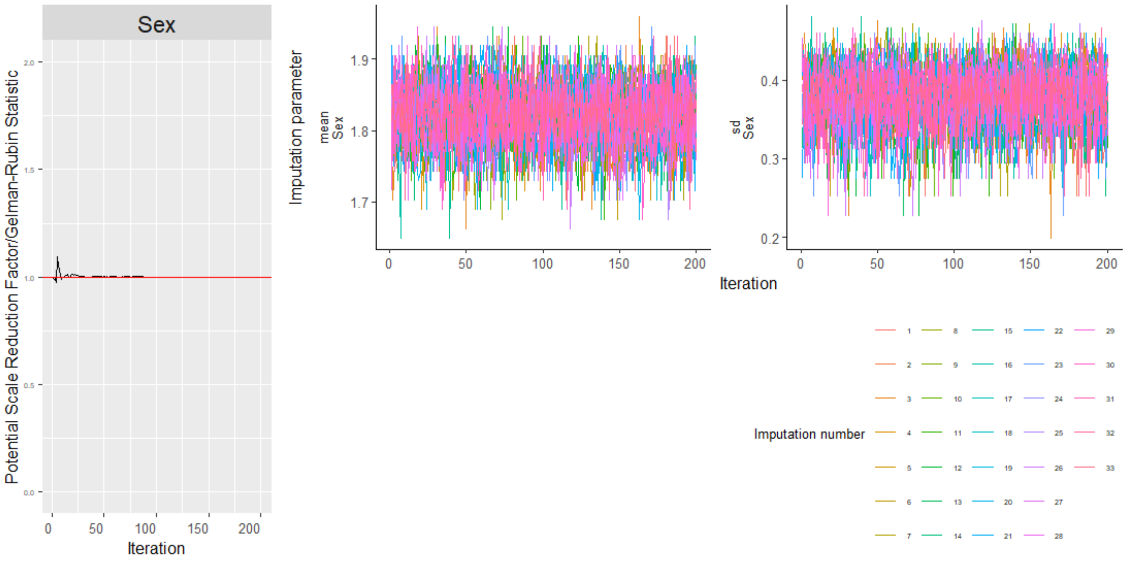

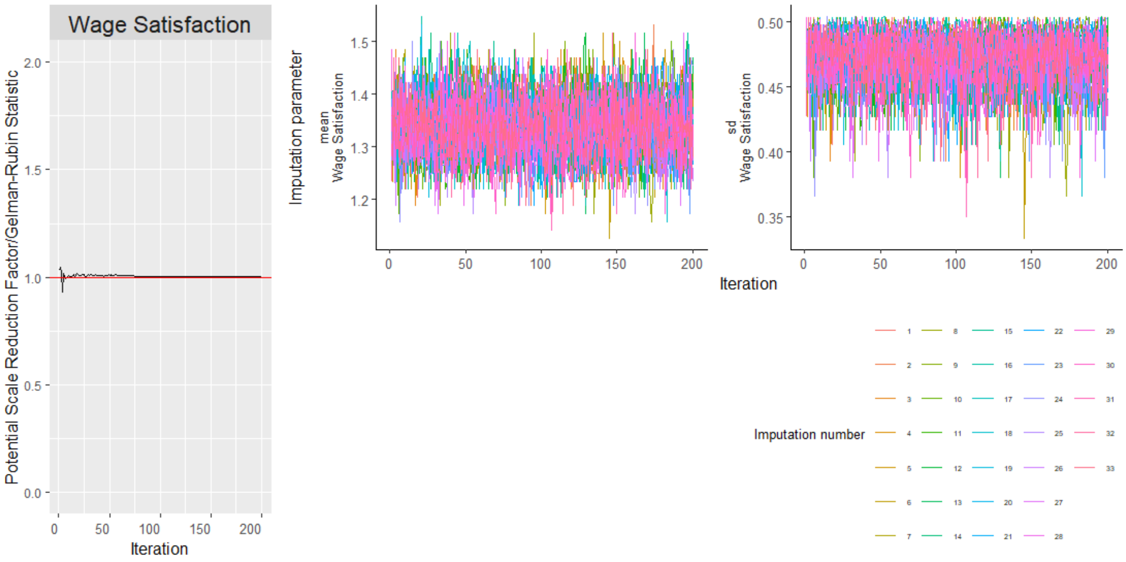
Wage Satisfaction
Potential Scale Reduction Factor/Gelman-Rubin Statistic
Iteration
Imputation parameter
mean
Wage Satisfaction
sd
Wage Satisfaction
Iteration
Imputation number

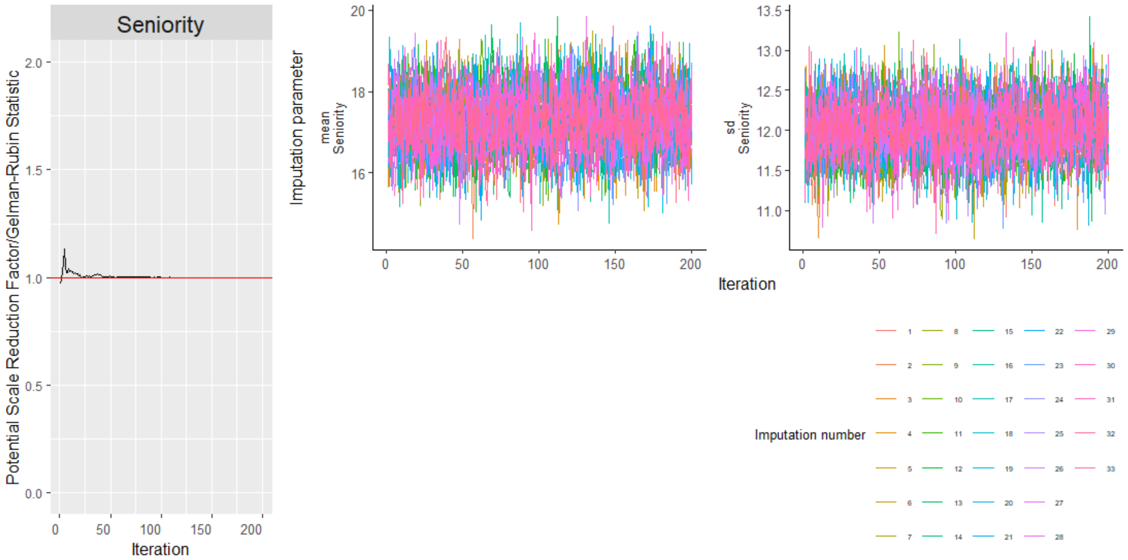
Seniority
Potential Scale Reduction Factor/Gelman-Rubin Statistic
Iteration
Imputation parameter
mean
Seniority
sd
Seniority
Iteration
Imputation number

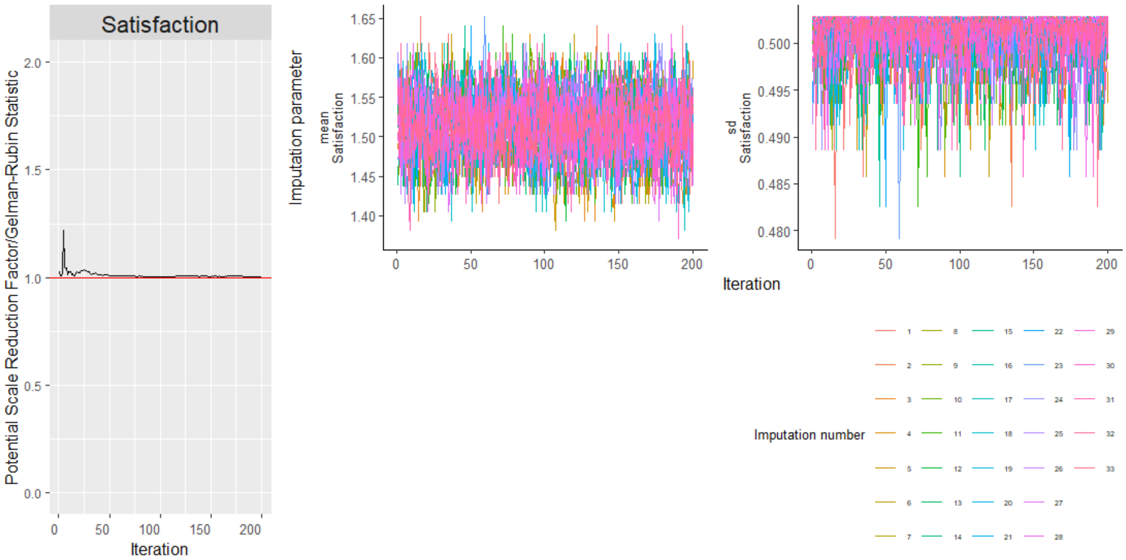
Satisfaction
Potential Scale Reduction Factor/Gelman-Rubin Statistic
Iteration
Imputation parameter
mean
Satisfaction
sd
Satisfaction
Iteration
Imputation number

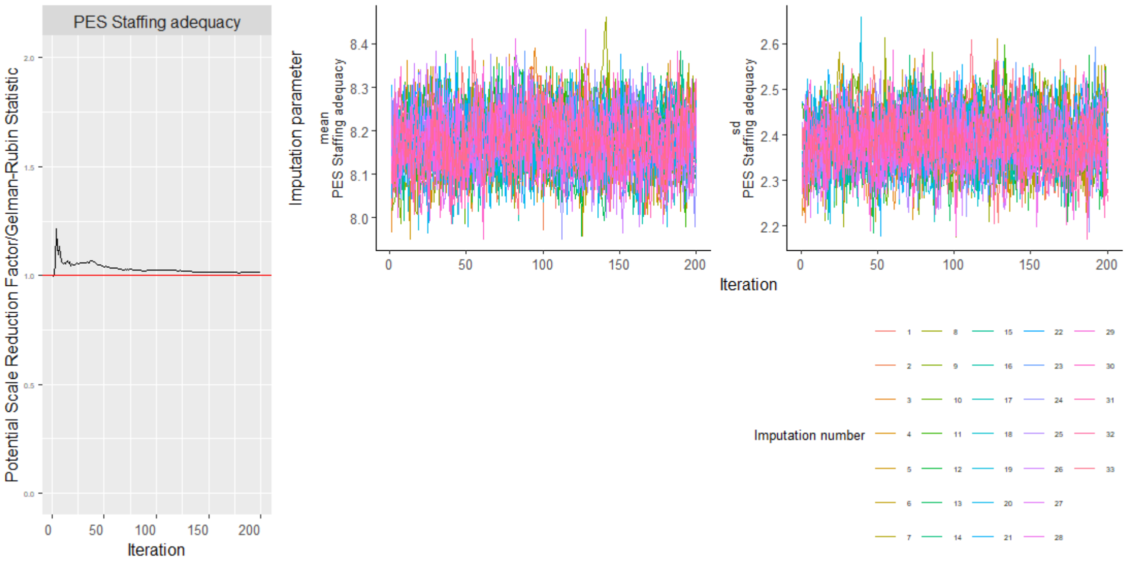
PES Staffing adequacy
Potential Scale Reduction Factor/Gelman-Rubin Statistic
Iteration
Imputation parameter
mean
PES Staffing adequacy
sd
PES Staffing adequacy
Iteration
Imputation number

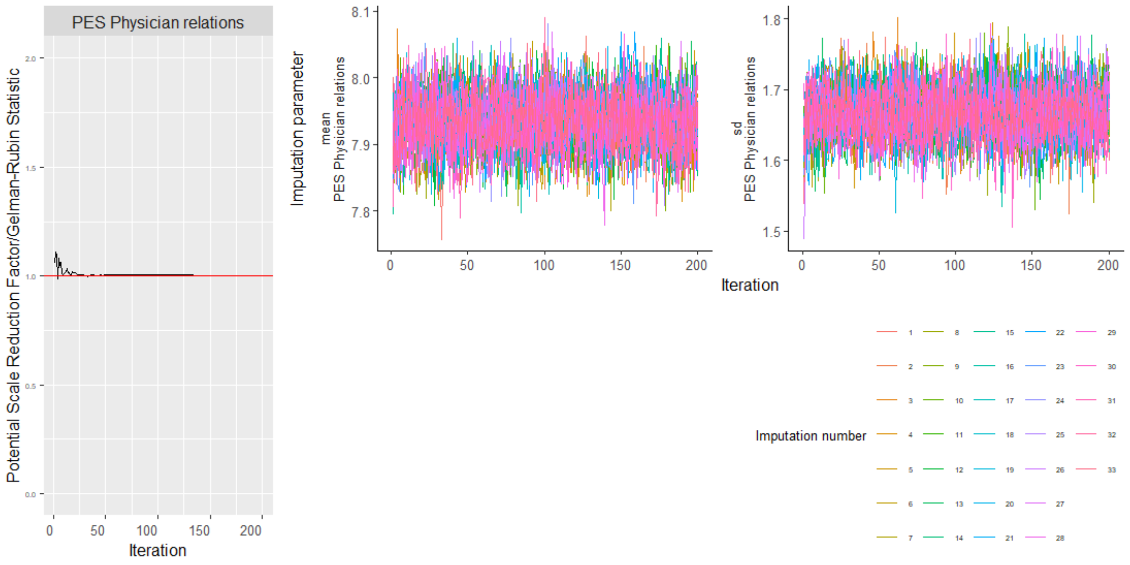
PES Physician relations
Potential Scale Reduction Factor/Gelman-Rubin Statistic
Iteration
Imputation parameter
mean
PES Physician relations
sd
PES Physician relations
Iteration
Imputation number

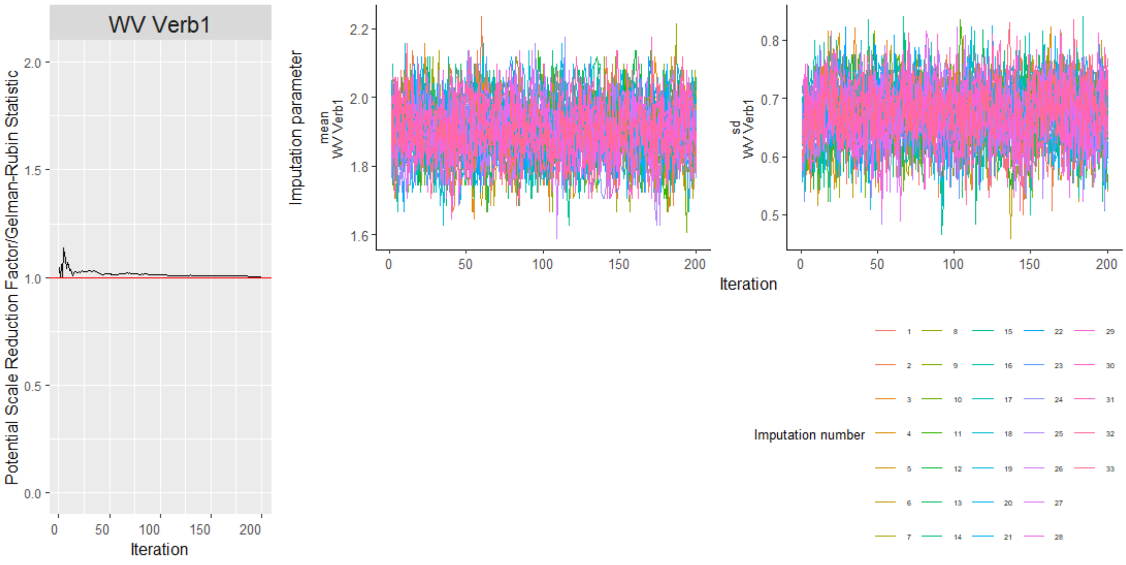
WV Verb1
Potential Scale Reduction Factor/Gelman-Rubin Statistic
Iteration
Imputation parameter
mean
WV Verb1
sd
WV Verb1
Iteration
Imputation number

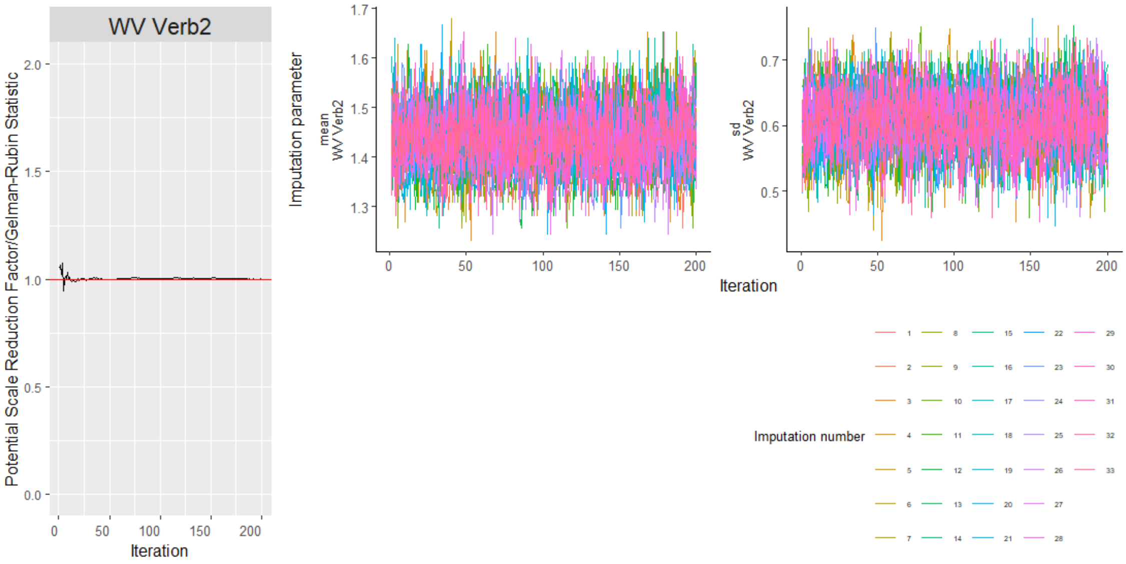
WV Verb2
Potential Scale Reduction Factor/Gelman-Rubin Statistic
Iteration
Imputation parameter
mean WV Verb2
sd WV Verb2
Imputation number

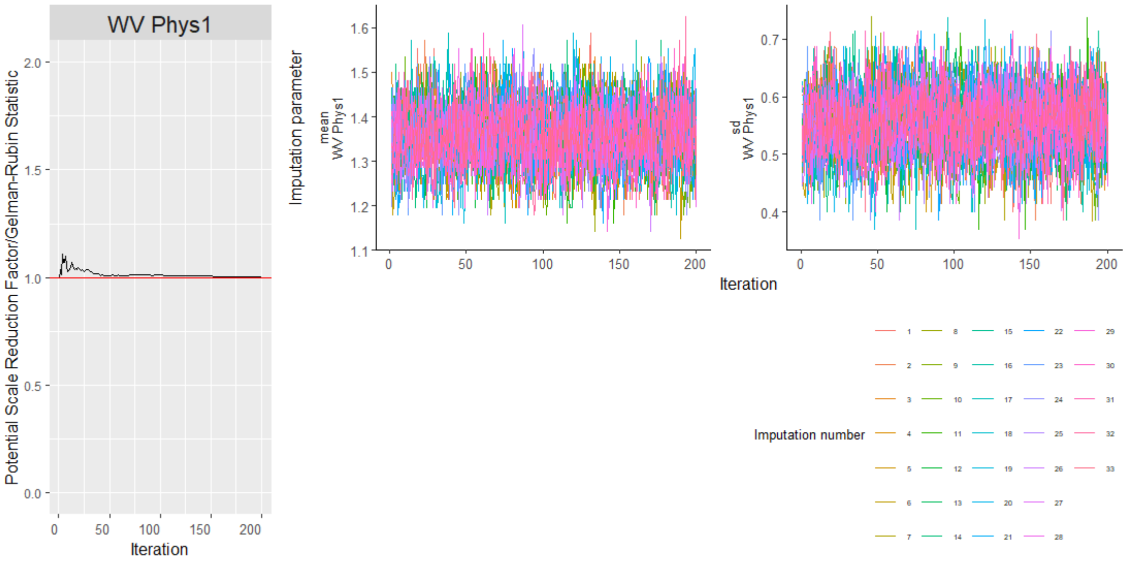
WV Phys1
Potential Scale Reduction Factor/Gelman-Rubin Statistic
Iteration
Imputation parameter
mean WV Phys1
sd WV Phys1
Imputation number

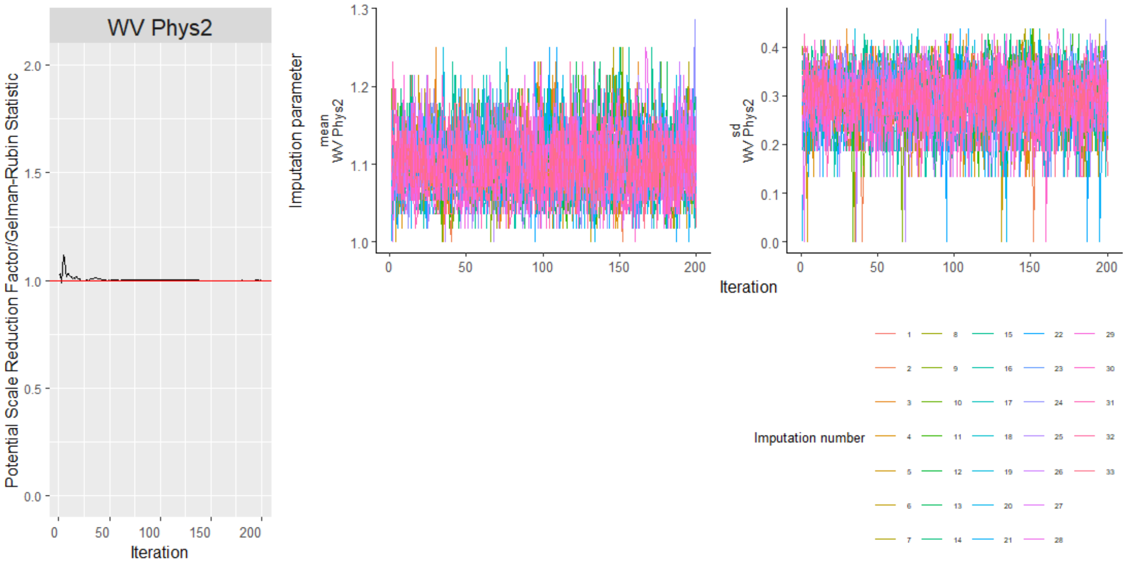
WV Phys2
Potential Scale Reduction Factor/Gelman-Rubin Statistic
Iteration
Imputation parameter
mean WV Phys2
sd WV Phys2
Imputation number

Appendix A4 – Prior predictive checks

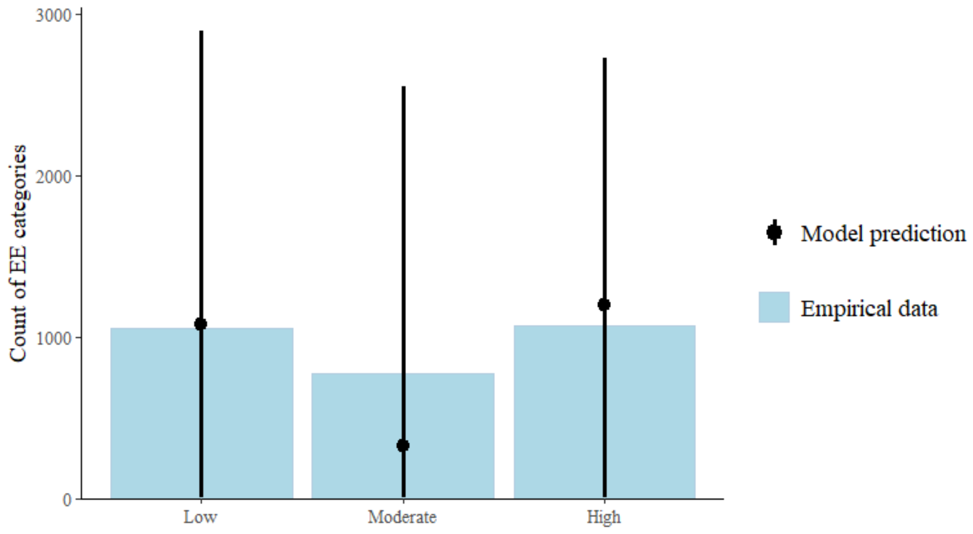

Prior predictive check, prior set 1

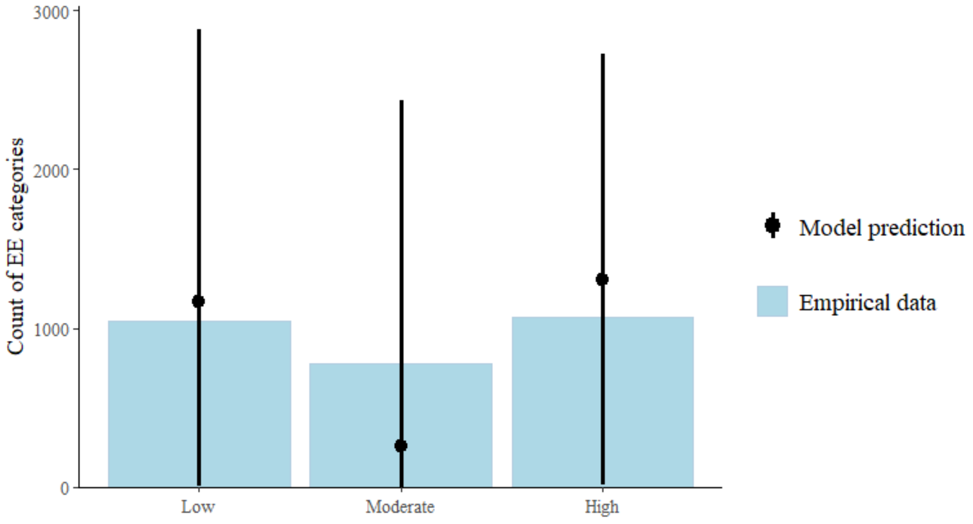

Prior predictive check, prior set 2

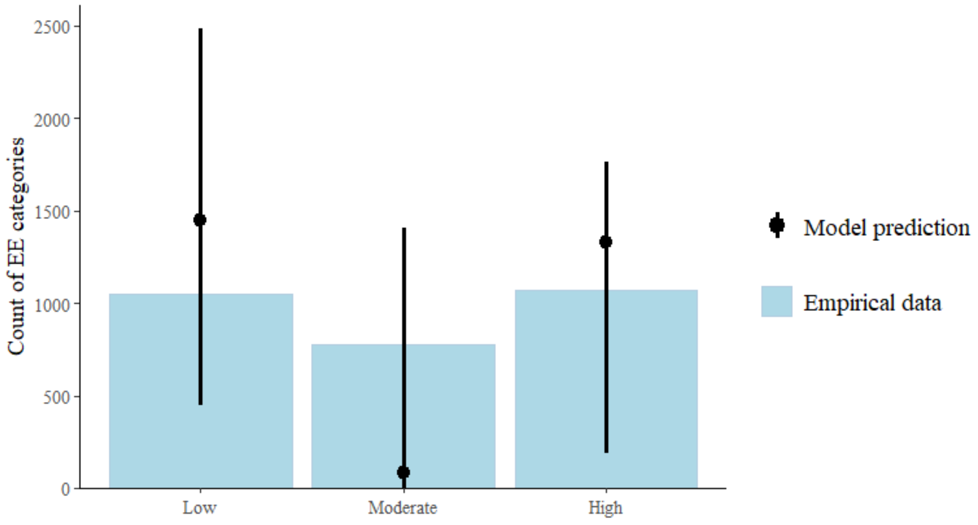

Prior predictive check, prior set 3

Appendix A5 – Posterior predictive checks

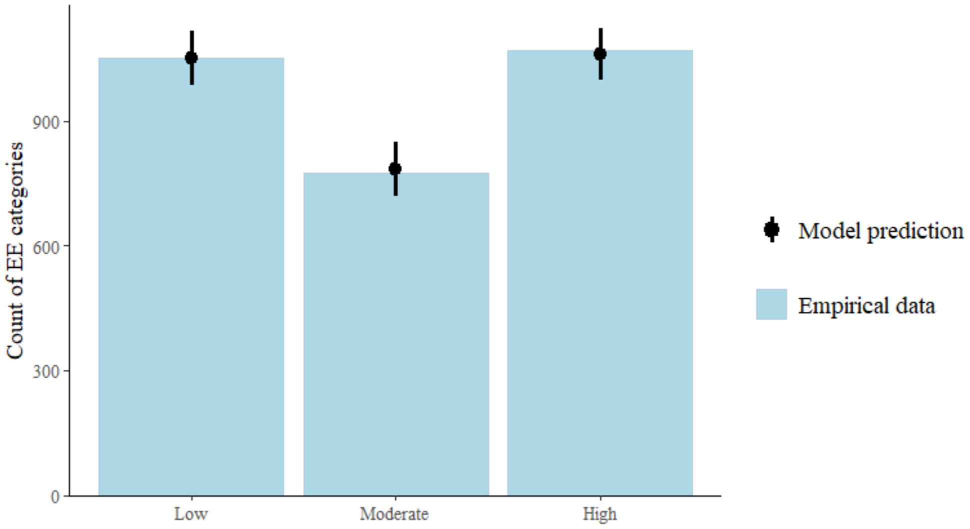

Posterior predictive check, prior set 1

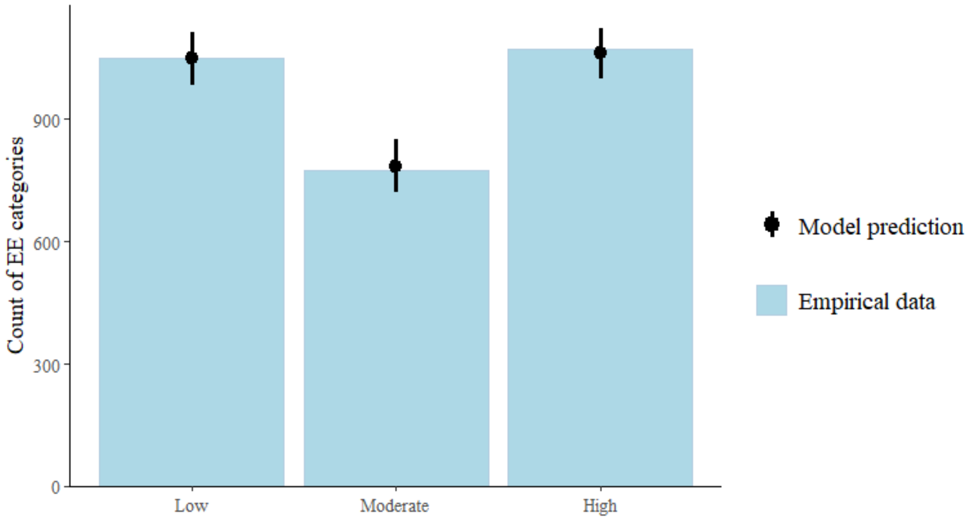

Posterior predictive check, prior set 2

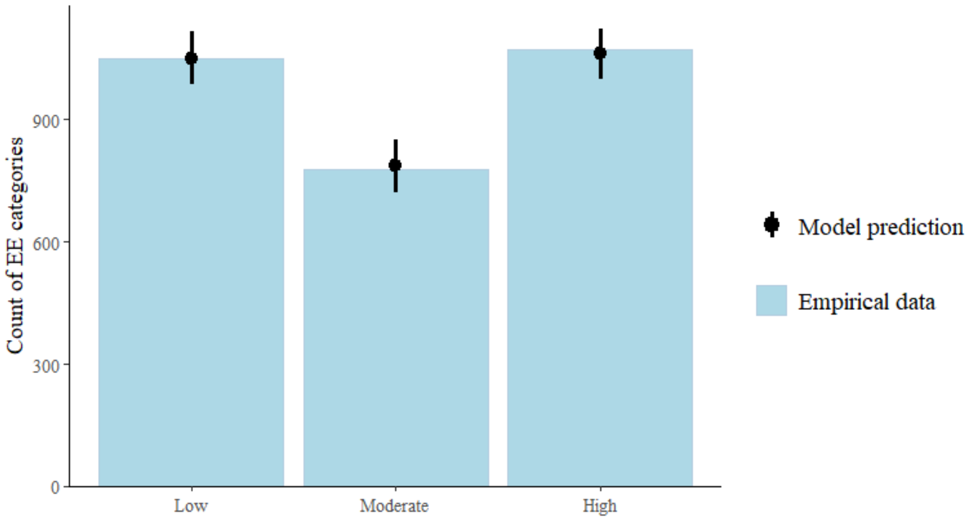

Posterior predictive check, prior set 3

Appendix A6 – RN posterior of MI analysis

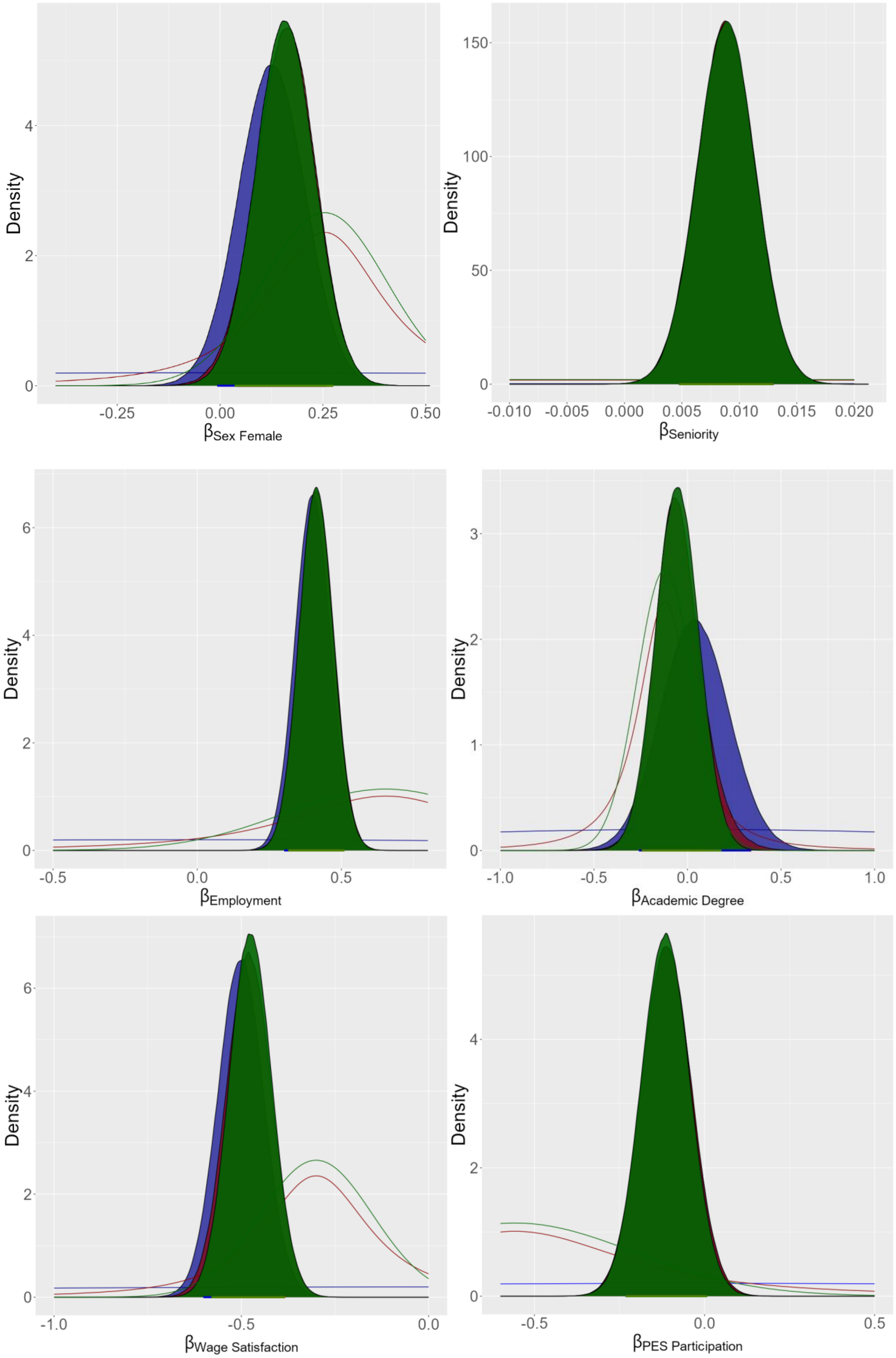

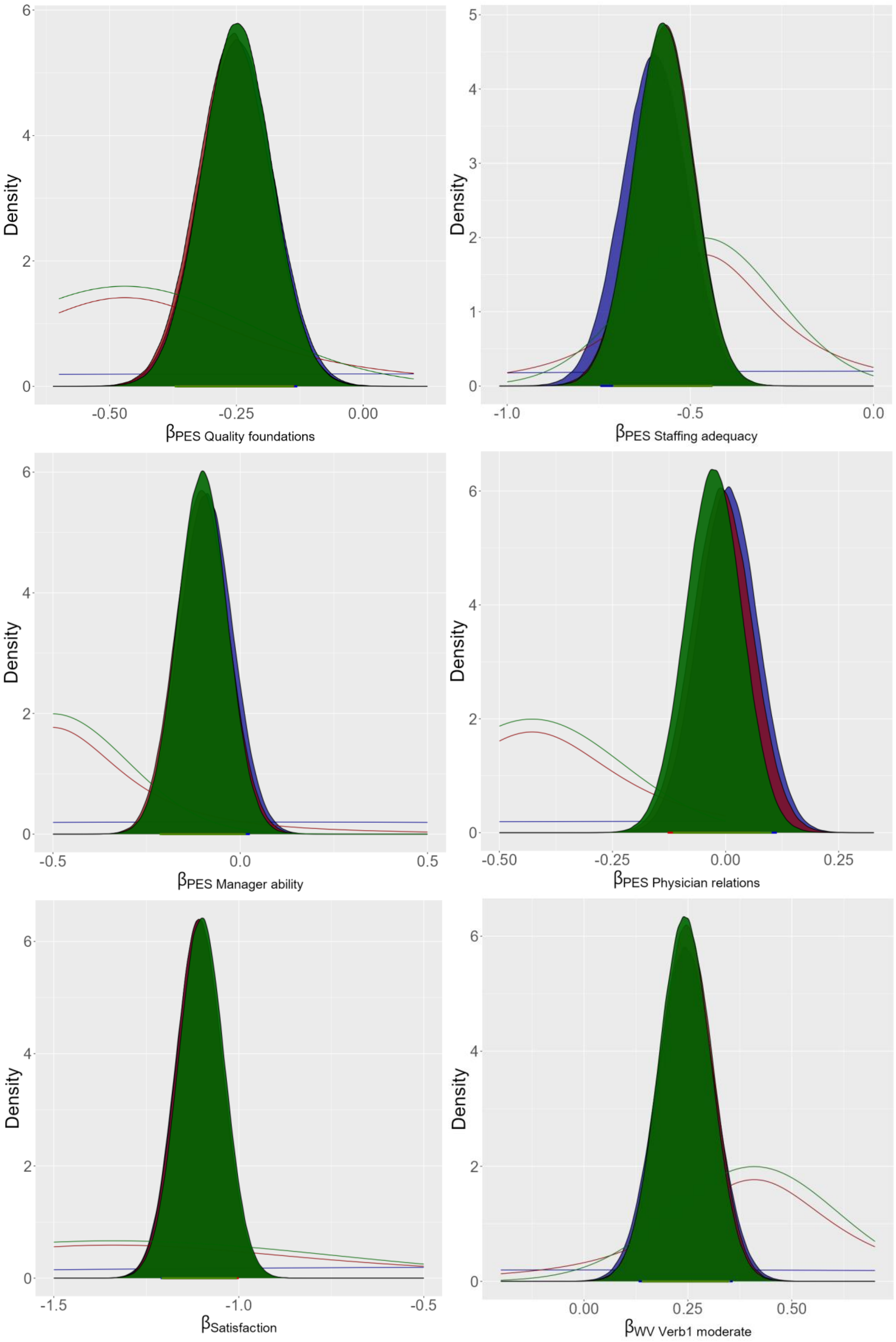
Density
-0.50
-0.25
0.00
$\beta_{\text{PES Quality foundations}}$
-1.0
-0.5
0.0
$\beta_{\text{PES Staffing adequacy}}$
-0.5
0.0
0.5
$\beta_{\text{PES Manager ability}}$
-0.50
-0.25
0.00
0.25
$\beta_{\text{PES Physician relations}}$
-1.5
-1.0
-0.5
$\beta_{\text{Satisfaction}}$
0.00
0.25
0.50
$\beta_{\text{WV Verb1 moderate}}$

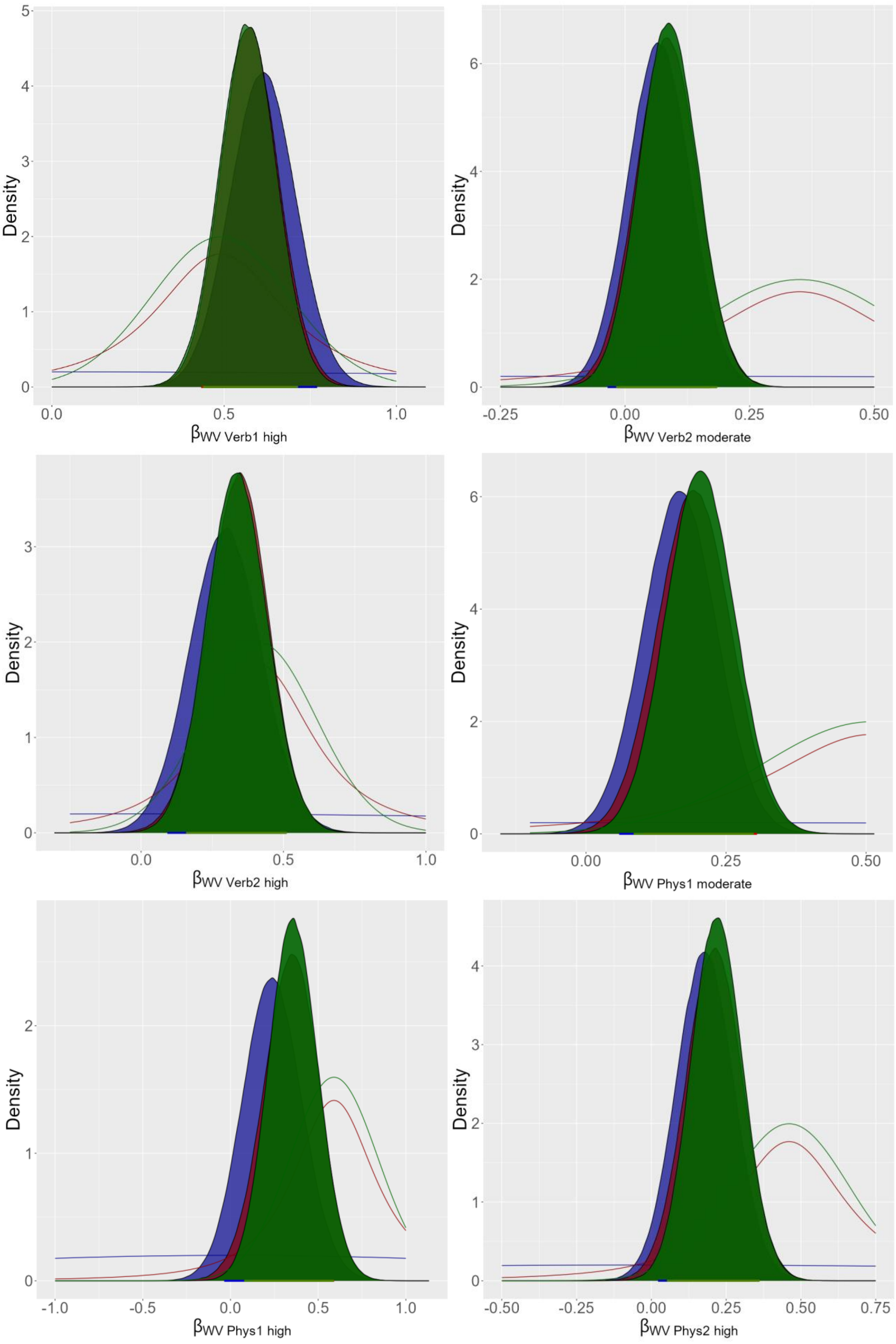
Density
βWV Verb1 high
0
1
2
3
4
5
0.0
0.5
1.0
Density
βWV Verb2 moderate
0
2
4
6
-0.25
0.00
0.25
0.50
Density
βWV Verb2 high
0
1
2
3
0.0
0.5
1.0
Density
βWV Phys1 moderate
0
2
4
6
0.00
0.25
0.50
Density
βWV Phys1 high
0
1
2
-1.0
-0.5
0.0
0.5
1.0
Density
βWV Phys2 high
0
1
2
3
4
-0.50
-0.25
0.00
0.25
0.50
0.75

Appendix A7 – RN Convergence CC

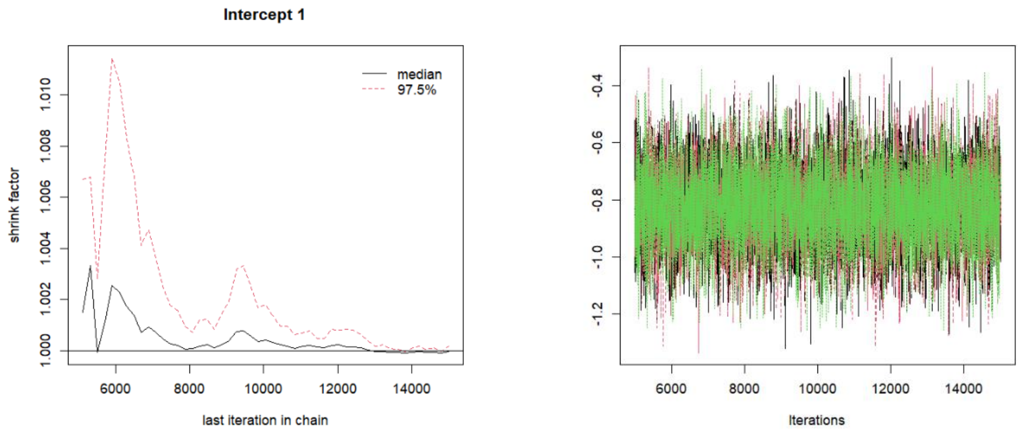

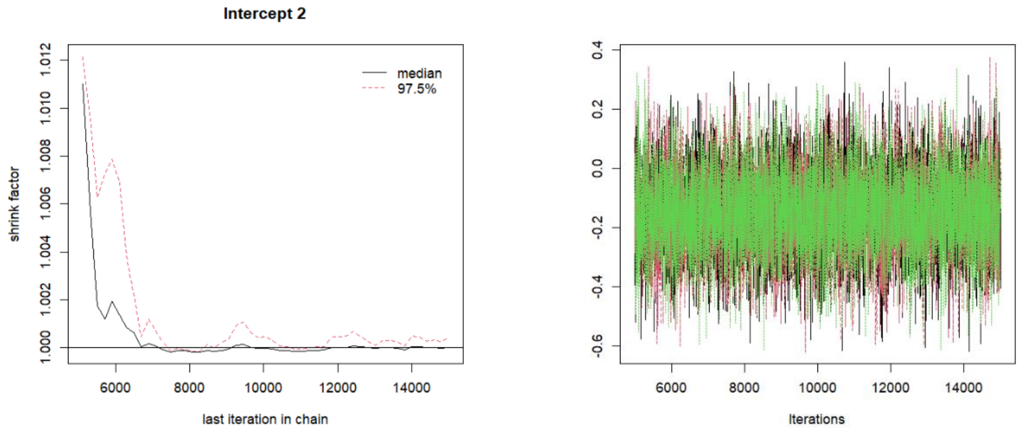

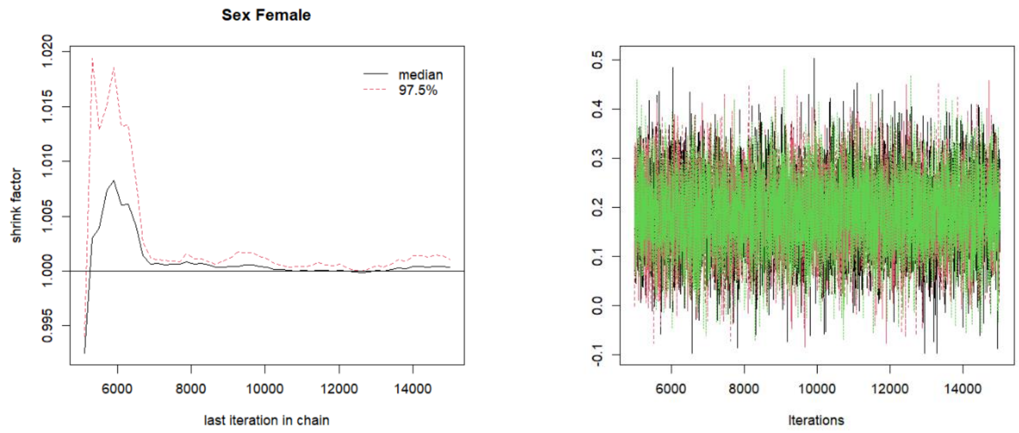

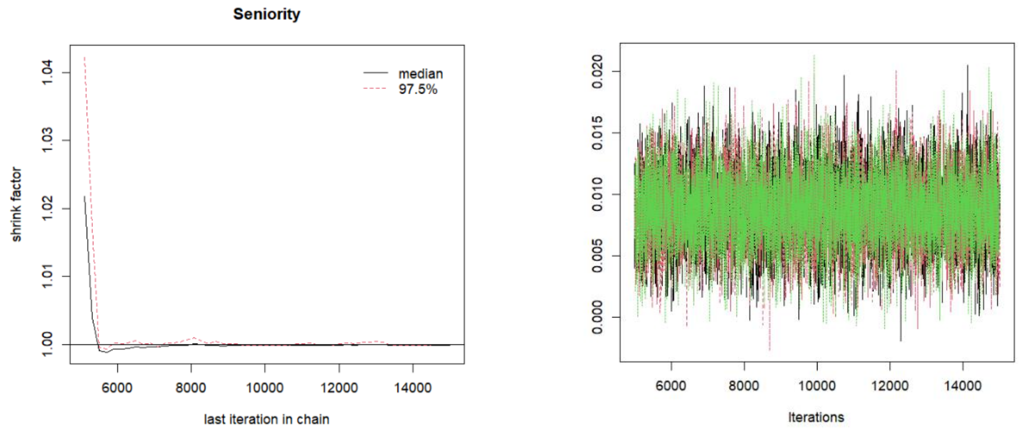

Employment Fulltime

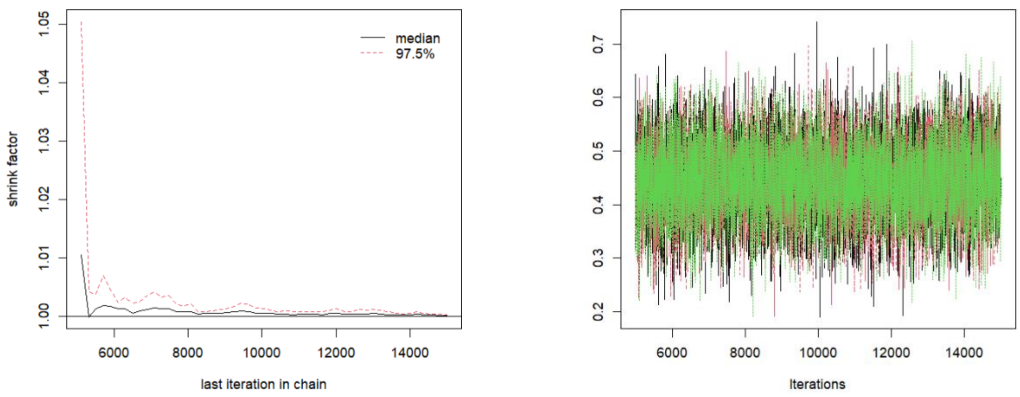

Academic degree

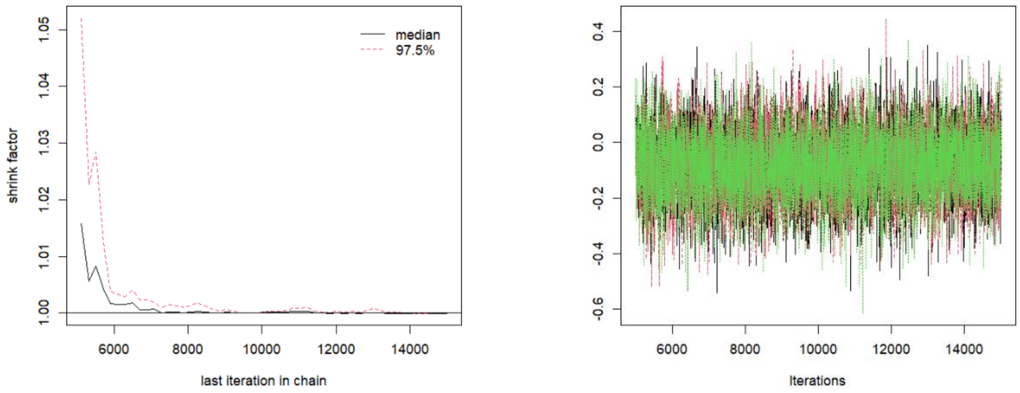

Wage Satisfaction

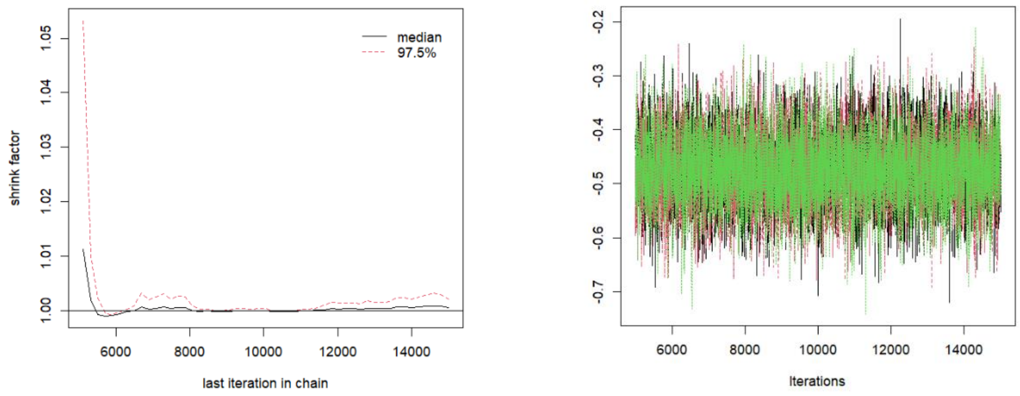

Seniority

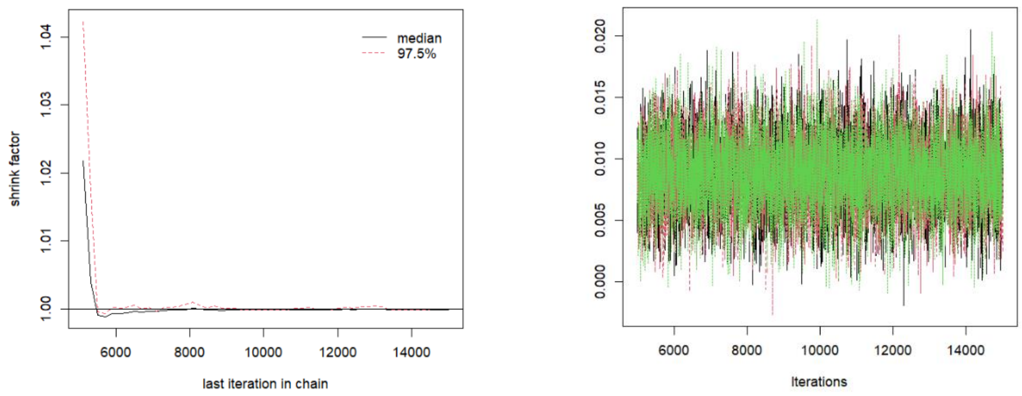

PES Participation

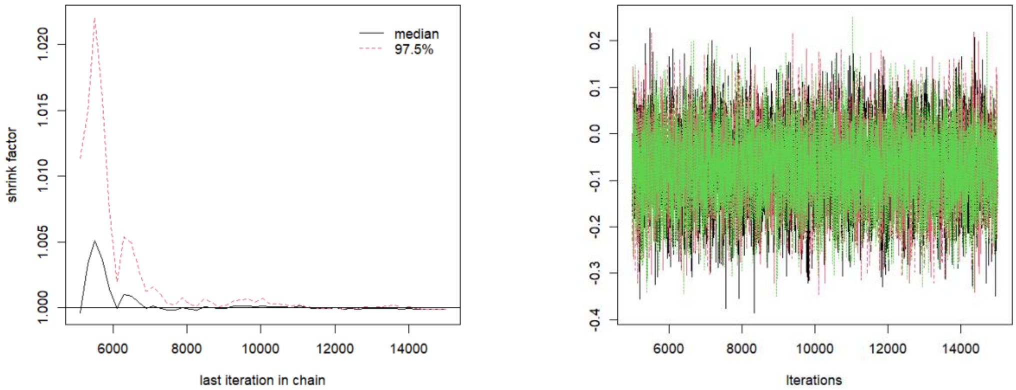

PES Quality foundations

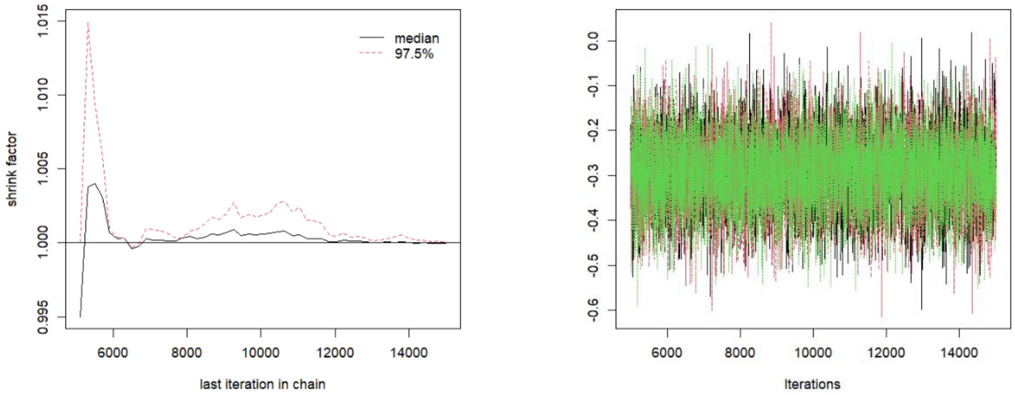

PES Staffing adequacy

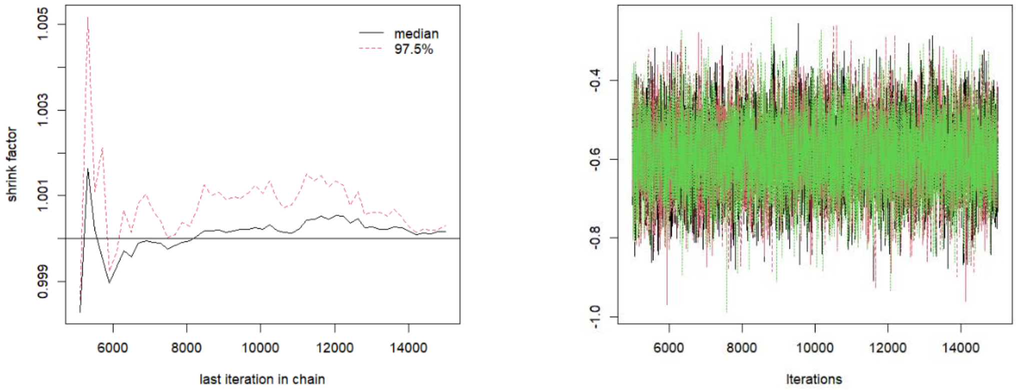

PES Manager ability

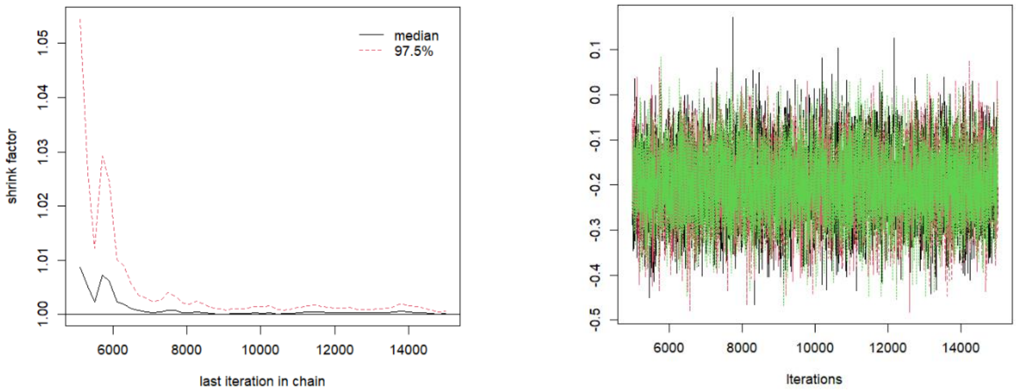

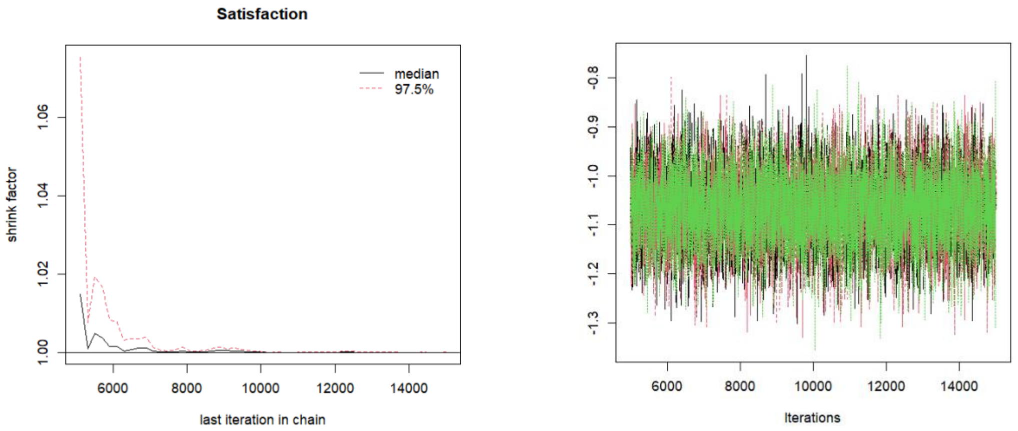
Satisfaction
median
97.5%
shrink factor
1.00
1.02
1.04
1.06
6000
8000
10000
12000
14000
last iteration in chain
-1.3
-1.2
-1.1
-1.0
-0.9
-0.8
Iterations

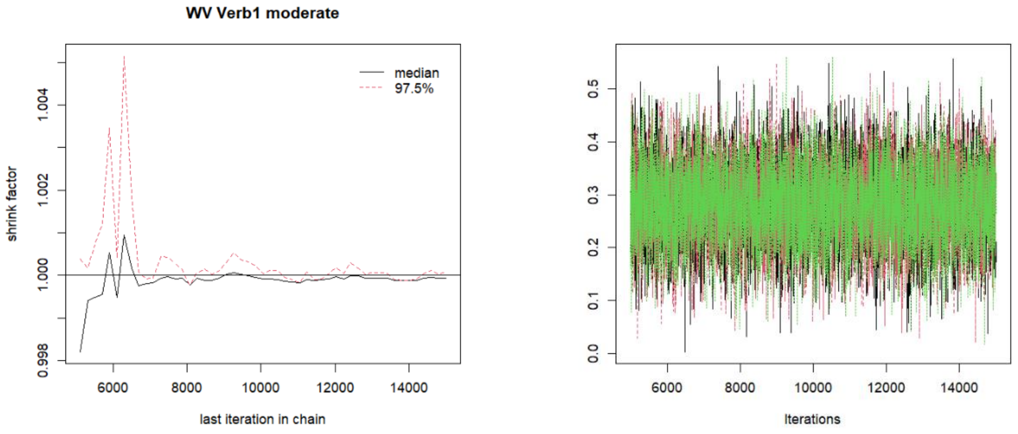
WV Verb1 moderate
median
97.5%
shrink factor
0.998
1.000
1.002
1.004
6000
8000
10000
12000
14000
last iteration in chain
0.0
0.1
0.2
0.3
0.4
0.5
Iterations

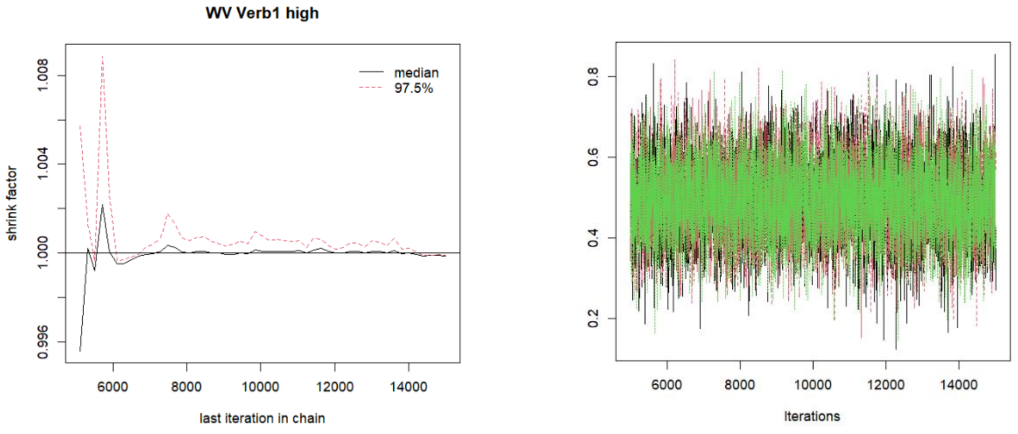
WV Verb1 high
median
97.5%
shrink factor
0.996
1.000
1.004
1.008
6000
8000
10000
12000
14000
last iteration in chain
0.2
0.4
0.6
0.8
Iterations

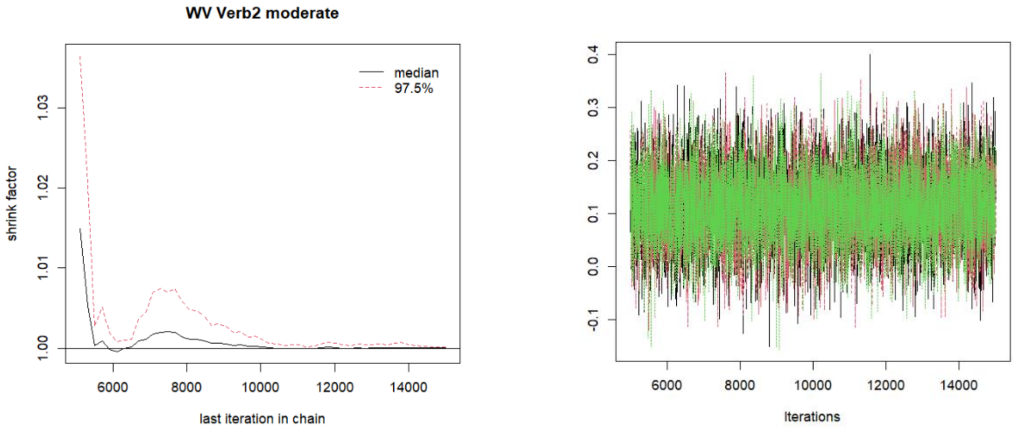
WV Verb2 moderate
median
97.5%
shrink factor
1.00
1.01
1.02
1.03
6000
8000
10000
12000
14000
last iteration in chain
-0.1
0.0
0.1
0.2
0.3
0.4
Iterations

WV Verb2 high

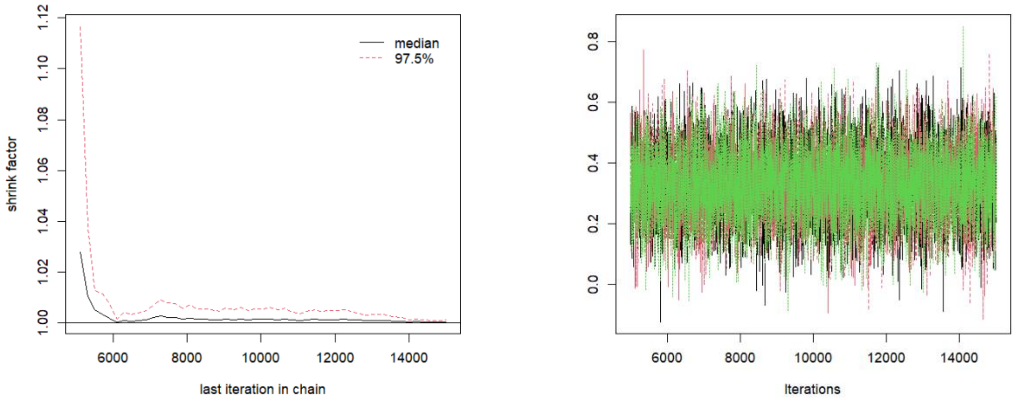

WV Phys1 moderate

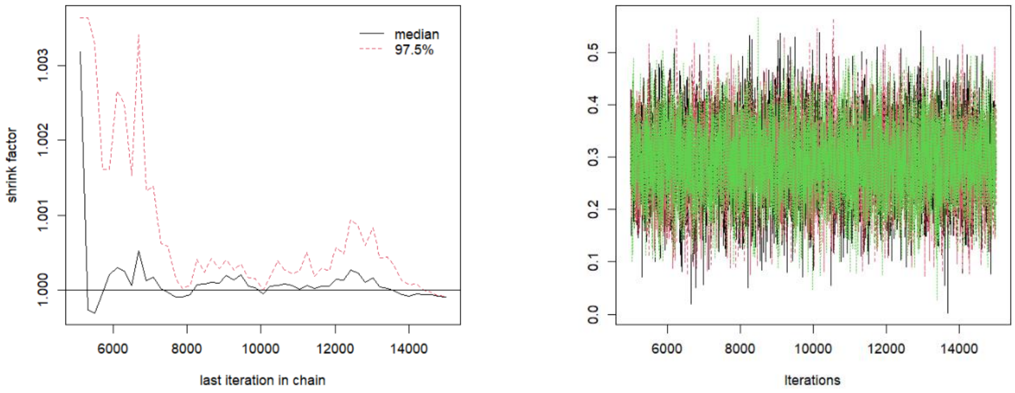

WV Phys1 high

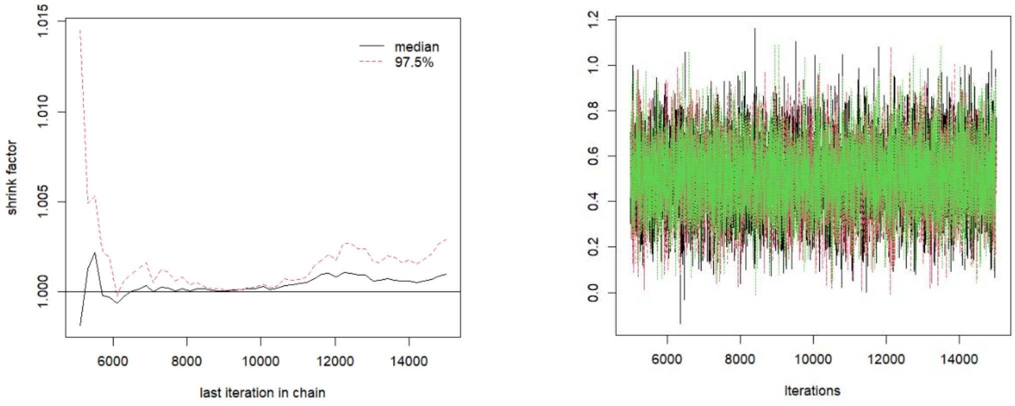

WV Phys2 high

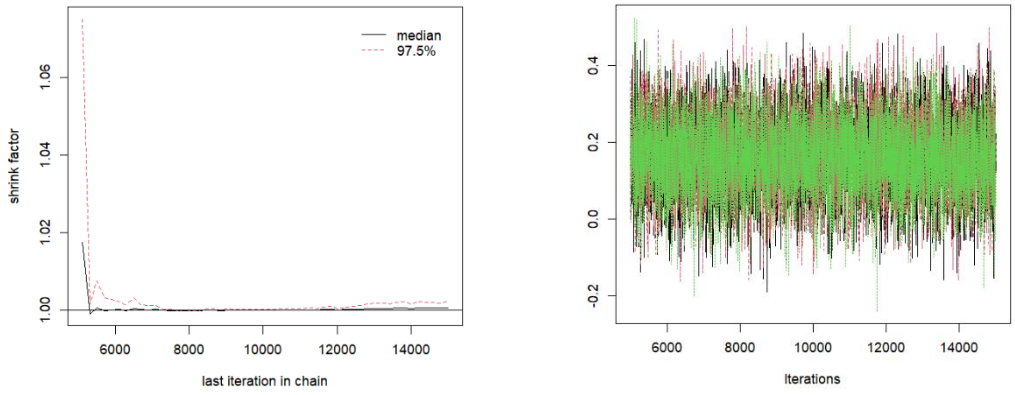

Supplement: Supplementary file 1 — Supplementary Material 1 [file 12912_2025_3745_MOESM1_ESM.pdf]
