# Supplementary material for: Impact of violence on emotional exhaustion risk of registered nurses in Germany: a Bayesian analysis of cross-sectional data with multiple imputations
Source: BMC Nurs. 2025 Aug 18;24:1080. doi: 10.1186/s12912-025-03745-y (PMC12359958; doi:10.1186/s12912-025-03745-y)
Supplement: Supplementary file 2 — Supplementary Material 2 [file 12912_2025_3745_MOESM2_ESM.pdf]

Appendix B – Literature searches

Variable selection - Literature-search

To identify relevant independent variables that explain MBI EE burnout in RNs, a comprehensive, but non-systematic literature search was conducted. 26 systematic reviews [1–26] were identified and screened for relevant articles. In addition, queries were conducted on scientific search engines (PubMed, Web of Science, ScienceDirect), using various search strings containing keywords such as - „Nurses, registered nurses, EMS, pre-clinical, pre-hospital, ambulance, paramedic, burnout, Maslach burnout inventory, violence, regression". Inclusion criteria were: Publication in a peer-reviewed journal; MBI compound or EE outcome/dependent variable; multivariable regression analysis; relevant population sufficiently present (RN or EMS workers); publication year after 2005 for RN and after 2000 for EMS workers; results and characteristics sufficiently reported. The difference in inclusion years resulted from lower publication volume of EMS-related articles. A total of 104 papers were included [7, 16, 27–128]. Characteristics such as sample size and population, regression models deployed, variable selection methods and burnout dimension (EE or compound MBI score) as well as the covariates included in regression models were tabulated and grouped into variable domains such as demographics, health, personality, and work-related variables, resulting in identification of 14 domains. Simple counts were used to estimate relative importance of the variable domain in the literature. Available variables were then matched to the relevant domains to select variables later included in the model. The results of the literature search and available selected variables can be seen in table 1.

<table>
<tr><th colspan="2">Variable domains from Literature</th><th>Variable in RN4Cast Data</th><th>Out of 104</th></tr>
<tr><td colspan="2">Demographics and Personal Background</td><td>Sex, seniority, academic degree, age</td><td>84</td></tr>
<tr><td colspan="2">Health</td><td>-</td><td>18</td></tr>
<tr><td colspan="3">Work Environment</td><td>77</td></tr>
<tr><td rowspan="5">Out of which:</td><td>Management, leadership and support</td><td>Nurse manager ability, leadership, and support of nurses*; Nurse-physician relations*</td><td>41</td></tr>
<tr><td>Working hours and shifts</td><td>Employment</td><td>31</td></tr>
<tr><td>Workload and staffing</td><td>Staffing and resource adequacy*</td><td>60</td></tr>
<tr><td>Professional quality and profession-related aspects</td><td>Foundation of quality of care*</td><td>15</td></tr>
<tr><td>Job control, organizational affairs and participation</td><td>Participation in hospital affairs*</td><td>29</td></tr>
<tr><td colspan="2">Personality-related</td><td>-</td><td>21</td></tr>
<tr><td colspan="2">Structural aspects</td><td>-</td><td>33</td></tr>
<tr><td colspan="2">Violence, aggression, and conflicts</td><td>Violence frequency</td><td>21</td></tr>
<tr><td colspan="2">Financial aspects</td><td>Satisfaction with wage</td><td>8</td></tr>
<tr><td colspan="2">Work satisfaction</td><td>Work satisfaction</td><td>14</td></tr>
<tr><td colspan="2">Other</td><td>-</td><td>24</td></tr>
</table>

Table B1 - Results from literature search and variable selection
Note: * = Items or questions from PES-NWI instruments

It is noteworthy that in the included literature, regardless of scoring key, analyses were run on dichotomized outcome of "High EE" vs. lower categories.

Violence variables - literature search

In addition to the covariates selected explained in the prior section, special attention is given to the operationalization of the violence measuring variables. This section aims to address two central aspects to evaluate comparability and justify the violence-related variables used:

1) How is the experience of violence measured in the relevant healthcare-workplace context?

2) Which aspects of the measured violence experience are relevant to (EE) burnout?

1) Measuring and operationalizing violence entails a multitude of challenges such as divergent definitions and research questions, that may inform the context of violence being studied (e.g. work-related violence, criminal violence, or domestic violence). Different study designs, data sources and sampling methods also extend to various approaches, such as victim surveys versus registry or insurance and claims data.

There are several recommendations for research on violence, workplace violence or violence against women [129–132]. These works recommend careful consideration of certain aspects of violence to adapt and integrate into the research question, including definitions of violence, mechanisms of violence, surveying techniques, cultural contexts, and more. For the given research question, four key aspects were chosen to evaluate variables present in the literature and subsequently, in the data available:

- **Types of violence:** The different forms that violence can take.
- **Perpetrator Relationship:** The relationship between victim and perpetrator.
- **Timeframe:** Framing of the timespan in which violence is surveyed
- **Measurement:** The statistical method and scale with which violence is measured

To answer question 1, these four aspects were evaluated through a literature search on survey-based research related to violence directed towards healthcare workers.
Citations from 20 systematic reviews [3–7, 10, 12, 14, 16–21, 24, 25, 133–136] and queries on scientific search engines (PubMed, Web of Science, ScienceDirect) were used to screen the literature. Inclusion criteria were: Publication in a peer-reviewed journal; publication year 2000 or more recent; empirical cross-sectional design; survey-based measure of violence present; context of EMS, hospitals or fire-stations and population of nurses, EMS or other non-physician workers such as medical assistants. Exclusion criteria were: Qualitative designs; overrepresented physician populations; context such as nursing homes, criminal or correctional facilities, ambulatory practices, military or defense-installations, active conflict zones and Covid-19 related violence. A total of 74 publications were included [30, 32, 43, 58–60, 62, 63, 80, 81, 106, 119, 120, 137–197]. Results for each of the four relevant aspects are described in the following:

While 45.5% of papers did not frame any person or group as the surveyed for perpetrator, the majority (54.5%) specified patients as the main perpetrators in their surveys. In addition to the patient category, violence from family/visitors and staff/colleagues was also commonly specified. Other categories included bystanders, the public or other specific people. The two most common types of violence were physical and verbal, which were addressed jointly in most papers (92%). Some papers further fragmented physical and/or verbal violence into specific acts in their questionnaires (13%), such as spitting, biting, shouting or use of weapons. Another notable and common type was sexual violence, which was exclusively surveyed alongside physical and verbal violence. Similarly, threats (both verbal and physical) were surveyed for as more specific but not distinct subsets of violence. Other less frequently used specifications of violence included but were not limited to intimidation (verbal and physical), violence towards materials and objects, bullying and weapon-based violence.

While a few papers did not report the timeframe in which respondents were asked to report experiences of violence (9%), most studies used a timeframe of 12 months, by either implicitly or explicitly stated in their surveys (62% of those who reported a timeframe). Explicit formulation in this case refers to surveys which introduced a timeframe a priori, while implicit means that only the survey items spanned up to a certain period.

Statistical measurement of violence was most prominent as a type of frequency measurement in the surveyed for timespan (49%), usually employing Likert-type scales. However, some papers used a lower resolution binary measurement in the form of any occurrence or prevalence of violence within that period of time (26%). Frequency and occasionally, occurrence of violence were paired with a measure of violence severity in a few cases. Furthermore, some papers featured measures other than direct, self-reported experience of violence, such as fear of violence, suspected reason for violence or the description of the most violent episodes to memory or in a certain timeframe.

To summarize, the literature on workplace-violence towards nurses and EMS workers typically surveys physical, verbal, and sexual violence perpetrated by patients, family members, and colleagues. Most often, a period covering the last 12 months is used, with questions measured by respondents reporting the frequency of the violence they experienced. This time period is deemed sufficient to observe changes in burnout symptoms influenced by WV, as evidenced by a prospective study [198], albeit using a population of social educators and measuring burnout with the Copenhagen Burnout Scale. There might be a meaningful distinction that concerns wording of the questions regarding violence experienced, which could frame violence towards the respondent alone or more generally, any violence witnessed at the workplace. This distinction was sometimes made explicitly in surveys. However, due to unclear or missing reporting and justification, no clear trend is recognizable

While in nursing, most papers used some form of validated questionnaire to measure violence (59%), papers studying EMS workers more frequently used self-designed instruments or developed variations of other validated instruments (55.5%). The screening included both studies limited to description of violence as well as analyses of the influence of violence on different outcomes, most frequently: burnout; post-traumatic stress disorder (PTSD); composite outcomes of mental health instruments and intention to leave the workplace or profession.

2) Based on the literature search performed in section 1), the question arises whether PTSD is a proximal contender of mental health conditions that may be determined by violence, since this mental health condition was the most commonly differentiated and defined condition found. This leads to the consideration of 2): how and whether certain aspects of experienced violence might specifically affect PTSD or burnout and whether the variables in the analysis reflect sufficiently the specific characteristics of violence experience that are connected to burnout.

Violence in its various forms is an exposure connected to PTSD via traumatic experience by virtue of intrinsic definition of the condition [199–202]. In the context of healthcare, causes of PTSD include unpredictable events such as sudden death, dealing with critical trauma cases, confrontation with great potential for violent behavior, and sexual harassment [201, 203, 204]. Severe psychological trauma, a high perceived threat to life for oneself or others, cumulative exposure to violence and severe or injurious assault are considered peri-traumatic factors [202, 205], further linking traumatic events to the risk of PTSD according to the theoretical model of trauma [206]. In contrast, exposure to WV is connected to the risk of MBI burnout dimensions by depleting nurses energy and activation of a negative loss cycle [135, 207]. This aligns with other studies [208], subsuming workplace violence under a type of job demand based on the job demands-resources model [209, 210], which erodes resources over time. Constant high demands in the form of facing and dealing with frequent violence fit within the mechanisms of burnout development, described as persistent imbalance of demands over resources [15, 16, 37, 211–215]. Violence frequency was generally shown to be associated with both diseases in research [135, 202, 216]. Only one publication differentiates the effects of frequency and severity on PTSD, notably finding that exposure to severe WV is similarly detrimental to risk of PTSD as the highest frequency exposure to WV in a sample of social educators [216]. Since evidence suggests that PTSD symptoms are linked to experiences of re-living or revictimization [135, 146, 199, 200, 217, 218], this teases the hypothesis that frequency of violence and the encounter of at least one traumatic experience (which caused the PTSD) or a re-living event are correlated to a certain degree, which makes discriminating between these associations complex. This is reinforced by evidence that burnout and PTSD themselves are associated [143, 202, 219]. However, the etiological development of burnout and literature search in section 2.4 supports the view that violence frequency is more relevant for the development of burnout than violence severity, which in terms is more connected to PTSD. This is also supported by literature describing differential impact of frequency of traumatic

experiences on PTSD, finding that the frequency of early life events causes more severe PTSD symptoms compared to events occurring more frequently later in life [220] and that trauma count is secondary to early trauma age concerning PTSD symptom treatment and magnitude [221]. To conclude, the variables present in the data do not cover the frequency of sexual assault but reflect high comparability with

References to appendix B

1. Thielmann B, Schwarze R, Böckelmann I (2023) A Systematic Review of Associations and Predictors for Job Satisfaction and Work Engagement in Prehospital Emergency Medical Services—Challenges for the Future. IJERPH 20(5), 4578. 10.3390/ijerph20054578.
2. Sahebi A, Golitaleb M, Jahangiri K (2021) Occupational Burnout in Pre-Hospital Emergency Personnel in Iran: A Systematic Review and Meta-Analysis. Iranian Journal of Nursing and Midwifery Research 26(1), 11–17. 10.4103/ijnmr.IJNMR_175_20.
3. Shabanikiya H, Kokabisaghi F, Mojtabaeian M, Sahebi T, Varmaghani M (2021) Global Prevalence of Workplace Violence Against Paramedics: A Systematic Review and Meta-analysis. HDQ 6(4), 205–216. 10.32598/hdq.6.4.259.3.
4. Murray RM, Davis AL, Shepler LJ, Moore-Merrell L, Troup WJ, Allen JA, Taylor JA (2020) A Systematic Review of Workplace Violence Against Emergency Medical Services Responders. New solutions a journal of environmental and occupational health policy NS 29(4), 487–503. 10.1177/1048291119893388.
5. Reardon M, Abrahams R, Thyer L, Simpson P (2020) Review article: Prevalence of burnout in paramedics: A systematic review of prevalence studies. Emerg Medicine Australasia 32(2), 182–189. 10.1111/1742-6723.13478.
6. Maguire BJ, O'Meara P, O'Neill BJ, Brightwell R (2018) Violence against emergency medical services personnel: A systematic review of the literature. American journal of industrial medicine 61(2), 167–180. 10.1002/ajim.22797.
7. Sterud T, Ekeberg Ø, Hem E (2006) Health status in the ambulance services: a systematic review. BMC health services research 6, 82. 10.1186/1472-6963-6-82.
8. Papazian L, Hraiech S, Loundou A, Herridge MS, Boyer L (2023) High-level burnout in physicians and nurses working in adult ICUs: a systematic review and meta-analysis. Intensive care medicine 49(4), 387–400. 10.1007/s00134-023-07025-8.
9. Tamata AT, Mohammadnezhad M (2023) A systematic review study on the factors affecting shortage of nursing workforce in the hospitals. Nursing open 10(3), 1247–1257. 10.1002/nop2.1434.
10. Chakraborty S, Mashreky SR, Dalal K (2022) Violence against physicians and nurses: a systematic literature review. J Public Health 30(8), 1837–1855. 10.1007/s10389-021-01689-6.
11. Niinihuhta M, Häggman-Laitila A (2022) A systematic review of the relationships between nurse leaders' leadership styles and nurses' work-related well-being. International journal of nursing practice 28(5), e13040. 10.1111/ijn.13040.
12. Zhang Y, Cai J, Yin R, Qin S, Wang H, Shi X, Mao L (2022) Prevalence of lateral violence in nurse workplace: a systematic review and meta-analysis. BMJ open 12(3), e054014. 10.1136/bmjopen-2021-054014.
13. Galanis P, Vraka I, Fragkou D, Bilali A, Kaitelidou D (2021) Nurses' burnout and associated risk factors during the COVID-19 pandemic: A systematic review and meta-analysis. Journal of advanced nursing 77(8), 3286–3302. 10.1111/jan.14839.
14. Giménez Lozano JM, Martínez Ramón JP, Morales Rodríguez FM (2021) Doctors and Nurses: A Systematic Review of the Risk and Protective Factors in Workplace Violence and Burnout. International journal of environmental research and public health 18(6). 10.3390/ijerph18063280.
15. Ramírez-Elvira S, Romero-Béjar JL, Suleiman-Martos N, Gómez-Urquiza JL, Monsalve-Reyes C, La Cañadas-De Fuente GA, Albendín-García L (2021) Prevalence, Risk Factors and Burnout Levels in Intensive Care Unit Nurses: A Systematic Review and Meta-Analysis. International journal of environmental research and public health 18(21). 10.3390/ijerph182111432.
16. Dall'Ora C, Ball J, Reinius M, Griffiths P (2020) Burnout in nursing: a theoretical review. Human resources for health 18(1), 41. 10.1186/s12960-020-00469-9.
17. Pariona-Cabrera P, Cavanagh J, Bartram T (2020) Workplace violence against nurses in health care and the role of human resource management: A systematic review of the literature. Journal of advanced nursing 76(7), 1581–1593. 10.1111/jan.14352.

18. Woo T, Ho R, Tang A, Tam W (2020) Global prevalence of burnout symptoms among nurses: A systematic review and meta-analysis. Journal of psychiatric research 123, 9–20. 10.1016/j.jpsychires.2019.12.015.
19. Crawford CL, Chu F, Judson LH, Cuenca E, Jadalla AA, Tze-Polo L, Kawar LN, Runnels C, Garvida R (2019) An Integrative Review of Nurse-to-Nurse Incivility, Hostility, and Workplace Violence: A GPS for Nurse Leaders. Nursing administration quarterly 43(2), 138–156. 10.1097/NAQ.0000000000000338.
20. López-López IM, Gómez-Urquiza JL, Cañadas GR, La Fuente EI de, Albendín-García L, La Cañadas-De Fuente GA (2019) Prevalence of burnout in mental health nurses and related factors: a systematic review and meta-analysis. International journal of mental health nursing 28(5), 1032–1041. 10.1111/inm.12606.
21. Molina-Praena J, Ramirez-Baena L, Gómez-Urquiza JL, Cañadas GR, La Fuente EI de, La Cañadas-De Fuente GA (2018) Levels of Burnout and Risk Factors in Medical Area Nurses: A Meta-Analytic Study. International journal of environmental research and public health 15(12). 10.3390/ijerph15122800.
22. Shin S, Park J-H, Bae S-H (2018) Nurse staffing and nurse outcomes: A systematic review and meta-analysis. Nursing outlook 66(3), 273–282. 10.1016/j.outlook.2017.12.002.
23. Chuang C-H, Tseng P-C, Lin C-Y, Lin K-H, Chen Y-Y (2016) Burnout in the intensive care unit professionals: A systematic review. Medicine 95(50), e5629. 10.1097/MD.0000000000005629.
24. Adriaenssens J, Gucht V de, Maes S (2015) Determinants and prevalence of burnout in emergency nurses: a systematic review of 25 years of research. International journal of nursing studies 52(2), 649–661. 10.1016/j.ijnurstu.2014.11.004.
25. Lanctôt N, Guay S (2014) The aftermath of workplace violence among healthcare workers: A systematic literature review of the consequences. Aggression and Violent Behavior 19(5), 492–501. 10.1016/j.avb.2014.07.010.
26. Khamisa N, Peltzer K, Oldenburg B (2013) Burnout in relation to specific contributing factors and health outcomes among nurses: a systematic review. International journal of environmental research and public health 10(6), 2214–2240. 10.3390/ijerph10062214.
27. Boland LL, Kinzy TG, Myers RN, Fernstrom KM, Kamrud JW, Mink PJ, Stevens AC (2018) Burnout and Exposure to Critical Incidents in a Cohort of Emergency Medical Services Workers from Minnesota. The western journal of emergency medicine 19(6), 987–995. 10.5811/westjem.8.39034.
28. Boland LL, Mink PJ, Kamrud JW, Jeruzal JN, Stevens AC (2019) Social Support Outside the Workplace, Coping Styles, and Burnout in a Cohort of EMS Providers From Minnesota. Workplace health & safety 67(8), 414–422. 10.1177/2165079919829154.
29. Haruna J, Uemura S, Taguchi Y, Muranaka S, Niiyama S, Inamura H, Sawamoto K, Mizuno H, Narimatsu E (2023) Influence of work and family environment on burnout among emergency medical technicians. Clin Exp Emerg Med 10(3), 287–295. 10.15441/ceem.22.389.
30. Kang J-H, Sakong J, Kim JH (2022) Impact of violence on the burnout status of paramedics in the emergency department: A multicenter survey study. Australasian emergency care 25(2), 147–153. 10.1016/j.auec.2021.07.002.
31. van der Ploeg E, Kleber RJ (2003) Acute and chronic job stressors among ambulance personnel: predictors of health symptoms. Occupational and environmental medicine 60 Suppl 1(Suppl 1), i40-6. 10.1136/oem.60.suppl_1.i40.
32. Hensel JM, Lunsky Y, Dewa CS (2015) Exposure to aggressive behaviour and burnout in direct support providers: The role of positive work factors. Research in developmental disabilities 36C, 404–412. 10.1016/j.ridd.2014.10.033.
33. Moukarzel A, Michelet P, Durand A-C, Sebbane M, Bourgeois S, Markarian T, Bompard C, Gentile S (2019) Burnout Syndrome among Emergency Department Staff: Prevalence and Associated Factors. BioMed research international 2019, 6462472. 10.1155/2019/6462472.

34. Chirico F, Crescenzo P, Sacco A, Riccò M, Ripa S, Nucera G, Magnavita N (2021) Prevalence of burnout syndrome among Italian volunteers of the Red Cross: a cross-sectional study. Industrial health 59(2), 117–127. 10.2486/indhealth.2020-0246.
35. Tarchi L, Crescenzo P, Talamonti K (2023) Prevalence and predictors of mental distress among Italian Red Cross auxiliary corps: A cross-sectional evaluation after deployment in anti-COVID-19 operations. Military psychology the official journal of the Division of Military Psychology, American Psychological Association 35(5), 394–407. 10.1080/08995605.2022.2069983.
36. Vagni M, Maiorano T, Giostra V, Pajardi D, Bartone P (2022) Emergency Stress, Hardiness, Coping Strategies and Burnout in Health Care and Emergency Response Workers During the COVID-19 Pandemic. Front. Psychol. 13. 10.3389/fpsyg.2022.918788.
37. Sporer C (2021) Burnout in emergency medical technicians and paramedics in the USA. IJES 10(3), 366–389. 10.1108/IJES-03-2020-0012.
38. ALmutairi MN, El Mahalli AA (2020) Burnout and Coping Methods among Emergency Medical Services Professionals. Journal of multidisciplinary healthcare 13, 271–279. 10.2147/JMDH.S244303.
39. Haji Mohammad Hoseini M, Ghanbari Afra L, Aliakbarzade Arani Z, Abdi M (2017) Mental Health and Job Burnout Among Pre-Hospital Emergency Care Personnel. HDQ 2(2), 89–94. 10.18869/nrip.hdq.2.2.89.
40. Akram Z, Li Y, Akram U (2019) When Employees are Emotionally Exhausted Due to Abusive Supervision. A Conservation-of-Resources Perspective. International journal of environmental research and public health 16(18). 10.3390/ijerph16183300.
41. Babenko-Mould Y, Laschinger HKS (2014) Effects of incivility in clinical practice settings on nursing student burnout. International journal of nursing education scholarship 11. 10.1515/ijnes-2014-0023.
42. García-Izquierdo M, Ríos-Rísquez MI (2012) The relationship between psychosocial job stress and burnout in emergency departments: an exploratory study. Nursing outlook 60(5), 322–329. 10.1016/j.outlook.2012.02.002.
43. Galián-Muñoz I, Ruiz-Hernández JA, Llor-Esteban B, López-García C (2016) User Violence and Nursing Staff Burnout: The Modulating Role of Job Satisfaction. Journal of interpersonal violence 31(2), 302–315. 10.1177/0886260514555367.
44. Chen S, Lin S, Ruan Q, Li H, Wu S (2016) Workplace violence and its effect on burnout and turnover attempt among Chinese medical staff. Archives of environmental & occupational health 71(6), 330–337. 10.1080/19338244.2015.1128874.
45. Andela M, Truchot D, van der Doef M (2016) Job stressors and burnout in hospitals: The mediating role of emotional dissonance. International Journal of Stress Management 23(3), 298–317. 10.1037/str0000013.
46. Bagheri Hosseinabadi M, Ebrahimi MH, Khanjani N, Biganeh J, Mohammadi S, Abdolahfard M (2019) The effects of amplitude and stability of circadian rhythm and occupational stress on burnout syndrome and job dissatisfaction among irregular shift working nurses. Journal of clinical nursing 28(9-10), 1868–1878. 10.1111/jocn.14778.
47. Willis TA, O'Connor DB, Smith L (2008) Investigating effort–reward imbalance and work–family conflict in relation to morningness–eveningness and shift work. Work & Stress 22(2), 125–137. 10.1080/02678370802180558.
48. Rezaei O, Habibi K, Arab Ghahestany D, Sayadnasiri M, Armoon B, Khan V, Fattah Moghadam L (2018) Factors related to job burnout among nurses in the Razi Psychiatric Hospital, Iran. International journal of adolescent medicine and health 32(3). 10.1515/ijamh-2017-0146.
49. Cao X, Naruse T (2019) Effect of time pressure on the burnout of home-visiting nurses: The moderating role of relational coordination with nursing managers. Japan journal of nursing science JJNS 16(2), 221–231. 10.1111/jjns.12233.
50. van der Doef M, Mbazzi FB, Verhoeven C (2012) Job conditions, job satisfaction, somatic complaints and burnout among East African nurses. Journal of clinical nursing 21(11-12), 1763–1775. 10.1111/j.1365-2702.2011.03995.x.

51. Myhren H, Ekeberg O, Stokland O (2013) Job Satisfaction and Burnout among Intensive Care Unit Nurses and Physicians. Critical care research and practice 2013, 786176. 10.1155/2013/786176.
52. Ayala E, Carnero AM (2013) Determinants of burnout in acute and critical care military nursing personnel: a cross-sectional study from Peru. PloS one 8(1), e54408. 10.1371/journal.pone.0054408.
53. Aydin Sayilan A, Kulakaç N, Uzun S (2021) Burnout levels and sleep quality of COVID-19 heroes. Perspectives in psychiatric care 57(3), 1231–1236. 10.1111/ppc.12678.
54. Stone PW, Du Y, Gershon RRM (2007) Organizational climate and occupational health outcomes in hospital nurses. Journal of occupational and environmental medicine 49(1), 50–58. 10.1097/01.jom.0000251622.05429.0c.
55. Shao J, Tang L, Wang X, Qiu R, Zhang Y, Jia Y, Ma Y, Ye Z (2018) Nursing work environment, value congruence and their relationships with nurses' work outcomes. Journal of nursing management 26(8), 1091–1099. 10.1111/jonm.12641.
56. Dhaini SR, Denhaerynck K, Bachnick S, Schwendimann R, Schubert M, Geest S de, Simon M (2018) Work schedule flexibility is associated with emotional exhaustion among registered nurses in Swiss hospitals: A cross-sectional study. International journal of nursing studies 82, 99–105. 10.1016/j.ijnurstu.2018.03.019.
57. La Cañadas-De Fuente GA, Vargas C, San Luis C, García I, Cañadas GR, La Fuente EI de (2015) Risk factors and prevalence of burnout syndrome in the nursing profession. International journal of nursing studies 52(1), 240–249. 10.1016/j.ijnurstu.2014.07.001.
58. Merecz D, Drabek M, Mościcka A (2009) Aggression at the workplace--psychological consequences of abusive encounter with coworkers and clients. International journal of occupational medicine and environmental health 22(3), 243–260. 10.2478/v10001-009-0027-2.
59. Pai DD, Lautert L, Souza SBC de, Marziale MHP, Tavares JP (2015) Violência, burnout e transtornos psíquicos menores no trabalho hospitalar. Revista da Escola de Enfermagem da U S P 49(3), 460–468. 10.1590/S0080-623420150000300014.
60. Hamaideh SH (2011) Burnout, social support, and job satisfaction among Jordanian mental health nurses. Issues in mental health nursing 32(4), 234–242. 10.3109/01612840.2010.546494.
61. Padilla Fortunatti C, Palmeiro-Silva YK (2017) Effort-Reward Imbalance and Burnout Among ICU Nursing Staff: A Cross-Sectional Study. Nursing research 66(5), 410–416. 10.1097/NNR.0000000000000239.
62. Vincent-Höper S, Stein M, Nienhaus A, Schablon A (2020) Workplace Aggression and Burnout in Nursing-The Moderating Role of Follow-Up Counseling. International journal of environmental research and public health 17(9). 10.3390/ijerph17093152.
63. Goussinsky R, Livne Y (2019) The Role of Coping Resources in the Relationship Between Mistreatment and Job Burnout: Evidence Across Two Healthcare Settings. Journal of Aggression, Maltreatment & Trauma 28(10), 1250–1268. 10.1080/10926771.2018.1473905.
64. McTiernan K, McDonald N (2015) Occupational stressors, burnout and coping strategies between hospital and community psychiatric nurses in a Dublin region. Journal of psychiatric and mental health nursing 22(3), 208–218. 10.1111/jpm.12170.
65. Konstantinou A-K, Bonotis K, Sokratous M, Siokas V, Dardiotis E (2018) Burnout Evaluation and Potential Predictors in a Greek Cohort of Mental Health Nurses. Archives of psychiatric nursing 32(3), 449–456. 10.1016/j.apnu.2018.01.002.
66. Kanste O, Kyngäs H, Nikkilä J (2007) The relationship between multidimensional leadership and burnout among nursing staff. Journal of nursing management 15(7), 731–739. 10.1111/j.1365-2934.2006.00741.x.
67. Ohue T, Moriyama M, Nakaya T (2011) Examination of a cognitive model of stress, burnout, and intention to resign for Japanese nurses. Japan journal of nursing science JJNS 8(1), 76–86. 10.1111/j.1742-7924.2010.00161.x.
68. van Bogaert P, Clarke S, Vermeyen K, Meulemans H, van de Heyning P (2009) Practice environments and their associations with nurse-reported outcomes in Belgian hospitals:

development and preliminary validation of a Dutch adaptation of the Revised Nursing Work Index. International journal of nursing studies 46(1), 54–64. 10.1016/j.ijnurstu.2008.07.009.
69. Rizo-Baeza M, Mendiola-Infante SV, Sepehri A, Palazón-Bru A, Gil-Guillén VF, Cortés-Castell E (2018) Burnout syndrome in nurses working in palliative care units: An analysis of associated factors. Journal of nursing management 26(1), 19–25. 10.1111/jonm.12506.
70. Petrino R, Riesgo LG-C, Yilmaz B (2022) Burnout in emergency medicine professionals after 2 years of the COVID-19 pandemic: a threat to the healthcare system? European journal of emergency medicine official journal of the European Society for Emergency Medicine 29(4), 279–284. 10.1097/MEJ.0000000000000952.
71. Aiken LH, Clarke SP, Sloane DM, Lake ET, Cheney T (2008) Effects of hospital care environment on patient mortality and nurse outcomes. The Journal of nursing administration 38(5), 223–229. 10.1097/01.NNA.0000312773.42352.d7.
72. McHugh MD, Ma C (2014) Wage, work environment, and staffing: effects on nurse outcomes. Policy, politics & nursing practice 15(3-4), 72–80. 10.1177/1527154414546868.
73. Cho S-H, June KJ, Kim YM, Cho YA, Yoo CS, Yun S-C, Sung YH (2009) Nurse staffing, quality of nursing care and nurse job outcomes in intensive care units. Journal of clinical nursing 18(12), 1729–1737. 10.1111/j.1365-2702.2008.02721.x.
74. Teixeira C, Ribeiro O, Fonseca AM, Carvalho AS (2013) Burnout in intensive care units - a consideration of the possible prevalence and frequency of new risk factors: a descriptive correlational multicentre study. BMC anesthesiology 13(1), 38. 10.1186/1471-2253-13-38.
75. Wisetborisut A, Angkurawaranon C, Jiraporncharoen W, Uaphanthasath R, Wiwatanadate P (2014) Shift work and burnout among health care workers. Occupational medicine (Oxford, England) 64(4), 279–286. 10.1093/occmed/kqu009.
76. Li B, Bruyneel L, Sermeus W, van den Heede K, Matawie K, Aiken L, Lesaffre E (2013) Group-level impact of work environment dimensions on burnout experiences among nurses: a multivariate multilevel probit model. International journal of nursing studies 50(2), 281–291. 10.1016/j.ijnurstu.2012.07.001.
77. Nikpour J, Carthon JMB (2023) Characteristics, work environments, and rates of burnout and job dissatisfaction among registered nurses in primary care. Nursing outlook 71(4), 101988. 10.1016/j.outlook.2023.101988.
78. Patrician PA, Shang J, Lake ET (2010) Organizational determinants of work outcomes and quality care ratings among Army Medical Department registered nurses. Research in nursing & health 33(2), 99–110. 10.1002/nur.20370.
79. Bruyneel A, Smith P, Tack J, Pirson M (2021) Prevalence of burnout risk and factors associated with burnout risk among ICU nurses during the COVID-19 outbreak in French speaking Belgium. Intensive & critical care nursing 65, 103059. 10.1016/j.iccn.2021.103059.
80. Anwar MM, Elareed HR (2017) Burnout among Egyptian Nurses. J Public Health 25(6), 693–697. 10.1007/s10389-017-0831-2.
81. Estryn-Behar M, van der Heijden B, Camerino D, Fry C, Le Nezet O, Conway PM, Hasselhorn H-M (2008) Violence risks in nursing--results from the European 'NEXT' Study. Occupational medicine (Oxford, England) 58(2), 107–114. 10.1093/occmed/kqm142.
82. Verdon M, Merlani P, Perneger T, Ricou B (2008) Burnout in a surgical ICU team. Intensive care medicine 34(1), 152–156. 10.1007/s00134-007-0907-5.
83. Hamdan M, Hamra AA (2017) Burnout among workers in emergency Departments in Palestinian hospitals: prevalence and associated factors. BMC health services research 17(1), 407. 10.1186/s12913-017-2356-3.
84. Poncet MC, Toullic P, Papazian L, Kentish-Barnes N, Timsit J-F, Pochard F, Chevret S, Schlemmer B, Azoulay E (2007) Burnout syndrome in critical care nursing staff. American journal of respiratory and critical care medicine 175(7), 698–704. 10.1164/rccm.200606-806OC.
85. Flynn L, Thomas-Hawkins C, Clarke SP (2009) Organizational traits, care processes, and burnout among chronic hemodialysis nurses. Western journal of nursing research 31(5), 569–582. 10.1177/0193945909331430.

86. Liu K, You L-M, Chen S-X, Hao Y-T, Zhu X-W, Zhang L-F, Aiken LH (2012) The relationship between hospital work environment and nurse outcomes in Guangdong, China: a nurse questionnaire survey. Journal of clinical nursing 21(9-10), 1476–1485. 10.1111/j.1365-2702.2011.03991.x.
87. Zhou W, He G, Wang H, He Y, Yuan Q, Liu D (2015) Job dissatisfaction and burnout of nurses in Hunan, China: A cross-sectional survey. Nursing & health sciences 17(4), 444–450. 10.1111/nhs.12213.
88. Lu M, Ruan H, Xing W, Hu Y (2015) Nurse burnout in China: a questionnaire survey on staffing, job satisfaction, and quality of care. Journal of nursing management 23(4), 440–447. 10.1111/jonm.12150.
89. See KC, Zhao MY, Nakataki E, Chittawatanarat K, Fang W-F, Faruq MO, Wahjuprajitno B, Arabi YM, Wong WT, Divatia JV, Palo JE, Shrestha BR, Nafees KMK, Binh NG, Al Rahma HN, Detleuxay K, Ong V, Phua J (2018) Professional burnout among physicians and nurses in Asian intensive care units: a multinational survey. Intensive care medicine 44(12), 2079–2090. 10.1007/s00134-018-5432-1.
90. Matsuo T, Kobayashi D, Taki F, Sakamoto F, Uehara Y, Mori N, Fukui T (2020) Prevalence of Health Care Worker Burnout During the Coronavirus Disease 2019 (COVID-19) Pandemic in Japan. JAMA network open 3(8), e2017271. 10.1001/jamanetworkopen.2020.17271.
91. Hu Z, Wang H, Xie J, Zhang J, Li H, Liu S, Li Q, Yang Y, Huang Y (2021) Burnout in ICU doctors and nurses in mainland China-A national cross-sectional study. Journal of critical care 62, 265–270. 10.1016/j.jcrc.2020.12.029.
92. Suzuki E, Saito M, Tagaya A, Mihara R, Maruyama A, Azuma T, Sato C (2009) Relationship between assertiveness and burnout among nurse managers. Japan Journal Nursing Sci 6(2), 71–81. 10.1111/j.1742-7924.2009.00124.x.
93. Yuan L, Yumeng C, Chunfen Z, Jinbo F (2020) Analyzing the Impact of Practice Environment on Nurse Burnout Using Conventional and Multilevel Logistic Regression Models. Workplace health & safety 68(7), 325–336. 10.1177/2165079919900796.
94. Merces MCd, Coelho JMF, Lua I, Silva DdSe, Gomes AMT, Erdmann AL, Oliveira DC de, Lago SB, Santana AIC, Da Silva DAR, Servo MLS, Sobrinho CLN, Marques SC, Figueiredo VP, Peres EM, Souza MC de, França LCM, Maciel DMC, Peixoto ÁRS, Couto PLS, Maia MdS, Marinho MCG, França SLG, Guimarães CF, Santos KA, Barreto FL, Castro JdO, Santos MPdS, Coutinho MO, Passos KG, Tosta Maciel RRB, Camelier FWR, Júnior AD (2020) Prevalence and Factors Associated with Burnout Syndrome among Primary Health Care Nursing Professionals: A Cross-Sectional Study †. International journal of environmental research and public health 17(2). 10.3390/ijerph17020474.
95. Lopes AR, Nihei OK (2020) Burnout among nursing students: predictors and association with empathy and self-efficacy. Revista brasileira de enfermagem 73(1), e20180280. 10.1590/0034-7167-2018-0280.
96. Butera S, Brasseur N, Filion N, Bruyneel A, Smith P (2021) Prevalence and Associated Factors of Burnout Risk Among Intensive Care and Emergency Nurses Before and During the Coronavirus Disease 2019 Pandemic: A Cross-Sectional Study in Belgium. Journal of emergency nursing 47(6), 879–891. 10.1016/j.jen.2021.08.007.
97. Bruyneel A, Bouckaert N, Maertens de Noordhout C, Detollenaere J, Kohn L, Pirson M, Sermeus W, van den Heede K (2023) Association of burnout and intention-to-leave the profession with work environment: A nationwide cross-sectional study among Belgian intensive care nurses after two years of pandemic. International journal of nursing studies 137, 104385. 10.1016/j.ijnurstu.2022.104385.
98. Yıldız E (2023) Psychopathological Factors Associated With Burnout in Intensive Care Nurses: A Cross-Sectional Study. Journal of the American Psychiatric Nurses Association 29(2), 122–135. 10.1177/1078390321999725.
99. Ching Sin Siau Lei-Hum Wee1, Norhayati Ibrahim, Uma Visvalingam2, Lena Lay Ling Yeap3, Seen Heng PREDICTING BURNOUT AND PSYCHOLOGICAL DISTRESS RISKS OF

HOSPITAL HEALTHCARE WORKERS. Malaysian Journal of Public Health Medicine 2018, Special Volume (1): 125-136.
100. Lasebikan VO, Oyetunde MO (2012) Burnout among Nurses in a Nigerian General Hospital: Prevalence and Associated Factors. ISRN Nursing 2012. 10.5402/2012/402157.
101. Bressi C, Manenti S, Porcellana M, Cevales D, Farina L, Felicioni I, Meloni G, Milone G, Miccolis IR, Pavanetto M, Pescador L, Poddigue M, Scotti L, Zambon A, Corrao G, Lambertenghi-Deliliers G, Invernizzi G (2008) Haemato-oncology and burnout: an Italian survey. British journal of cancer 98(6), 1046–1052. 10.1038/sj.bjc.6604270.
102. Escribà-Agüir V, Pérez-Hoyos S (2007) Psychological well-being and psychosocial work environment characteristics among emergency medical and nursing staff. Stress and Health 23(3), 153–160. 10.1002/smi.1131.
103. Escribà-Agüir V, Martín-Baena D, Pérez-Hoyos S (2006) Psychosocial work environment and burnout among emergency medical and nursing staff. International archives of occupational and environmental health 80(2), 127–133. 10.1007/s00420-006-0110-y.
104. Alvares MEM, Thomaz EBAF, Lamy ZC, Nina RVdAH, Pereira MUL, Garcia JBS (2020) Síndrome de burnout entre profissionais de saúde nas unidades de terapia intensiva: um estudo transversal com base populacional. Revista Brasileira de terapia intensiva 32(2), 251–260. 10.5935/0103-507x.20200036.
105. Filho FA, Rodrigues MCS, Cimiotti JP (2019) Burnout in Brazilian Intensive Care Units: A Comparison of Nurses and Nurse Technicians. AACN advanced critical care 30(1), 16–21. 10.4037/aacnacc2019222.
106. Jiménez RE, Bachelet VC, Gomolán P, Lefio LÁ, Goyenechea M (2019) Violence and burnout in health care emergency workers in Santiago, Chile: A survey-based cross-sectional study. International emergency nursing 47, 100792.
107. Sundin L, Hochwälder J, Lisspers J (2011) A longitudinal examination of generic and occupational specific job demands, and work-related social support associated with burnout among nurses in Sweden. Work (Reading, Mass.) 38(4), 389–400. 10.3233/WOR-2011-1142.
108. Leineweber C, Westerlund H, Chungkham HS, Lindqvist R, Runesdotter S, Tishelman C (2014) Nurses' practice environment and work-family conflict in relation to burn out: a multilevel modelling approach. PloS one 9(5), e96991. 10.1371/journal.pone.0096991.
109. Kowalski C, Ommen O, Driller E, Ernstmann N, Wirtz MA, Köhler T, Pfaff H (2010) Burnout in nurses - the relationship between social capital in hospitals and emotional exhaustion. Journal of clinical nursing 19(11-12), 1654–1663. 10.1111/j.1365-2702.2009.02989.x.
110. Nantsupawat A, Kunaviktikul W, Nantsupawat R, Wichaikhum O-A, Thienthong H, Poghosyan L (2017) Effects of nurse work environment on job dissatisfaction, burnout, intention to leave. International Nursing Review 64(1), 91–98. 10.1111/inr.12342.
111. Nantsupawat A, Srisuphan W, Kunaviktikul W, Wichaikhum O-A, Aungsuroch Y, Aiken LH (2011) Impact of nurse work environment and staffing on hospital nurse and quality of care in Thailand. Journal of nursing scholarship an official publication of Sigma Theta Tau International Honor Society of Nursing 43(4), 426–432. 10.1111/j.1547-5069.2011.01419.x.
112. Vidotti V, Ribeiro RP, Galdino MJQ, Martins JT (2018) Burnout Syndrome and shift work among the nursing staff. Revista latino-americana de enfermagem 26, e3022. 10.1590/1518-8345.2550.3022.
113. Zhang L-F, You L-M, Liu K, Zheng J, Fang J-B, Lu M-M, Lv A-L, Ma W-G, Wang J, Wang S-H, Wu X, Zhu X-W, Bu X-Q (2014) The association of Chinese hospital work environment with nurse burnout, job satisfaction, and intention to leave. Nursing outlook 62(2), 128–137. 10.1016/j.outlook.2013.10.010.
114. Chen R, Sun C, Chen J-J, Jen H-J, Kang XL, Kao C-C, Chou K-R (2021) A Large-Scale Survey on Trauma, Burnout, and Posttraumatic Growth among Nurses during the COVID-19 Pandemic. International journal of mental health nursing 30(1), 102–116. 10.1111/inm.12796.
115. Ntantana A, Matamis D, Savvidou S, Giannakou M, Gouva M, Nakos G, Koulouras V (2017) Burnout and job satisfaction of intensive care personnel and the relationship with personality and

religious traits: An observational, multicenter, cross-sectional study. Intensive & critical care nursing 41, 11–17. 10.1016/j.iccn.2017.02.009.
116. Hanrahan NP, Aiken LH, McClaine L, Hanlon AL (2010) Relationship between psychiatric nurse work environments and nurse burnout in acute care general hospitals. Issues in mental health nursing 31(3), 198–207. 10.3109/01612840903200068.
117. Stimpfel AW, Sloane DM, Aiken LH (2012) The longer the shifts for hospital nurses, the higher the levels of burnout and patient dissatisfaction. Health affairs (Project Hope) 31(11), 2501–2509. 10.1377/hlthaff.2011.1377.
118. Madathil R, Heck NC, Schuldberg D (2014) Burnout in psychiatric nursing: examining the interplay of autonomy, leadership style, and depressive symptoms. Archives of psychiatric nursing 28(3), 160–166. 10.1016/j.apnu.2014.01.002.
119. Yoon HS, Sok SR (2016) Experiences of violence, burnout and job satisfaction in Korean nurses in the emergency medical centre setting. International journal of nursing practice 22(6), 596–604. 10.1111/ijn.12479.
120. Abdo SAM, El-Sallamy RM, El-Sherbiny AAM, Kabbash IA (2016) Burnout among physicians and nursing staff working in the emergency hospital of Tanta University, Egypt. Eastern Mediterranean health journal = La revue de sante de la Mediterranee orientale = al-Majallah al-sihhiyah li-sharq al-mutawassit 21(12), 906–915. 10.26719/2015.21.12.906.
121. Gunnarsdóttir S, Clarke SP, Rafferty AM, Nutbeam D (2009) Front-line management, staffing and nurse-doctor relationships as predictors of nurse and patient outcomes. a survey of Icelandic hospital nurses. International journal of nursing studies 46(7), 920–927. 10.1016/j.ijnurstu.2006.11.007.
122. Giusti EM, Pedroli E, D'Aniello GE, Stramba Badiale C, Pietrabissa G, Manna C, Stramba Badiale M, Riva G, Castelnuovo G, Molinari E (2020) The Psychological Impact of the COVID-19 Outbreak on Health Professionals: A Cross-Sectional Study. Front. Psychol. 11, 1684. 10.3389/fpsyg.2020.01684.
123. Rees CS, Eley R, Osseiran-Moisson R, Francis K, Cusack L, Heritage B, Hegney D (2019) Individual and environmental determinants of burnout among nurses. Journal of health services research & policy 24(3), 191–200. 10.1177/1355819619840373.
124. Al Sabei S, AbuAlRub R, Al Yahyaei A, Al-Rawajfah OM, Labrague LJ, Burney IA, Al-Maqbali M (2023) The influence of nurse managers' authentic leadership style and work environment characteristics on job burnout among emergency nurses. International emergency nursing 70, 101321. 10.1016/j.ienj.2023.101321.
125. García-Sierra R, Fernández-Castro J, Martínez-Zaragoza F (2016) Relationship between job demand and burnout in nurses: does it depend on work engagement? Journal of nursing management 24(6), 780–788. 10.1111/jonm.12382.
126. van Bogaert P, Clarke S, Roelant E, Meulemans H, van de Heyning P (2010) Impacts of unit-level nurse practice environment and burnout on nurse-reported outcomes: a multilevel modelling approach. Journal of clinical nursing 19(11-12), 1664–1674. 10.1111/j.1365-2702.2009.03128.x.
127. Manzano García G, Ayala Calvo JC (2021) The threat of COVID-19 and its influence on nursing staff burnout. Journal of advanced nursing 77(2), 832–844. 10.1111/jan.14642.
128. Sterud T, Hem E, Lau B, Ekeberg O (2011) A comparison of general and ambulance specific stressors: predictors of job satisfaction and health problems in a nationwide one-year follow-up study of Norwegian ambulance personnel. Journal of occupational medicine and toxicology (London, England) 6(1), 10. 10.1186/1745-6673-6-10.
129. University of texas, Health Science Center at Houston, School of Public Health (2016) Work-Related VIolence Research Project: Overview and Survey module and Focus Group Findings.
130. Department of Economic and Social Affairs, United Nations (2014) Guidelines for Producing Statistics on Violence against Women.
131. INTERNATIONAL LABOUR OFFICE, ILO Department of Statistics (2013) Work-related violence and its integration into existing surveys: 19th International Conference of Labour Statisticians.

132. Winters T, Canadioan Union of Public Employers Conducting Surveys on Violence in the Workplace.
133. Yusoff HM, Ahmad H, Ismail H, Reffin N, Chan D, Kusnin F, Bahari N, Baharudin H, Aris A, Shen HZ, Rahman MA (2023) Contemporary evidence of workplace violence against the primary healthcare workforce worldwide: a systematic review. Human resources for health 21(1), 82. 10.1186/s12960-023-00868-8.
134. Stafford S, Avsar P, Nugent L, O'Connor T, Moore Z, Patton D, Watson C (2022) What is the impact of patient violence in the emergency department on emergency nurses' intention to leave? Journal of nursing management 30(6), 1852–1860. 10.1111/jonm.13728.
135. Wang J, Zeng Q, Wang Y, Liao X, Xie C, Wang G, Zeng Y (2022) Workplace violence and the risk of post-traumatic stress disorder and burnout among nurses: A systematic review and meta-analysis. Journal of nursing management 30(7), 2854–2868. 10.1111/jonm.13809.
136. Sahebi A, Golitaleb M, Moayedi S, Torres M, Sheikhbardsiri H (2022) Prevalence of workplace violence against health care workers in hospital and pre-hospital settings: An umbrella review of meta-analyses. Front. Public Health 10. 10.3389/fpubh.2022.895818.
137. Furin M, Eliseo LJ, Langlois B, Fernandez WG, Mitchell P, Dyer KS (2015) Self-reported provider safety in an urban emergency medical system. The western journal of emergency medicine 16(3), 459–464. 10.5811/westjem.2015.2.24124.
138. Koritsas S, Boyle M, Coles J (2009) Factors associated with workplace violence in paramedics. Prehospital and disaster medicine 24(5), 417–421. 10.1017/s1049023x0000724x.
139. Boyle M, Koritsas S, Coles J, Stanley J (2007) A pilot study of workplace violence towards paramedics. Emergency medicine journal EMJ 24(11), 760–763. 10.1136/emj.2007.046789.
140. Brough P (2005) Workplace violence experienced by paramedics: Relationship with social support, job satisfaction and psychological strain. Australia.
141. Braun D, Reifferscheid F, Kerner T, Dressler JL, Stuhr M, Wenderoth S, Petrowski K (2021) Association between the experience of violence and burnout among paramedics. International archives of occupational and environmental health 94(7), 1559–1565. 10.1007/s00420-021-01693-z.
142. Kim S, Kitzmiller R, Baernholdt M, Lynn MR, Jones CB (2023) Patient Safety Culture: The Impact on Workplace Violence and Health Worker Burnout. Workplace health & safety 71(2), 78–88. 10.1177/21650799221126364.
143. Kim W, Bae M, Chang S-J, Yoon J-H, Da Jeong Y, Hyun D-S, Ryu H-Y, Park K-S, Kim M-J, Kim C (2019) Effect of Burnout on Post-traumatic Stress Disorder Symptoms Among Firefighters in Korea: Data From the Firefighter Research on Enhancement of Safety & Health (FRESH). Journal of preventive medicine and public health = Yebang Uihakhoe chi 52(6), 345–354. 10.3961/jpmph.19.116.
144. Deniz T, Saygun M, Eroğlu O, Ülger H, Azapoğlu B (2016) Effect of exposure to violence on the development of burnoutsyndrome in ambulance staff. Turkish journal of medical sciences 46(2), 296–302. 10.3906/sag-1406-53.
145. Bigham BL, Jensen JL, Tavares W, Drennan IR, Saleem H, Dainty KN, Munro G (2014) Paramedic self-reported exposure to violence in the emergency medical services (EMS) workplace: a mixed-methods cross-sectional survey. Prehospital emergency care 18(4), 489–494. 10.3109/10903127.2014.912703.
146. Gómez-Gutiérrez MM, Bernaldo-De-Quirós M, Piccini AT, Cerdeira JC (2016) Posttraumatic Stress Symptomatology in Pre-Hospital Emergency Care Professionals Assaulted by Patients and/or Relatives: Importance of Severity and Experience of the Aggression. Journal of interpersonal violence 31(2), 339–354. 10.1177/0886260514555370.
147. Gormley MA, Crowe RP, Bentley MA, Levine R (2016) A National Description of Violence toward Emergency Medical Services Personnel. Prehospital emergency care 20(4), 439–447. 10.3109/10903127.2015.1128029.

148. Rahmani A, Hassankhani H, Mills J, Dadashzadeh A (2012) Exposure of Iranian emergency medical technicians to workplace violence: a cross-sectional analysis. Emerg Medicine Australasia 24(1), 105–110. 10.1111/j.1742-6723.2011.01494.x.
149. Petzäll K, Tällberg J, Lundin T, Suserud B-O (2011) Threats and violence in the Swedish pre-hospital emergency care. International emergency nursing 19(1), 5–11. 10.1016/j.ienj.2010.01.004.
150. Petersen S, Scheller B, Wutzler S, Zacharowski K, Wicker S (2016) Aggression und subjektive Gefährdung in der Notfallmedizin Eine Umfrage. Der Anaesthesist 65(8), 580–584. 10.1007/s00101-016-0202-3.
151. Lindner T, Joachim R, Bieberstein S, Schiffer H, Möckel M, Searle J (2015) Aggressives und herausforderndes Verhalten gegenüber dem Klinikpersonal. Notfall Rettungsmed 18(3), 195–200. 10.1007/s10049-015-1982-8.
152. Suserud BO, Blomquist M, Johansson I (2002) Experiences of threats and violence in the Swedish ambulance service. Accident and emergency nursing 10(3), 127–135. 10.1054/aaen.2002.0361.
153. Musengamana V, Adejumo O, Banamwana G, Mukagendaneza MJ, Twahirwa TS, Munyaneza E, Kabakambira JD (2022) Workplace violence experience among nurses at a selected university teaching hospital in Rwanda. The Pan African medical journal 41, 64. 10.11604/pamj.2022.41.64.30865.
154. Alameddine M, Mourad Y, Dimassi H (2015) A National Study on Nurses' Exposure to Occupational Violence in Lebanon: Prevalence, Consequences and Associated Factors. PloS one 10(9), e0137105. 10.1371/journal.pone.0137105.
155. Bernaldo-De-Quirós M, Piccini AT, Gómez MM, Cerdeira JC (2015) Psychological consequences of aggression in pre-hospital emergency care: cross sectional survey. International journal of nursing studies 52(1), 260–270. 10.1016/j.ijnurstu.2014.05.011.
156. Hamdan M, Abu Hamra A (2015) Workplace violence towards workers in the emergency departments of Palestinian hospitals: a cross-sectional study. Human resources for health 13, 28. 10.1186/s12960-015-0018-2.
157. Hu SX, Luk AL, Smith GD (2015) The effects of hazardous working conditions on burnout in Macau nurses. International Journal of Nursing Sciences 2(1), 86–92. 10.1016/j.ijnss.2015.01.006.
158. Looff P de, Nijman H, Didden R, Embregts P (2018) Burnout symptoms in forensic psychiatric nurses and their associations with personality, emotional intelligence and client aggression: A cross-sectional study. Journal of psychiatric and mental health nursing 25(8), 506–516. 10.1111/jpm.12496.
159. Looff P de, Didden R, Embregts P, Nijman H (2019) Burnout symptoms in forensic mental health nurses: Results from a longitudinal study. International journal of mental health nursing 28(1), 306–317. 10.1111/inm.12536.
160. Liu W, Zhao S, Shi L, Zhang Z, Liu X, Li L, Duan X, Li G, Lou F, Jia X, Fan L, Sun T, Ni X (2018) Workplace violence, job satisfaction, burnout, perceived organisational support and their effects on turnover intention among Chinese nurses in tertiary hospitals: a cross-sectional study. BMJ open 8(6), e019525. 10.1136/bmjopen-2017-019525.
161. Liu J, Zheng J, Liu K, Liu X, Wu Y, Wang J, You L (2019) Workplace violence against nurses, job satisfaction, burnout, and patient safety in Chinese hospitals. Nursing outlook 67(5), 558–566. 10.1016/j.outlook.2019.04.006.
162. Merecz D, Rymaszewska J, Mościcka A, Kiejna A, Jarosz-Nowak J (2006) Violence at the workplace--a questionnaire survey of nurses. European psychiatry the journal of the Association of European Psychiatrists 21(7), 442–450. 10.1016/j.eurpsy.2006.01.001.
163. Grinberg K, Revach C, Lipsman G (2022) Violence in hospitals and burnout among nursing staff. International emergency nursing 65, 101230. 10.1016/j.ienj.2022.101230.
164. Llor-Esteban B, Sánchez-Muñoz M, Ruiz-Hernández JA, Jiménez-Barbero JA (2017) User violence towards nursing professionals in mental health services and emergency units. The European Journal of Psychology Applied to Legal Context 9(1), 33–40. 10.1016/j.ejpal.2016.06.002.

165. Gascon S, Leiter MP, Andrés E, Santed MA, Pereira JP, Cunha MJ, Albesa A, Montero-Marín J, García-Campayo J, Martínez-Jarreta B (2013) The role of aggressions suffered by healthcare workers as predictors of burnout. Journal of clinical nursing 22(21-22), 3120–3129. 10.1111/j.1365-2702.2012.04255.x.
166. Schablon A, Zeh A, Wendeler D, Peters C, Wohlert C, Harling M, Nienhaus A (2012) Frequency and consequences of violence and aggression towards employees in the German healthcare and welfare system: a cross-sectional study. BMJ open 2(5). 10.1136/bmjopen-2012-001420.
167. Stahl-Gugger A, Hämmig O (2022) Prevalence and health correlates of workplace violence and discrimination against hospital employees - a cross-sectional study in German-speaking Switzerland. BMC health services research 22(1), 291. 10.1186/s12913-022-07602-5.
168. Savoy S, Carron P-N, Romain-Glassey N, Beysard N (2021) Self-Reported Violence Experienced by Swiss Prehospital Emergency Care Providers. Emergency medicine international 2021, 9966950. 10.1155/2021/9966950.
169. Li J-H, Chen T-W, Lee H-F, Shih W-M (2021) The Effects of Emergency Room Violence toward Nurse's Intention to Leave-Resilience as a Mediator. Healthcare (Basel, Switzerland) 9(5). 10.3390/healthcare9050507.
170. Li N, Zhang L, Xiao G, Chen ZJ, Lu Q (2020) Effects of organizational commitment, job satisfaction and workplace violence on turnover intention of emergency nurses: A cross-sectional study. International journal of nursing practice 26(6), e12854. 10.1111/ijn.12854.
171. Li N, Zhang L, Xiao G, Chen J, Lu Q (2019) The relationship between workplace violence, job satisfaction and turnover intention in emergency nurses. International emergency nursing 45, 50–55. 10.1016/j.ienj.2019.02.001.
172. Bordignon M, Monteiro MI (2019) Predictors of nursing workers' intention to leave the work unit, health institution and profession. Revista latino-americana de enfermagem 27, e3219. 10.1590/1518-8345.3280.3219.
173. Fang H, Zhao X, Yang H, Sun P, Li Y, Jiang K, Li P, Jiao M, Liu M, Qiao H, Wu Q (2018) Depressive symptoms and workplace-violence-related risk factors among otorhinolaryngology nurses and physicians in Northern China: a cross-sectional study. BMJ open 8(1), e019514. 10.1136/bmjopen-2017-019514.
174. Jeong I-Y, Kim J-S (2018) The relationship between intention to leave the hospital and coping methods of emergency nurses after workplace violence. Journal of clinical nursing 27(7-8), 1692–1701. 10.1111/jocn.14228.
175. Li Y-F, Chao M, Shih C-T (2018) Nurses' intention to resign and avoidance of emergency department violence: A moderated mediation model. International emergency nursing 39, 55–61. 10.1016/j.ienj.2017.09.004.
176. Schablon A, Wendeler D, Kozak A, Nienhaus A, Steinke S (2018) Prevalence and Consequences of Aggression and Violence towards Nursing and Care Staff in Germany-A Survey. International journal of environmental research and public health 15(6). 10.3390/ijerph15061274.
177. Zhao S, Xie F, Wang J, Shi Y, Zhang S, Han X, Sun Z, Shi L, Li Z, Mu H, Liu X, Liu W, Gao L, Sun T, Fan L (2018) Prevalence of Workplace Violence Against Chinese Nurses and Its Association with Mental Health: A Cross-sectional Survey. Archives of psychiatric nursing 32(2), 242–247. 10.1016/j.apnu.2017.11.009.
178. Choi S-H, Lee H (2017) Workplace violence against nurses in Korea and its impact on professional quality of life and turnover intention. Journal of nursing management 25(7), 508–518. 10.1111/jonm.12488.
179. Ferri P, Silvestri M, Artoni C, Di Lorenzo R (2016) Workplace violence in different settings and among various health professionals in an Italian general hospital: a cross-sectional study. Psychology research and behavior management 9, 263–275. 10.2147/PRBM.S114870.
180. Frick J, Slagman A, Lomberg L, Searle J, Möckel M, Lindner T (2016) Sicherheitsinfrastruktur in deutschen Notaufnahmen. Notfall Rettungsmed 19(8), 666–670. 10.1007/s10049-016-0179-0.

181. Speroni KG, Fitch T, Dawson E, Dugan L, Atherton M (2014) Incidence and cost of nurse workplace violence perpetrated by hospital patients or patient visitors. Journal of emergency nursing 40(3), 218-28; quiz 295. 10.1016/j.jen.2013.05.014.
182. Pai H-C, Lee S (2011) Risk factors for workplace violence in clinical registered nurses in Taiwan. Journal of clinical nursing 20(9-10), 1405–1412. 10.1111/j.1365-2702.2010.03650.x.
183. Al-Omari H (2015) Physical and verbal workplace violence against nurses in Jordan. International Nursing Review 62(1), 111–118. 10.1111/inr.12170.
184. Lee H-L, Han C-Y, Redley B, Lin C-C, Lee M-Y, Chang W (2020) Workplace Violence Against Emergency Nurses in Taiwan: A Cross-Sectional Study. Journal of emergency nursing 46(1), 66-71.e4. 10.1016/j.jen.2019.09.004.
185. Guan R, Gao J, Liu G, Cheng F, Ge B (2019) The Mediating Effect of Perceived Social Acknowledgment on the Relationship Between Patient Assaults and Posttraumatic Stress Reactions in Emergency Nurses. Journal of interpersonal violence 34(18), 3833–3849. 10.1177/0886260516673627.
186. Noorana Zahra A, Feng J-Y (2018) Workplace violence against nurses in Indonesian emergency departments. Enfermería Clínica 28, 184–190. 10.1016/S1130-8621(18)30064-0.
187. Gillespie GL, Pekar B, Byczkowski TL, Fisher BS (2017) Worker, workplace, and community/environmental risk factors for workplace violence in emergency departments. Archives of environmental & occupational health 72(2), 79–86. 10.1080/19338244.2016.1160861.
188. Partridge B, Affleck J (2017) Verbal abuse and physical assault in the emergency department: Rates of violence, perceptions of safety, and attitudes towards security. Australasian emergency nursing journal AENJ 20(3), 139–145. 10.1016/j.aenj.2017.05.001.
189. Pich JV, Kable A, Hazelton M (2017) Antecedents and precipitants of patient-related violence in the emergency department: Results from the Australian VENT Study (Violence in Emergency Nursing and Triage). Australasian emergency nursing journal AENJ 20(3), 107–113. 10.1016/j.aenj.2017.05.005.
190. ALBashtawy M, Aljezawi M (2016) Emergency nurses' perspective of workplace violence in Jordanian hospitals: A national survey. International emergency nursing 24, 61–65. 10.1016/j.ienj.2015.06.005.
191. Alyaemni A, Alhudaithi H (2016) Workplace violence against nurses in the emergency departments of three hospitals in Riyadh, Saudi Arabia: A cross-sectional survey. NursingPlus Open 2, 35–41. 10.1016/j.npls.2016.09.001.
192. Abou-ElWafa HS, El-Gilany A-H, Abd-El-Raouf SE, Abd-Elmouty SM, El-Sayed RE-SH (2015) Workplace violence against emergency versus non-emergency nurses in Mansoura university hospitals, Egypt. Journal of interpersonal violence 30(5), 857–872. 10.1177/0886260514536278.
193. Darawad MW, Al-Hussami M, Saleh AM, Mustafa WM, Odeh H (2015) Violence against nurses in emergency departments in jordan: nurses' perspective. Workplace health & safety 63(1), 9–17. 10.1177/2165079914565348.
194. Stene J, Larson E, Levy M, Dohlman M (2015) Workplace violence in the emergency department: giving staff the tools and support to report. The Permanente journal 19(2), e113-7. 10.7812/tpp/14-187.
195. ALBashtawy M (2013) Workplace violence against nurses in emergency departments in Jordan. International Nursing Review 60(4), 550–555. 10.1111/inr.12059.
196. Franz S, Zeh A, Schablon A, Kuhnert S, Nienhaus A (2010) Aggression and violence against health care workers in Germany--a cross sectional retrospective survey. BMC health services research 10, 51. 10.1186/1472-6963-10-51.
197. Mambrey V, Ritz-Timme S, Loerbroks A (2023) Prevalence and correlates of workplace violence against medical assistants in Germany: a cross-sectional study. BMC health services research 23(1), 350. 10.1186/s12913-023-09331-9.
198. Pihl-Thingvad J, Elklit A, Brandt LPA, Andersen LL (2019) Workplace violence and development of burnout symptoms: a prospective cohort study on 1823 social educators. International archives of occupational and environmental health 92(6), 843–853. 10.1007/s00420-019-01424-5.

199. American Psychological Association (accessed 2024; Updated on 2023) posttraumatic stress disorder (PTSD). https://dictionary.apa.org/posttraumatic-stress-disorder.
200. Mayo Clinic (2022) Symptoms and causes. https://www.mayoclinic.org/diseases-conditions/post-traumatic-stress-disorder/symptoms-causes/syc-20355967.
201. D'Ettorre G, Pellicani V, Ceccarelli G (2020) Post-traumatic stress disorder symptoms in healthcare workers: a ten-year systematic review. Acta bio-medica Atenei Parmensis 91(12-S), e2020009. 10.23750/abm.v91i12-S.9459.
202. Hilton NZ, Addison S, Ham E, C Rodrigues N, Seto MC (2022) Workplace violence and risk factors for PTSD among psychiatric nurses: Systematic review and directions for future research and practice. Journal of psychiatric and mental health nursing 29(2), 186–203. 10.1111/jpm.12781.
203. Hansen NB, Vang ML, Lichtenstein MB, Pihl-Thingvad J (2022) Workplace Sexual Harassment Increases the Risk of PTSD Symptoms with Higher Frequency and Harassment Coming from a Colleague or Leader as Risk Factors. Scandinavian Journal of Work and Organizational Psychology 7(1). 10.16993/sjwop.174.
204. Martinmäki SE, Jong K de, Komproe IH, Boelen PA, Kleber RJ (2023) Incidence and Severity of Sexual Harassment, and its Impact on Mental Health in a Cohort of International Humanitarian Field-Workers. Journal of interpersonal violence 38(11-12), 7426–7456. 10.1177/08862605221145954.
205. Blizzard SJ, Kemppainen J, Taylor J (2009) Posttraumatic stress disorder and community violence: An update for nurse practitioners. Journal of the American Academy of Nurse Practitioners 21(10), 535–541. 10.1111/j.1745-7599.2009.00442.x.
206. May CL, Wisco BE (2016) Defining trauma: How level of exposure and proximity affect risk for posttraumatic stress disorder. Psychological trauma theory, research, practice and policy 8(2), 233–240. 10.1037/tra0000077.
207. Laeeque SH, Bilal A, Babar S, Khan Z, Ul Rahman S (2018) How Patient-Perpetrated Workplace Violence Leads to Turnover Intention Among Nurses: The Mediating Mechanism of Occupational Stress and Burnout. Journal of Aggression, Maltreatment & Trauma 27(1), 96–118. 10.1080/10926771.2017.1410751.
208. Converso D, Sottimano I, Balducci C (2021) Violence exposure and burnout in healthcare sector: mediating role of work ability. La Medicina del lavoro 112(1), 58–67. 10.23749/mdl.v112i1.9906.
209. Demerouti E, Bakker AB, Nachreiner F, Schaufeli WB (2001) The job demands-resources model of burnout. Journal of Applied Psychology 86(3), 499–512. 10.1037/0021-9010.86.3.499.
210. Viotti S, Gilardi S, Guglielmetti C, Converso D (2015) Verbal Aggression from Care Recipients as a Risk Factor among Nursing Staff: A Study on Burnout in the JD-R Model Perspective. BioMed research international 2015, 215267. 10.1155/2015/215267.
211. Maslach C, Leiter MP (2016) Understanding the burnout experience: recent research and its implications for psychiatry. World psychiatry official journal of the World Psychiatric Association (WPA) 15(2), 103–111. 10.1002/wps.20311.
212. Schaufeli WB, Leiter MP, Maslach C (2009) Burnout: 35 years of research and practice. Career Development International 14(3), 204–220. 10.1108/13620430910966406.
213. Shirey MR (2006) Stress and coping in nurse managers: two decades of research. Nursing economic$ 24(4), 193-203, 211 passim.
214. Kohler JM, Munz DC, Grawitch MJ (2006) Test of a Dynamic Stress Model for Organisational Change: Do Males and Females Require Different Models? Applied Psychology 55(2), 168–191. 10.1111/j.1464-0597.2006.00229.x.
215. Coyle D, Edwards D, Hannigan B, Fothergill A, Burnard P (2005) A systematic review of stress among mental health social workers. International Social Work 48(2), 201–211. 10.1177/0020872805050492.
216. Pihl-Thingvad J, Andersen LL, Brandt LPA, Elklit A (2019) Are frequency and severity of workplace violence etiologic factors of posttraumatic stress disorder? A 1-year prospective study of 1,763 social educators. Journal of occupational health psychology 24(5), 543–555. 10.1037/ocp0000148.

217. Jankovic M, Sijtsema JJ, Reitz AK, Masthoff ED, Bogaerts S (2021) Workplace violence, post-traumatic stress disorder symptoms, and personality. Personality and Individual Differences 168, 110410. 10.1016/j.paid.2020.110410.
218. Kunst MJJ, Winkel FW, Bogaerts S (2010) Posttraumatic Growth Moderates the Association Between Violent Revictimization and Persisting PTSD Symptoms in Victims of Interpersonal Violence: A Six-Month Follow-Up Study. Journal of Social and Clinical Psychology 29(5), 527–545. 10.1521/jscp.2010.29.5.527.
219. Mitani S, Fujita M, Nakata K, Shirakawa T (2006) Impact of post-traumatic stress disorder and job-related stress on burnout: a study of fire service workers. The Journal of emergency medicine 31(1), 7–11. 10.1016/j.jemermed.2005.08.008.
220. Ogle CM, Rubin DC, Berntsen D, Siegler IC (2013) The Frequency and Impact of Exposure to Potentially Traumatic Events Over the Life Course. Clinical psychological science a journal of the Association for Psychological Science 1(4), 426–434. 10.1177/2167702613485076.
221. Fitzpatrick S, Saraiya T, Lopez-Castro T, Ruglass LM, Hien D (2020) The impact of trauma characteristics on post-traumatic stress disorder and substance use disorder outcomes across integrated and substance use treatments. Journal of substance abuse treatment 113, 107976. 10.1016/j.jsat.2020.01.012.
