# Supplementary material for: Impact of violence on emotional exhaustion risk of registered nurses in Germany: a Bayesian analysis of cross-sectional data with multiple imputations
Source: BMC Nurs. 2025 Aug 18;24:1080. doi: 10.1186/s12912-025-03745-y (PMC12359958; doi:10.1186/s12912-025-03745-y)
Supplement: Supplementary file 3 — Supplementary Material 3 [file 12912_2025_3745_MOESM3_ESM.pdf]

Appendix C – technical details

Appendix C1 – Multiple Imputation

Missing data (missingness) were addressed using multiple imputed chained equations (MICE) with fully conditional specification (FCS) within the `mice` package in RStudio (version 4.3.3) [1].

Missingness on multi-item composite scores (EE; PES-subdimensions) were addressed by using passive imputation, which yield more stable estimates and exploit differential missingness between the subscales [2, 3]. The algorithm is explained below. Except for EE, all variables with missing data will be assumed missing at random (MAR) based on either high response rates or the availability of an extensive pool of auxiliary variables measuring similar constructs that were often non-missing. Due to machine-based reading of survey forms, missingness codes were differentiable between "missing" or "not readable". Entries "not readable" are assumed to be missing completely at random (MCAR).

A total of 37 auxiliary variables were considered, including: eight patient safety variables, DP and PA, patient injury, workplace injury and complaint frequency, number of patients and their needs, intention to leave, self-rated quality of different nursing aspects, a sum score of missed tasks and more. Other auxiliaries were excluded due to redundancy, high missingness, or suspected misunderstanding of the questions. Auxiliary variables were selected considering their average influx and outflux, correlation, joint model significance, and hypothesis testing of missingness indicators of the analysis variables [4]. A second-order model for missingness in auxiliary variables was defined via the `quickpred()` function included in the `mice` package. Using auxiliary variables in the imputation process increases the chances of missingness being MAR and contributes valuable information to the imputation, especially under an inclusive approach [4]. For variables that are likely missing not at random (MNAR), unbiased results cannot be guaranteed. However, with a few exceptions, MI will still outperform CC analysis and give less biased results, especially when sufficient information is available in other variables [5].

Plausibility of imputed values was ensured by employing logistic regression models to generate dichotomous variables or predictive mean matching (PMM) and random forest (RF) for ordinal and continuous variables. The choice of models for ordinal data has been extensively analyzed in different simulation studies under multiple conditions in the literature [6]. PMM was used for imputation in both analyses, following recommendations for its suitability for passive imputation and robustness to distributional assumptions [7, 8]. Exceptions were made for continuous variables using RF (e.g. work seniority, number of patients) as it lacks assumptions on normality, heteroscedasticity and collinearity. [9]. Compatibility was ensured by deploying a full prediction model. Predictive matrices implemented into FCS are found in appendix A1.

A total of 33 imputed datasets over 200 iterations were generated. The number of imputed datasets was based on percentage-points of (item-level) missing data in the analysis variables. No specific guidelines exist for the number of iterations, but a low number is typically sufficient for convergence [2]. Results from different seeds were checked for comparability and convergence (seeds 116 and 117, random seed (749) applied for analysis).

Convergence was monitored by inspecting trace plots of the posterior means and standard deviations, as well as Potential Scale Reduction Factor $\hat{R}$ (PSRF).

Passive imputation pseudo-code

Explanation:

Let Z be a set of background variables, while EE and PES are scores generated from items $EE_1, \dots, EE_9$ and $PES_1, \dots, PES_3$ respectively. After generating starting imputations, passive imputation would work implementing the following algorithm via FCS:

1. Impute Z given EE and PES
2. Impute EE_1 given $EE_2, \dots, EE_9$, Z, and PES
3. Impute EE_2 given $EE_1, EE_3, \dots, EE_9$, Z, and PES
4. Impute $EE_3, \dots, EE_9$ the same way
5. Impute PES_1 given Z $PES_2, \dots, PES_3$ and EE
6. Impute PES_2 and PES_3 the same way
7. Generate EE and PES from their items

Appendix C2 – Bayesian Analysis and Regression

A Bayesian regression analysis was chosen for both CC and MI data, because this method lends itself well to the joint analysis of multiple imputed datasets and allows for the use and comparison of prior information derived from previous literature and research.

Model explanation

The ordinal scale of EE risk was analyzed using a continuation ratio model (CRM) with a logistic link function. The CRM models an ordinal outcome as sequential stages that are reached successively, comparing conditional transitions from lower to a higher stage, such that reaching a higher stage is only possible when lower stages are reached before. In this instance, the model was formulated assuming proportional odds, resulting in proportional effects of regression estimates on transition between stages [10, 11]. The variables were entered to the linear index additively.

Prior distributions

Prior means were calculated by taking weighted averages of relevant regression weights from the literature in section 2.2. These means were then used as μ parameters for either a set of mildly informative priors with normal distributions (Prior set 1) or a set of weakly informative priors using t-student distributions (Prior set 2). Standard deviations σ and degrees of freedom df were set to always include a 0 effect, corresponding to an OR of 1. This discounted more extreme means with higher uncertainty and provided sufficiently large β ranges. A set of flat, regularizing priors with a normal distribution centered around 0 and a larger standard deviation of 1.5 were also used (Prior set 3). Prior sets 2 and 3 were used for sensitivity analysis of prior set 1. All priors are formalized and plotted in appendix A2.

Prior predictive checks

Prior predictive checks exclusively sample from the prior distribution to predict dependent categories and assess whether priors usefully mimic observed data. Priors that allow posterior sampling over a

useful range and respect the distribution of outcomes indicate acceptable prior information. Checks from the three prior sets were performed with the same number of prediction samples as iteration samples. Model predictions are represented via expected count (points) and 90% equal tailed intervals (ETI) as credible intervals (CI) (lines). Mildly informative priors set 1 predicted EE with the highest fidelity to the original data (appendix A4). The “Moderate EE” category was consistently underestimated across all prior sets, potentially due to the common practice in the literature of dichotomizing EE into “High EE” vs. lower EE categories, which influenced the generation of these priors. However, the priors allowed sampling over a useful range and maintained the relationships between category counts.

Software and computation

The models were computed using the “Bayesian Regression Models using ‘Stan’ ” (`brms`) package implementing the No-U-Turn-Sampling (NUTS) in RStudio (version 4.3.3) [12]. NUTS extends the Hamiltonian Monte Carlo (HMC) (A Markov chain Monte Carlo method) by eliminating the need for user specified parameters (such as step size and step number), to which HMC is sensible, while remaining at least as efficient as tuned sampling [13]. Seeds were chosen by random number generation and used uniformly for analysis (seed 985). Regression on CC data used 5000 warmup iterations and 10000 samples over 3 chains with thinning set to two to avoid autocorrelation along the chains. The same specifications were used for MI analysis with 20000 samples to counteract simulation uncertainty. Chain convergence and resolution were assessed with PSRF $\hat{R}$ and Effective Sample Size (ESS) measures, which are reported for CC analysis. For MI analysis, the same measures were assessed for convergence in the single imputed datasets. For all regression results, 90% posterior equal-tailed CI were used.

Appendix C3 – Missing data and missing data mechanisms

A total of 4317 responses were recorded. All analysis variables contained missing entries up to 12% in the “PES Foundations of Quality of Care” variable. The variable indicating an academic degree in nursing had 0.9% missing entries, which were manually imputed using free text entries from another item. WV variables exhibited low rates of missingness (verbal violence by patients: 1.2%; verbal violence by staff: 1.8%; physical violence by patients: 1.3%; physical violence by staff: 1.3%). After deleting one entry in self-rated quality due to a coding error, a CC sample of n=2897 (67.1%) was available.

In total, the RNs EE had missing entries in 6.3% of observations and represented the variable with the highest likelihood of being MNAR. Subscale missingness (EE 1 - EE 9) manifested as follows: EE 1: 1.2%; EE 2: 0.6%; EE 3: 1.3%; EE 4:0.9%; EE 5: 1.2%; EE 6: 1.45%; EE 7: 1.64%; EE 8: 0.8%; EE 9: 1.78%. Overall, EE sum score is considered MNAR. EE items contain mutually informative entries, which is reinforced by high internal consistency (Cronbach’s alpha) and therefore assumed MAR using a passive imputation approach and a large pool of auxiliary variables. A missingness pattern plot for EE items is shown in figure C1.

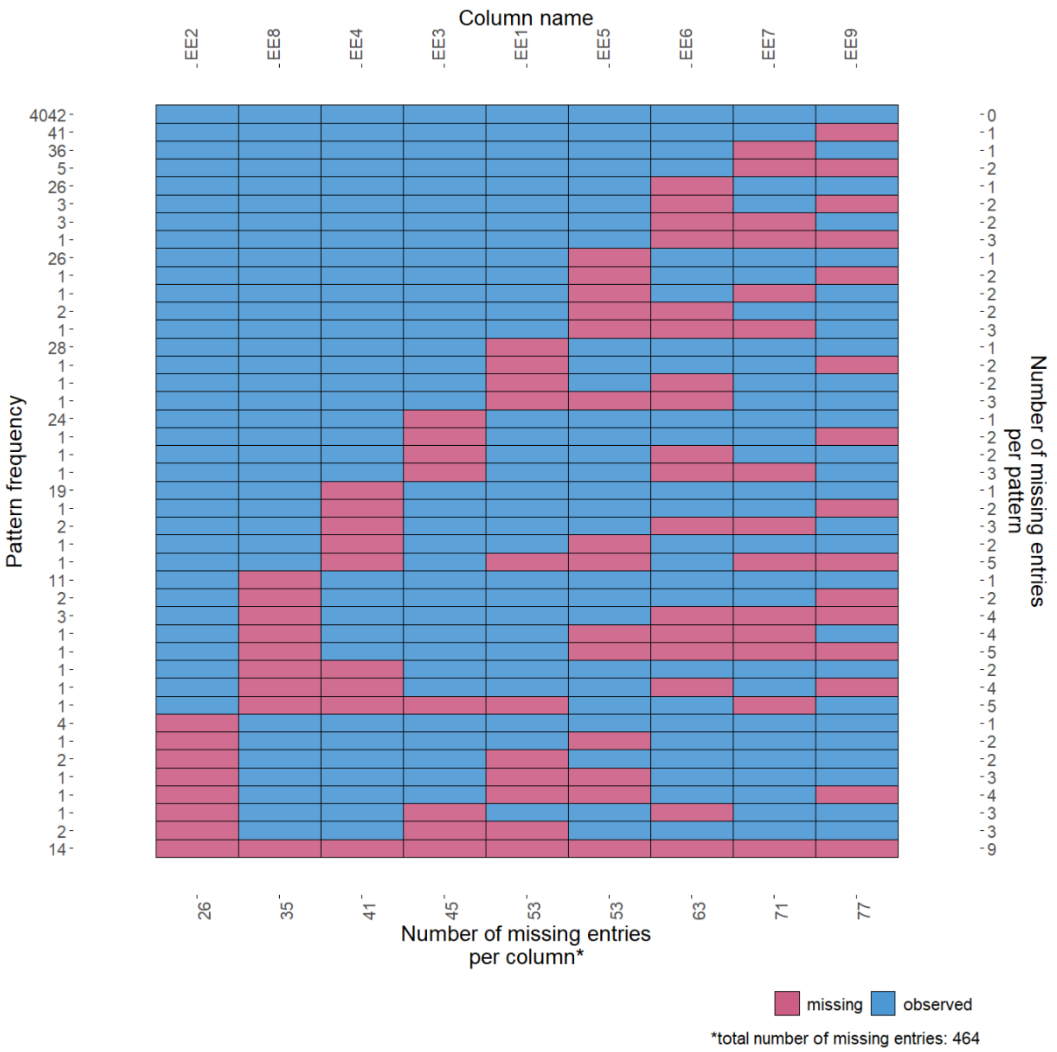

Figure C1 – Missing data pattern of EE items (RNs)

The missingness plot (figure C2) did not reveal distinct patterns. Missingness in auxiliary variables ranged from 1.2% (patient safety variable 3) to a maximum of 9.12% (DP) and not shown here.

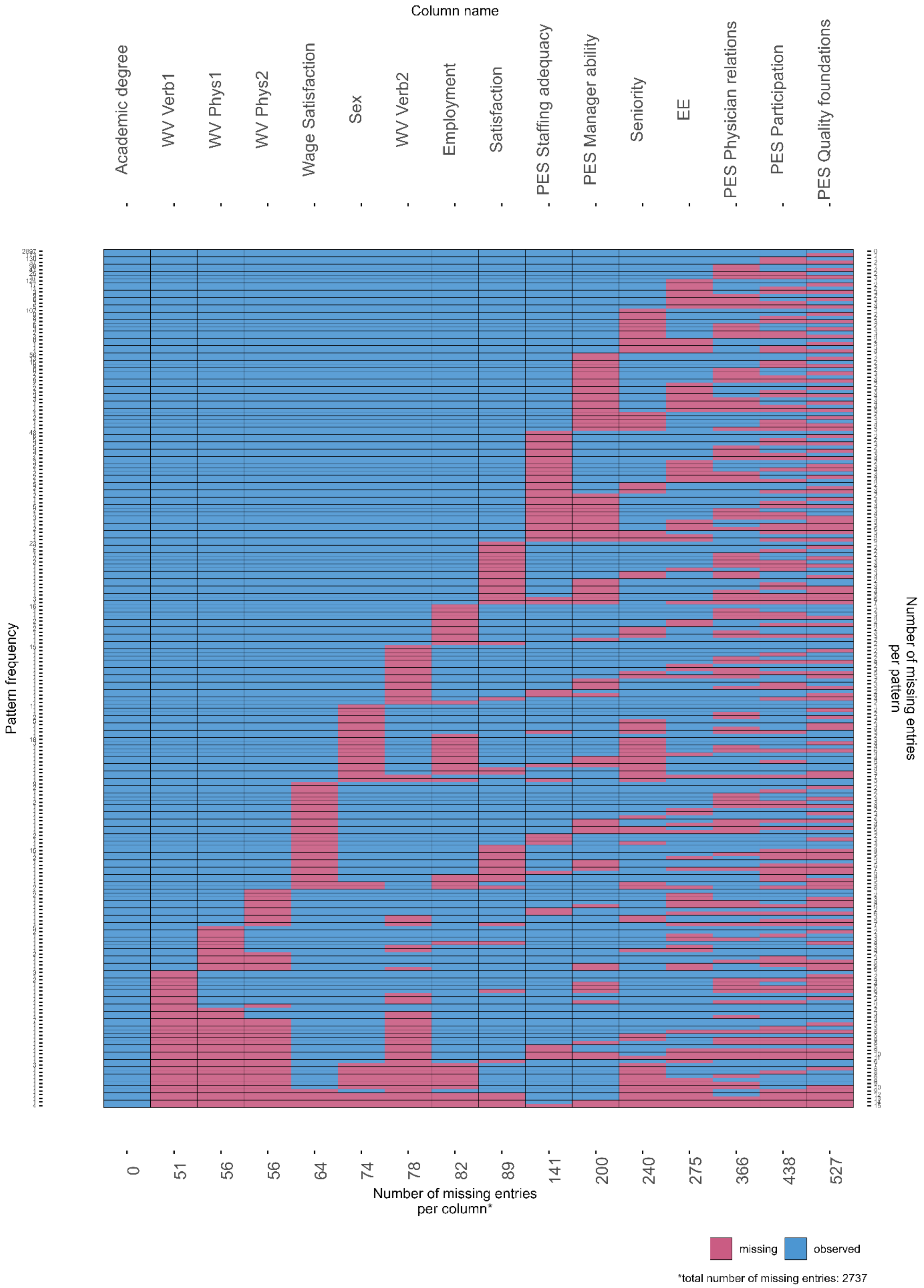

Figure C2 – Missing data pattern of RNs variables

References to appendix C

1. van Buuren S, Groothuis-Oudshoorn K (2011) mice Multivariate Imputation by Chained Equations in R. J. Stat. Soft. 45(3). 10.18637/jss.v045.i03.
2. van Buuren S (2018) Flexible imputation of missing data. Boca Raton, London, New York: CRC Press Taylor & Francis Group A Chapman & Hall Book.
3. Rezvan PH, Comulada WS, Fernández MI, Belin TR (2022) Assessing Alternative Imputation Strategies for Infrequently Missing Items on Multi-item Scales. Communications in statistics. Case studies, data analysis and applications 8(4), 682–713. 10.1080/23737484.2022.2115430.
4. Mainzer RM, Nguyen CD, Carlin JB, Moreno-Betancur M, White IR, Lee KJ (2024) A comparison of strategies for selecting auxiliary variables for multiple imputation. Biometrical journal. Biometrische Zeitschrift 66(1), e2200291. 10.1002/bimj.202200291.
5. van Ginkel JR, Linting M, Rippe RCA, van der Voort A (2020) Rebutting Existing Misconceptions About Multiple Imputation as a Method for Handling Missing Data. Journal of personality assessment 102(3), 297–308. 10.1080/00223891.2018.1530680.
6. Wu W, Jia F, Enders C (2015) A Comparison of Imputation Strategies for Ordinal Missing Data on Likert Scale Variables. Multivariate behavioral research 50(5), 484–503. 10.1080/00273171.2015.1022644.
7. Eekhout I, Vet HCW de, Twisk JWR, Brand JPL, Boer MR de, Heymans MW (2014) Missing data in a multi-item instrument were best handled by multiple imputation at the item score level. Journal of clinical epidemiology 67(3), 335–342. 10.1016/j.jclinepi.2013.09.009.
8. Seaman SR, Bartlett JW, White IR (2012) Multiple imputation of missing covariates with non-linear effects and interactions: an evaluation of statistical methods. BMC medical research methodology 12, 46. 10.1186/1471-2288-12-46.
9. Golino HF, Gomes CMA (2016) Random forest as an imputation method for education and psychology research: its impact on item fit and difficulty of the Rasch model. International Journal of Research & Method in Education 39(4), 401–421. 10.1080/1743727X.2016.1168798.
10. Tutz G (2022) Ordinal regression: A review and a taxonomy of models. WIREs Computational Stats 14(2). 10.1002/wics.1545.
11. Bürkner P-C, Vuorre M (2019) Ordinal Regression Models in Psychology: A Tutorial. Advances in Methods and Practices in Psychological Science 2(1), 77–101. 10.1177/2515245918823199.
12. Bürkner P-C (2017) brms An R Package for Bayesian Multilevel Models Using Stan. J. Stat. Soft. 80(1). 10.18637/jss.v080.i01.
13. Hoffman MD, Gelman A (2014) he No-U-Turn Sampler: Adaptively Setting Path Lengths in Hamiltonian Monte Carlo. http://www.stat.columbia.edu/~gelman/research/published/nuts.pdf.
